# Supplementary material for: Effects of behavioural parent training for children with attention-deficit/hyperactivity disorder on parenting behaviour: a protocol for an individual participant data meta-analysis
Source: BMJ Open. 2020 Nov 27;10(11):e037749. doi: 10.1136/bmjopen-2020-037749 (PMC7703408; doi:10.1136/bmjopen-2020-037749)
Supplement: Supplementary data [file bmjopen-2020-037749supp002.pdf]

Supplement 1- Search terms per database

Behavioral interventions

PubMed
("Attention Deficit Disorder with Hyperactivity"[Mesh] OR ADHD[tiab] OR ADD[tiab] OR attention deficit*[tiab] OR hyperactiv*[tiab] OR hyperkinetic*[tiab] OR minimal brain deficit*[tiab] OR minimal brain dysfunction*[tiab])
AND

(("Psychotherapy"[Mesh] OR psychotherap*[tiab] OR psychological therap*[tiab] OR psychological intervent*[tiab] OR psychoeduca*[tiab] OR mentoring*[tiab] OR coaching*[tiab] OR mindful*[tiab] OR relax*[tiab] OR meditat*[tiab])
OR
((parent*[tiab] OR mother[tiab] OR father[tiab] OR teacher*[tiab] OR school*[tiab])
AND
(program*[tiab] OR train*[tiab] OR educa*[tiab] OR therapy*[tiab] OR therapies*[tiab] OR therapeu*[tiab]OR intervention*[tiab] OR coaching*[tiab] OR counseling*[tiab]))
OR
((behavio*[tiab] OR cognit*[tiab] OR “acceptance and commitment”[tiab] OR dialectica*[tiab])
AND
(program*[tiab] OR therapy*[tiab] OR therapies*[tiab] OR therapeu*[tiab] OR intervention*[tiab] OR treatment*[tiab] OR train*[tiab]))
OR
(psychosocial[tiab]
AND
(treatment*[tiab] OR therapy*[tiab] OR therapies*[tiab] OR therapeu*[tiab] OR train*[tiab] OR intervention*[tiab] OR program*[tiab]))
OR
((skill*[tiab] OR organization*[tiab] OR organisation*[tiab] OR planning*[tiab] OR play*[tiab])
AND
(train*[tiab] OR intervention*[tiab] OR program*[tiab])))

AND

("Adolescent"[Mesh] OR "Child"[Mesh] OR child*[tiab] OR school*[tiab] OR infan*[tiab] OR adolescen*[tiab] OR pediatri*[tiab] OR paediatr*[tiab] OR boy[tiab] OR boys[tiab] OR boyhood[tiab] OR girl[tiab] OR girls[tiab] OR girlhood[tiab] OR youth[tiab] OR youths[tiab] OR teen[tiab] OR teens[tiab] OR teenage*[tiab] OR puberty[tiab] OR preschool*[tiab] OR toddler*[tiab] OR juvenile*[tiab] OR kids[tiab])
AND

("Controlled Clinical Trial" [Publication Type] OR "Randomized Controlled Trials as Topic"[Mesh] OR "Follow-Up Studies"[Mesh] OR follow-up[tiab] OR followup[tiab] OR control group*[tiab] OR "Random Allocation"[Mesh] OR random*[tiab] OR trial[ti])

EMBASE EMBASE.com (losse delen combineren op website)
('attention deficit disorder'/exp OR (ADHD OR ADD OR ‘attention deficit*’
OR hyperactiv* OR hyperkinetic* OR ‘minimal brain deficit*’ OR ‘minimal brain dysfunction*’):ab,ti)
AND

('psychotherapy'/exp OR (psychotherap* OR ‘psychological therap*’ OR ‘psychological intervent*’
OR psychoeduca* OR mentoring* OR coaching* OR mindful* OR relax*):ab,ti)
OR
((((parent* OR mother OR father OR teacher* OR school*)
AND
(program* OR train* OR educa* OR therapy* OR therapies* OR therapeu*OR intervention* OR coaching* OR counseling*))
OR
((behavio* OR cognit* OR ‘acceptance and commitment’ OR dialectica*)
AND
(program* OR therapy* OR therapies* OR therapeu* OR intervention* OR treatment* OR train*))
OR
(psychosocial
AND
(treatment* OR therapy* OR therapies* OR therapeu* OR train* OR intervention* OR program*))
OR
((skill* OR organization* OR organisation* OR planning* OR play*)
AND
(train* OR intervention* OR program*))):ab,ti)

AND
('juvenile'/exp OR (child* OR school* OR infan* OR adolescen* OR pediatri* OR paediatr* OR boy OR boys OR boyhood OR girl OR girls OR girlhood OR youth OR youths OR teen OR teens OR teenage* OR puberty OR preschool* OR toddler* OR juvenile* OR kids):ab,ti)

AND
('controlled clinical trial'/exp OR 'randomization'/exp OR (‘control group*’ OR random*):ab,ti OR trial:ti)

EBSCO PsycINFO
(DE "Attention Deficit Disorder" OR DE "Attention Deficit Disorder with Hyperactivity" OR ADHD OR ADD OR “attention deficit*” OR hyperactiv* OR hyperkinetic* OR “minimal brain deficit*” OR “minimal brain dysfunction*”)
AND
(DE "Psychotherapy" OR DE "Child Psychotherapy" OR DE "Play Therapy" OR DE "Behavior Therapy" OR DE "Aversion Therapy" OR DE "Conversion Therapy" OR DE "Dialectical Behavior Therapy" OR DE "Exposure Therapy" OR DE "Implosive Therapy" OR DE "Reciprocal Inhibition Therapy" OR DE "Response Cost" OR DE "Systematic Desensitization Therapy" AND DE "Cognitive Behavior Therapy" OR DE "Cognitive Therapy" OR DE "Group Psychotherapy" OR DE "Neurotherapy" OR psychotherap* OR “psychological therap*” OR “psychological intervent*” OR psychoeduca* OR mentoring* OR coaching* OR mindful* OR relax*)
OR
((parent* OR mother OR father OR teacher* OR school*)
AND
(program* OR train* OR educa* OR therapy* OR therapies* OR therapeu*OR intervention* OR coaching* OR counseling*))
OR
((behavio* OR cognit* OR ‘acceptance and commitment’ OR dialectica*)
AND
(program* OR therapy* OR therapies* OR therapeu* OR intervention* OR treatment* OR train*))
OR
(psychosocial
AND
(treatment* OR therapy* OR therapies* OR therapeu* OR train* OR intervention* OR program*))
OR
((skill* OR organization* OR organisation* OR planning* OR play*)
AND
(train* OR intervention* OR program*))
AND
(AG (childhood OR adolescence) OR (child* OR school* OR infan* OR adolescen* OR pediatri* OR paediatr* OR boy OR boys OR boyhood OR girl OR girls OR girlhood OR youth OR youths OR teen OR teens OR teenage* OR puberty OR preschool* OR toddler* OR juvenile* OR kids))
AND
(“control group*” OR random* OR "controlled trial" OR "controlled study" OR "experimental study" OR "experimental design" OR TI trial)

EBSCO CINAHL
(MH "Attention Deficit Hyperactivity Disorder" OR "Attention Deficit Disorder with Hyperactivity" OR ADHD OR ADD OR “attention deficit*” OR hyperactiv* OR hyperkinetic* OR “minimal brain deficit*” OR “minimal brain dysfunction*”)
AND
(MH "Psychotherapy+" OR psychotherap* OR “psychological therap*” OR “psychological intervent*” OR psychoeduca* OR mentoring* OR coaching* OR mindful* OR relax*)
OR
((parent* OR mother OR father OR teacher* OR school*)
AND

(program* OR train* OR educa* OR therapy* OR therapies* OR therapeu*OR intervention* OR coaching* OR counseling*))
OR
((behavio* OR cognit* OR ‘acceptance and commitment’ OR dialectica*)
AND
(program* OR therapy* OR therapies* OR therapeu* OR intervention* OR treatment* OR train*))
OR
(psychosocial
AND
(treatment* OR therapy* OR therapies* OR therapeu* OR train* OR intervention* OR program*))
OR
((skill* OR organization* OR organisation* OR planning* OR play*)
AND
(train* OR intervention* OR program*))
AND
(AG child OR (child* OR school* OR infan* OR adolescen* OR pediatri* OR paediatr* OR boy OR boys OR boyhood OR girl OR girls OR girlhood OR youth OR youths OR teen OR teens OR teenage* OR puberty OR preschool* OR toddler* OR juvenile* OR kids))
AND
(MH "Clinical Trials+" OR “control group*” OR random* OR "controlled trial" OR "controlled study" OR "experimental study" OR "experimental design" OR TI trial)

EBSCO ERIC
(DE "Attention Deficit Disorder" OR DE "Attention Deficit Hyperactivity Disorder" OR ADHD OR ADD OR “attention deficit*” OR hyperactiv* OR hyperkinetic* OR “minimal brain deficit*” OR “minimal brain dysfunction*”)
AND

(DE "Psychotherapy" OR psychotherap* OR “psychological therap*” OR “psychological intervent*” OR psychoeduca* OR mentoring* OR coaching* OR mindful* OR relax*)
OR
((parent* OR mother OR father OR teacher* OR school*)
AND
(program* OR train* OR educa* OR therapy* OR therapies* OR therapeu*OR intervention* OR coaching* OR counseling*))
OR
((behavio* OR cognit* OR ‘acceptance and commitment’ OR dialectica*)
AND
(program* OR therapy* OR therapies* OR therapeu* OR intervention* OR treatment* OR train*))
OR
(psychosocial
AND
(treatment* OR therapy* OR therapies* OR therapeu* OR train* OR intervention* OR program*))
OR
((skill* OR organization* OR organisation* OR planning* OR play*)
AND

(train* OR intervention* OR program*))
AND

(child* OR school* OR infan* OR adolescen* OR pediatri* OR paediatr* OR boy OR boys OR boyhood OR girl OR girls OR girlhood OR youth OR youths OR teen OR teens OR teenager* OR puberty OR preschool* OR toddler* OR juvenile* OR kids))
AND

(“control group*” OR random* OR "controlled trial" OR "controlled study" OR "experimental study" OR "experimental design" OR TI trial)

Web of Science
(TS=("Attention Deficit Disorder with Hyperactivity" OR ADHD OR ADD OR “attention deficit*” OR hyperactiv* OR hyperkinetic* OR “minimal brain deficit*” OR “minimal brain dysfunction*”))
AND

(TS=(psychotherap* OR psychotherap* OR “psychological therap*” OR “psychological intervent*” OR psychoeduca* OR mentoring* OR coaching* OR mindful* OR relax*
OR
((parent* OR mother OR father OR teacher* OR school*)
AND
(program* OR train* OR educa* OR therapy* OR therapies* OR therapeu*OR intervention* OR coaching* OR counseling*))
OR
((behavio* OR cognit* OR ‘acceptance and commitment’ OR dialectica*)
AND
(program* OR therapy* OR therapies* OR therapeu* OR intervention* OR treatment* OR train*))
OR
(psychosocial
AND
(treatment* OR therapy* OR therapies* OR therapeu* OR train* OR intervention* OR program*))
OR
((skill* OR organization* OR organisation* OR planning* OR play*)
AND
(train* OR intervention* OR program*))))
AND
(TS=(child* OR school* OR infan* OR adolescen* OR pediatri* OR paediatr* OR boy OR boys OR boyhood OR girl OR girls OR girlhood OR youth OR youths OR teen OR teens OR teenager* OR puberty OR preschool* OR toddler* OR juvenile* OR kids))
AND
(TS=(“clinical trial*” OR “control group*” OR random* OR "controlled trial" OR "controlled study" OR "experimental study" OR "experimental design"))

Cochrane Central register of Controlled Trials *(CENTRAL)*
("Attention Deficit Disorder with Hyperactivity" OR ADHD OR ADD OR “attention deficit*” OR hyperactiv* OR hyperkinetic* OR “minimal brain deficit*” OR “minimal brain dysfunction*”)
AND

((psychotherap* OR “psychological therap*” OR “psychological intervent*” OR psychoeduca* OR mentoring* OR coaching* OR mindful* OR relax*)
OR
((parent* OR mother OR father OR teacher* OR school*)
AND
(program* OR train* OR educa* OR therapy* OR therapies* OR therapeu*OR intervention* OR coaching* OR counseling*))
OR
((behavio* OR cognit* OR ‘acceptance and commitment’ OR dialectica*)
AND
(program* OR therapy* OR therapies* OR therapeu* OR intervention* OR treatment* OR train*))
OR
((psychosocial)
AND
(treatment* OR therapy* OR therapies* OR therapeu* OR train* OR intervention* OR program*))
OR
((skill* OR organization* OR organisation* OR planning* OR play*)
AND
(train* OR intervention* OR program*)))
AND

(child* OR school* OR infan* OR adolescen* OR pediatri* OR paediatr* OR boy OR boys OR boyhood OR girl OR girls OR girlhood OR youth OR youths OR teen OR teens OR teenage* OR puberty OR preschool* OR toddler* OR juvenile* OR kids)

Supplement 2. Data sharing manual

Data collection form

Content

Introduction
How do I transfer my data?
Which participants do we need?
Which data do we need?
What will we do with the data?
Supplement 3A. Measures

Thank you for taking place in our Psychosocial ADHD INTerverntions (PAINT) collaborators group. We are very grateful that you are willing to put your time into this project. We believe that this group will be fruitful and successful collaboration with the aim to get more insight into psychosocial interventions for ADHD.
With this manual, we hope to provide you with guidance on the data collection. However, if you have any questions regarding the data handling, despite reading this manual, please let us know so we can help you further, and adapt our manual accordingly. You can reach us at paint@accare.nl.

Most of the choices we have made concerning data collection are driven by a need to keep methods and measures similar across trials. Every study is designed slightly different, with different measures, different in- and exclusion criteria, different methods to handle missing data, etc..
To harmonize measures and methods, at some point we will have to make choices regarding data analysis, that differ from the choices made in the original study. For example, we will do intention to treat analysis, while it may be possible that you did not do this in your original analysis. This means that, if available, we will ask for data or participants not included in the original analysis. Furthermore, it is important to mention that we will not report on results from a single trial, although we can report a forestplot displaying all effect sizes of included studies.

How do I transfer my data?
Preferably, we would receive the information electronically. We can work with any kind of data file (STATA, SPSS, etc.), but we prefer a layout, where each line represents a subject.

You can email the data file once you encrypted it using a program such as 7-zip (an explanation on how to do this is included in supplement B). It is important that the password with which you will protect your data file, will be send in a separate email from the email that contains the data file (as attachment). Another important point in transferring the data is that the data should be anonymized. This means that there is no information with which we could trace a single participant back to the individual. This means that no names should be included in the file. However, all other information that can identify a person should be left out of this file. This can mean combinations of variables, for example postal code and date of birth. Please make sure that this kind of information is not present in the data file. If these kinds of examples arise, please contact us, so we can think with you on what best to do.

Which participants do we need?
We want the data of all included participants. This means all participants that have a baseline measure and were randomized. It could be possible that this is a different approach than you took in

your analysis of the data. Because we want to perform intention to treat analysis, we need all data from included participants.

Which data do we need?

We ask you for clean data (please contact us if this provides you with any questions). We want sum scores and if available norm-scores of questionnaires. Proper labeling is important. In the Table below we provide you with an overview of constructs that we are interested in. It could be possible that the measure or instrument used in your study is not mentioned in this Table, this does not mean that we are not interested, the measures mentioned are mere examples. If there are any remarks on this measure (e.g. certain items were not used) please mention these in the column "remarks". Please indicate which informants filled out the questionnaires, when multiple informants are used please provide us with data from all informants. If multiple instruments have been used to measure a certain construct, we would really like to receive all instruments (for example a self and a proxy version, or multiple ADHD measures). This could aid us in the harmonization process (see above). If a construct is measured with an in-house instrument or if you made any adjustments on the instrument, could you also provide us this instrument?

For questionnaires, we would like to receive the, sum scores and norm-scores and if available item scores. We would always like to receive the full questionnaire if possible, and not just certain subscales. In the future, it could be possible that we will ask you about norming tables, we hope however, that we will not need these.

For (structured) interviews we would like to receive items scores, sum scores and outcomes. We would like to receive information on the full interview, not only certain outcomes.

Concerning cognitive measures, we would like to receive calculated outcomes variables across trials and conditions, not mere trial to trial performance. For example, in the stop task, we would like to receive measures such as SSRT, mean (and SD) of reaction time on Go trials, number of commissions and omissions, but not the individual performance on a single trial of the stop task. We fill also need information about the task such as number of conditions, number of trials, and how the outcome variables were calculated.

For variables that are available on multiple time points, please provide us with all relevant data. For example, if an ADHD measure is taken at baseline, right after the intervention and after 3 months, we would like to receive this measure assessed at all time points. Again, proper labelling is very important. Please label the data in English (or Dutch).

The constructs and variables that we are interested in for the IPD meta-analysis are shown below. We understand that not all studies have included all measures. The table below will provide you with a list you can keep on hand when preparing the data before sending it. Next to the constructs are examples of instruments. Please remember that this is not a complete list, and that it could be possible that you have used an instrument that is not in our list, but that would still be very valuable for this IPD meta-analysis. Please remember that you do not have to recode your data. We will do this for all datasets.

What will we do with the data?

Once we receive the data we will do some basic data checks. These will include checking missing and impossible values. If we will have any questions concerning the data during or after these checks we will contact you for clarification. After that we will harmonize the data with the other datasets. This harmonization process depends on the available data. For example, this could mean that we have to dichotomize data, or making norm deviation scores (translating raw questionnaire scores on one

scale into raw questionnaire scores on another scale using norm scores). Harmonization is easiest if the same measure has been used across studies. If you have used multiple instruments to measure a certain outcome (e.g. more than 1 ADHD scale), we ask you to provide us both measures. This could mean that we may be able use a latent variable for some measures, or we could calculate correlations between measures.

The original dataset will be saved as the original file. Any harmonization will take place in a separate file, which are only later merged into one big data file.

Supplement 2A.

Tabel 1 Measures.

**Descriptive measures**	
Participant ID	
Date of randomization	Dd/mm/yyyy
Date of treatment start	Dd/mm/yyyy
Date post-intervention measurement	Dd/mm/yyyy
Date follow up measurement	Dd/mm/yyyy
Date baseline measurement	Dd/mm/yyyy
Treatment allocation	0- Control<br>1- Intervention
Age	Age in years
Gender	1-Male<br>2-Female
IQ	Norm score of WISC
Verbal IQ	Norm score of WISC
Performance IQ	Norm score of WISC
Ethnicity	
Socioeconomic status	
ADHD severity at baseline	
Comorbidities	ODD/CD/Anxiety/Depression/PDD/Tics/ etc.
Number of children living with the family	Total number of children in the household
Primary care-taker education	
Secundary care-taker education	
Household income	Combined income of primary caretaker and (if applicable) secundary caretaker
Parental psychopathology (diagnosis)	Parental ADHD/ anxiety/depression/ etc
Marital status	
Education primary caretaker	
Education secondary caretaker	
Age Father	Age in years
Age mother	Age in years

Psychoactive Medication use pre- intervention	Yes/no, if yes which medication, mg MPH if applicable, x times per day, for the last month, duration
Previous care use of the child	Care use in mental health.
Medication use during intervention	Yes/no, if yes, mg per day
Treatment expectancy	
Stable medication use during intervention	Yes/No/NA
Other descriptive measures	
**Individual child outcomes- behavioral**	
**Measured constructs**	**Examples of instruments**
**ADHD**	Conners, CBCL, DBD, ACTeRS, ARS, BASC, SNAP, SWAN, Werry Weiss Peters activity scale, Behavioral checklist, PACS, Parent daily report, KSADS, standardized behavioral observations
Disruptive disorders/ behavior	Conners, CBCL, DBD, EDBI, SNAP, SSRS, ACTeRS, KSADS, behavior problem checklist, aggression and conduct problems scale, behavioral checklist, PACS, parent daily reports, standardized behavioral observations
Social skills	DSAS, Behavior problem checklist, BASC, ACTeRS, SSRS, CBCL
Internalizing problems	Conners, CBCL, SSRS, BASC, Behavior problem checklist, MASC, PACS, KSADS, SCARED, CDI
Attachment	
Quality of life	HONOSCA, kidsscreen, EQ-5d, IRS
Selfconcept	Piers-Harris
Which other child behavioral outcomes are available?	
**Individual child outcomes- academic outcomes**	
Measured Construct	Examples of measures
Grades on core courses	Math, Spelling, Reading
Standardized Academic Achievement tests	Reading, spelling, math scores, woodcock johnson, WRAT, WISC Durrel
On and off task behaviors	Classroom observations, SOAPs
Academic competence	Academic rating scale, classroom performance survey

Which other academic outcomes are available?	
**Individual child outcomes- cognitive outcomes[a]**	
Higher cognitive functions	Working memory, executive functions, inhibition, planning, set shifting, reward sensitivity, emotion regulation
Intellectual functioning	IQ (WISC, RAVEN)
Motor skills	Fine motor skills, precision, gross motor skills
Memory	Visuospatial, verbal
Basic cognitive functions	Attention, reaction time, speed, perception
**Child individual outcomes- other measures**	
Adverse events	Types of adverse events reported
Treatment fidelity	
Treatment satisfaction	
Number of sessions attended	
Drop out	Yes/no
Date of discontinuation of the intervention	
Reason for discontinuation	
**Parental outcome measures**	
Measured constructs	Examples of measures
Parental psychopathology	DASS, GHQ, BDI, PSI, CSS, AARS, SCL90,
Parent child interaction, parenting behaviors, parenting style, parenting affect	DPICS or other observational measure, parenting practices inventory (PPI), parent interaction style, PS (parenting scale), measures parental sensitivity/reponsivity
Parental competence/ self efficacy	PSI-subscale parental competence, PSOC
Spouse relationship	locke wallace marital adjustment, PSI-subscale
Parental self esteem	Rosenberg self esteem scale (RSE)
Parenting stress	PSI
Which other parenting outcomes are available?	

[a] concerning cognitive measures, we would like to receive calculated outcomes measures, not mere trial to trial performance. For example, in the stop task, we would like to receive measures such as SSRT, mean reaction time, number of commissions and omissions, but not the individual performance on a single trial of the stop task. We will also need information on the conditions of the task, how the outcome was calculated, etc.
