# Supplementary material for: Effects of behavioural parent training for children with attention-deficit/hyperactivity disorder on parenting behaviour: a protocol for an individual participant data meta-analysis
Source: BMJ Open. 2020 Nov 27;10(11):e037749. doi: 10.1136/bmjopen-2020-037749 (PMC7703408; doi:10.1136/bmjopen-2020-037749)
Supplement: Author's manuscript [file bmjopen-2020-037749.draft_revisions.pdf]

BMJ Open

BMJ Open is committed to open peer review. As part of this commitment we make the peer review history of every article we publish publicly available.

When an article is published we post the peer reviewers' comments and the authors' responses online. We also post the versions of the paper that were used during peer review. These are the versions that the peer review comments apply to.

The versions of the paper that follow are the versions that were submitted during the peer review process. They are not the versions of record or the final published versions. They should not be cited or distributed as the published version of this manuscript.

BMJ Open is an open access journal and the full, final, typeset and author-corrected version of record of the manuscript is available on our site with no access controls, subscription charges or pay-per-view fees (http://bmjopen.bmj.com).

If you have any questions on BMJ Open's open peer review process please email info.bmjopen@bmj.com

BMJ Open

# Effects of Behavioral Parent Training for children and adolescents with attention-deficit/hyperactivity disorder on Parenting Behavior: A protocol for an Individual Participant Data Meta-analysis


| | |
|---|---|
| Journal: | *BMJ Open* |
| Manuscript ID | bmjopen-2020-037749 |
| Article Type: | Protocol |
| Date Submitted by the Author: | 14-Feb-2020 |


| | |
|---|---|
| Complete List of Authors: | Steenhuis, Laura; University of Groningen, Department of Child and Adolescent Psychiatry<br>Groenman, Annabeth; University Medical Center Groningen, Accare, child and adolescent psychiatry<br>Hoekstra, Pieter J; University of Groningen<br>Hornstra, Rianne; University Medical Center Groningen, Accare, child and adolescent psychiatry<br>Luman, Marjolein; VU medisch centrum,<br>van der Oord, Saskia; KU Leuven, Clinical Psychology<br>van den Hoofdakker, Barbara; University Medical Center Groningen, Accare, child and adolescent psychiatry |


| | |
|---|---|
| Keywords: | Child & adolescent psychiatry < PSYCHIATRY, MENTAL HEALTH, PAEDIATRICS, PSYCHIATRY |
| | |


SCHOLARONE™
Manuscripts

*I, the Submitting Author has the right to grant and does grant on behalf of all authors of the Work (as defined in the below author licence), an exclusive licence and/or a non-exclusive licence for contributions from authors who are: i) UK Crown employees; ii) where BMJ has agreed a CC-BY licence shall apply, and/or iii) in accordance with the terms applicable for US Federal Government officers or employees acting as part of their official duties; on a worldwide, perpetual, irrevocable, royalty-free basis to BMJ Publishing Group Ltd ("BMJ") its licensees and where the relevant Journal is co-owned by BMJ to the co-owners of the Journal, to publish the Work in this journal and any other BMJ products and to exploit all rights, as set out in our licence.*

*The Submitting Author accepts and understands that any supply made under these terms is made by BMJ to the Submitting Author unless you are acting as an employee on behalf of your employer or a postgraduate student of an affiliated institution which is paying any applicable article publishing charge ("APC") for Open Access articles. Where the Submitting Author wishes to make the Work available on an Open Access basis (and intends to pay the relevant APC), the terms of reuse of such Open Access shall be governed by a Creative Commons licence – details of these licences and which Creative Commons licence will apply to this Work are set out in our licence referred to above.*

*Other than as permitted in any relevant BMJ Author's Self Archiving Policies, I confirm this Work has not been accepted for publication elsewhere, is not being considered for publication elsewhere and does not duplicate material already published. I confirm all authors consent to publication of this Work and authorise the granting of this licence.*

For peer review only

# Effects of Behavioral Parent Training for children and adolescents with attention-deficit/hyperactivity disorder on Parenting Behavior: A protocol for an Individual Participant Data Meta-analysis

**Laura A. Steenhuis°**, University of Groningen, University Medical Center Groningen, Department of Child and Adolescent Psychiatry, Groningen, the Netherlands
**Annabeth P. Groenman°,** University of Groningen, University Medical Center Groningen, Department of Child and Adolescent Psychiatry, Groningen, the Netherlands
**Pieter J. Hoekstra,** University of Groningen, University Medical Center Groningen, Department of Child and Adolescent Psychiatry, Groningen, the Netherlands
**Rianne Hornstra,** University of Groningen, University Medical Center Groningen, Department of Child and Adolescent Psychiatry, Groningen, the Netherlands
**Marjolein Luman*,** Department of Clinical Neuropsychology, Vrije Universiteit Amsterdam, Amsterdam, The Netherlands. Bascule, academic centre for child and adolescent psychiatry
**Saskia vd Oord*,** Department of Clinical Psychology, KU Leuven; Developmental Psychology, University of Amsterdam
**Barbara J. vd Hoofdakker*,** University of Groningen, University Medical Center Groningen, Department of Child and Adolescent Psychiatry, Groningen, the Netherlands; University of Groningen, Department of Clinical Psychology and Experimental Psychopathology, Groningen, The Netherlands

**° shared first author, authors made equal contributions to the project.**
* Shared last author, authors made equal contributions to the project.

**Corresponding Author:** Annabeth P. Groenman,

Address: Accare, Department of Child and Adolescent Psychiatry, Freepost 176, 9700 VB Groningen, the Netherlands

E-mail address: a.groenman@accare.nl

**Word count main body: 3999 [max 4000]**
**Word count abstract: 291 [max 300]**



For peer review only - http://bmjopen.bmj.com/site/about/guidelines.xhtml

**Conflicts of interest:**
APG, LAS, PJH, RH, ML, SVDO, BvdH have no conflicts of interest to report.

**Funding:** This research was funded by The Dutch Organization for Health Research and Development (ZonMw) under grant number 729300013. The funder had no role in the design of this protocol.The funder will have no input on the collection of data, the data analysis or the interpretation or publication of the study results.

**Author contributions:** BvdH is the guarantor. APG wrote the first draft of the manuscript, and LAS wrote a revised and final version. APG, RH and LAS are performing the systematic search and data extraction. PJH, ML, SvdO, BvdH acquired funding for the PAINT-IPDMA project; an international database including individual participant data from RCTs examining psychosocial interventions for the treatment of ADHD in children. The current study is a part of the PAINT-IPDMA project. PJH, ML, SvdO, BvdH, and APG setup the initial design for the IPDMA. All authors worked together to design this IPDMA, contributed to the intellectual content of the current manuscript and approved the final version.

**Acknowledgements**
We would like to thank all collaborators of the PAINT-IPDMA project for providing input to this protocol and for providing data for this project.

**Exclusive License**
I, the Submitting Author has the right to grant and does grant on behalf of all authors of the Work (as defined in the below author licence), an exclusive licence and/or a non-exclusive licence for contributions from authors who are: i) UK Crown employees; ii) where BMJ has agreed a CC-BY licence shall apply, and/or iii) in accordance with the terms applicable for US Federal Government officers or employees acting as part of their official duties; on a worldwide, perpetual, irrevocable, royalty-free basis to BMJ Publishing Group Ltd ("BMJ") its licensees and where the relevant Journal is co-owned by BMJ to the co-owners of the Journal, to publish the Work in BMJ Open and any other BMJ products and to exploit all rights, as set out in our licence.

The Submitting Author accepts and understands that any supply made under these terms is made by BMJ to the Submitting Author unless you are acting as an employee on behalf of your employer or a postgraduate student of an affiliated institution which is paying any applicable article publishing charge ("APC") for Open Access articles. Where the Submitting Author wishes to make the Work available on an Open Access basis (and intends to pay the relevant APC), the terms of reuse of such Open Access shall be governed by a Creative Commons licence – details of these licences and which Creative Commons licence will apply to this Work are set out in our licence referred to above.

For peer review only



For peer review only - http://bmjopen.bmj.com/site/about/guidelines.xhtml

**Abstract**

Introduction: Behavioral Parent Training (BPT) is a well-established treatment for children with attention-deficit/hyperactivity disorder (ADHD). BPT is based on the hypothesis that improvements in parenting are mediators of improvements in children's behaviors. However, meta-analyses show considerate heterogeneity in effects of BPT on child outcomes, and meta-analyses on parenting outcomes are scarce. Also, few studies have investigated parenting factors as mediators of child outcomes. This study aims to examine the effects and moderators of BPT on parenting outcomes and whether improvements in parenting mediate amelioration of behavior and impairment in children and adolescents with ADHD.

Methods and analyses: We will conduct an individual participant data meta-analysis (IPDMA), making use of individual data of existing trials, and giving the opportunity for highly powered moderator analyses. This IPDMA will be performed by the Psychosocial ADHD INTervention (PAINT) collaboration. We will include randomized controlled trials of BPT, for individuals with ADHD below 18 years old. Systematic searches have been performed to locate relevant papers. Authors are currently contacted to share their data with the PAINT-IPDMA project. We will examine effects of BPT on parenting outcomes (e.g., positive and negative parenting, management of affect, perceived parenting competence, parenting stress), moderators of these effects (e.g., parental depression, parenting stress, severity of the child's ADHD symptoms) and subsequently perform mediation analyses where parenting outcomes are modelled as mediators of child outcomes (e.g., symptoms and severity of ADHD, comorbid psychopathology and impairment).

Ethics and Dissemination: We will include data from RCTs for which ethical approval has been received and consent forms have been signed. De-identified data will be provided by the original investigators. We aim to disseminate through peer-reviewed scientific journals, presentations at (inter)national scientific meetings, newsletters, the website of our project and the Dutch academic workspace ADHD.



For peer review only - http://bmjopen.bmj.com/site/about/guidelines.xhtml

Article summary

*Strengths and limitations of this study:*

- There is a need for well powered studies into the effects of behavioral parent training for ADHD on parenting outcomes, which is the aim of the current IPDMA.
- We will examine potential moderators (such as parental depression, parenting stress and ADHD severity) of parenting outcomes.
- We will investigate whether changes in parenting mediate treatment outcomes of children's ADHD and behavioral symptoms.
- Our IPDMA is limited to the examination of those variables that are reported consistently across all included studies.

For peer review only

**Introduction**

Behavioral parent training (BPT) is a well-established intervention for children with attention-deficit/hyperactivity disorder (ADHD) and recommended as first line treatment by many international guidelines [1–3]. In BPT, parents are trained to apply behavioral techniques meant to increase prosocial and adaptive child behaviors and to reduce disruptive and maladaptive child behaviors. In addition, BPT focusses on nurturing and positive parent-child relationships [4]. Several meta-analyses on BPT for children with ADHD have shown medium to large effect sizes on parent reported reductions of ADHD symptoms, comorbid problems, impairment and parenting behaviors [3,5–7]. However, the full range of relevant parenting outcomes of BPT has not been assessed in meta-analyses [7,8]. Moreover, while improvements in parenting are thought to mediate improvements in children's behaviors and associated impairments [3,9], studies investigating these mediation effects are scarce. Another problem is that individual studies commonly lack the statistical power to adequately assess moderators of improvements of parenting outcomes. Knowing which moderators impact the effectiveness of BPT for child [5] and parenting outcomes [3,6], in addition to knowing which mediators drive the effects of BPT will give more insight into for whom BPT works best and the working mechanisms underlying this, therefore contributing to personalized treatment of children with ADHD. Also, more insight is needed regarding the effects of BPT on different parenting outcomes.

BPT aims to increase positive parenting and reduce negative parenting behaviors, improve management of affect, reduce parenting stress and enhance parenting self-efficacy [9], in order to positively influence children's behaviors and decrease impairment. Parents are trained to modify environmental antecedents (e.g., providing structure) and consequences (e.g., positive rewards for adaptive behavior). Positive parenting consists of praise, encouragement, effective communication, positive affect and physical affirmations [10,11], whereas negative parenting is described as inconsistent discipline, verbal criticism, corporal punishment, poor supervision and negative affect [12–14]. Essential to positive parenting is the effective management of parental affect, which is often a specific target of BPT [15]. Parents are taught to express positive emotions (e.g., love, affection and warmth) and to inhibit expressions of negative and unsupportive emotions (e.g., anger, frustration and irritability) [16]. Parenting stress (i.e. stress arising from the feeling that the demands of parenting outweigh the resources [17]) is often reported by parents of children with ADHD [18] and is also an important target of BPT. Lastly, parenting self-efficacy is an important target of BPT [19]; parents should feel more confident and competent in carrying out their parenting tasks.

When assessing parenting outcomes of BPT, it is important to consider a range of outcomes, particularly positive and negative parenting, management of affect, parenting stress and parenting



For peer review only - http://bmjopen.bmj.com/site/about/guidelines.xhtml

self-efficacy. However, treatment trials of BPT have not uniformly assessed all parenting outcomes that are explicit targets of BPT [3,6]. Recently, two meta-analyses examined the effectiveness of behavioral interventions (mostly consisting of BPT) on parenting outcomes in ADHD, either assessed by raters unblinded to the treatment condition (e.g. parents involved in BPT [9]) or by blinded raters (e.g. independent raters coding video-taped interactions between the parent and child [3,6]). The first meta-analysis [3] considered effects on positive and negative parenting and parenting self-efficacy, immediately post-intervention. Results indicated improvements in positive parenting (effect size (ES) of 0.68 and 0.63 for unblinded and blinded raters, respectively), reductions in negative parenting (ES of 0.57 and 0.43 for unblinded and for blinded raters, respectively), and improvements in parenting self-efficacy (ES of 0.37 for unblinded raters). The other meta-analysis [6] investigated the effects of BPT for preschool children with (or at risk for) ADHD, and reported ESs of 0.63 and 0.33 by unblinded and blinded raters, respectively, for reductions in negative parenting. In addition, long-term effects (up to 12 months) on negative parenting was reported by unblinded raters with an ES of 0.12. Overall, both unblinded and blinded parent outcomes show improvements after BPT, although the effects on blinded outcomes generally appeared smaller than the effects on unblinded outcomes. Improvements in parenting stress and parental affect following BPT have not been addressed in meta-analyses. Moreover, individual studies demonstrate that parenting stress can be effectively alleviated with BPT [19], and that mothers who participated in BPT have less negative affect and better emotion regulation abilities post-treatment [20].

Heterogeneity in effect sizes is common in meta-analyses investigating parenting outcomes of BPT for children with ADHD [3,6]. More knowledge on factors associated with treatment effects will yield more insight into for whom BPT works best, and allows clinicians to make better treatment choices tailored to individuals. So far, very few randomized control trials (RCTs) were adequately powered for moderator analyses, and existing moderator analyses were mostly limited to child behavioral outcomes [21–23]. While traditional methods of addressing heterogeneity in meta-analyses (such as excluding extreme ESs, subgroup analyses, or meta-regression) may resolve heterogeneity (i.e. reduce $I^2$), they reveal little about the cause of this heterogeneity. Individual participant data meta-analysis (IPDMA) includes data at an individual level rather than at study level, which enables exploration of moderators, therefore yielding more information about the cause of heterogeneity. Moreover, IPDMA performs a uniform analysis across all studies. IPDMA also has enough power to perform subgroup analyses, which most individual RCTs lack. Due to the collaborative nature of an IPDMA, collaborators can provide input on all phases of the research (including design, analyses, interpretation and manuscript preparation), leading to a high quality product [24–26]. So far, no IPDMA has been conducted for parenting outcomes of BPT for children with ADHD.



For peer review only - http://bmjopen.bmj.com/site/about/guidelines.xhtml

In this paper, we present our protocol for an IPDMA on BPT for children with ADHD. The current IPDMA will explore several child- and parent-related moderators on parenting outcomes of BPT. Given that the examination of moderators on parenting outcomes is scarce, the choice of possible moderators to be investigated in this IPDMA will also be drawn from BPT trials on child outcomes. First, age of the child might be an important moderator [27], as parents may have more influence on younger children, and younger children may have less engrained symptoms. A meta-analysis indeed demonstrated that BPT had more effect on positive parenting for younger children with ADHD [3]. Second, medication use of the child may moderate BPT outcomes, as parents of children on medication might find it easier to adapt their parenting styles and experience less resistance when doing so, although there could also be a floor effect as that children with medication might already function better. Previous studies indeed suggested that medication use may positively contribute to BPT outcomes on child's ADHD symptoms [28], although results have been mixed [21]. Third, it seems plausible that intelligence of the child is positively associated with a treatment that involves learning associations between behaviors and consequences and new skills [29]. There is some evidence that higher children's IQ is associated with more improvement in ADHD symptoms following behavioral treatment, but only for specific subgroups of children, such as anti-social girls [23,30,31]. Fourth, pretreatment ADHD severity and presence of comorbidities could moderate BPT, as parents might find it easier to change their parenting behavior when the child has less complex symptomatology. Previous individual studies have indeed confirmed that comorbidities at baseline negatively impacted the outcome of BPT treatment with regard to child symptoms [21,22,32]. In addition, a recent IPDMA by our group [5] confirmed that comorbidities are associated with worse outcomes for the child in behavioral treatments for ADHD.

There are several parent-related moderators that may have an effect on parenting outcomes of BPT. First, parental mental health problems (depression, ADHD, parental stress) are likely to affect how well parents are able to grasp new information and impact their ability to learn new methods of parenting and skills [21,22,33–35]. Second, low socio-economic status (SES) and limited parental education may moderate outcomes of BPT, as this may increase family strain and impact the availability of family resources. There is mixed evidence of the moderating effect of low SES on the effects of BPT on ADHD symptoms and related problems, showing either no effect [23], or better outcomes for parents with lower education [36]. Effects of low SES in relation to parenting outcomes of BPT have not been investigated. Third, single parenthood may also moderate outcomes of BPT, as individual studies have demonstrated that single mothers are less likely to respond to BPT [37,38], and maintain treatment gains over time [39] in terms of parenting behavior. Fourth, there is evidence that lower parenting self-efficacy at baseline has a negative impact on improvements in behavioral problems in children with ADHD following BPT [40] and one could expect similar results



For peer review only - http://bmjopen.bmj.com/site/about/guidelines.xhtml

for parenting outcomes. On the other hand, it is also possible that lower parenting abilities at baseline yields more room for improvement in BPT, and thus both directions of the effect could be expected.

Following the investigation of parenting outcomes of BPT as part of our IPDMA, the next step will be to examine mediators of improvements in child's behavior following BPT. Currently, there are no meta-analyses analyzing whether improvements in parenting mediate improvements of BPT on symptoms of ADHD of the child. Some evidence of individual studies shows that reductions in parenting or parenting attributions mediate improvements on outcomes in behavioral interventions [12,34,41]. This IPDMA aims to synthesize the available data regarding the association between improvements in parent outcomes and improvements in child's outcomes following BPT.

The specific aims of our IPDMA include:

(1) to investigate effects of BPT on parenting outcomes (positive and negative parenting, parental affect, parenting stress and parenting self-efficacy). Given that BPT for ADHD has shown different effects when assessed by unblinded raters and by more blinded raters [3,6,7], we aim to distinguish (if possible) between unblinded and blinded assessments of parenting and parental affect;

(2) to investigate possible child (including age, medication use, IQ, ADHD severity, presence and severity of comorbidities) and parent (including depression, ADHD, socio-economic status, single parenthood, and parenting measures) moderators of parenting outcomes;

(3) to investigate whether improvements in parenting mediate the effect of BPT on behavioral child outcomes following treatment.

For aims 1-3, outcomes of BPT will be examined immediately post-treatment and at long-term where possible.

**Methods and Analysis**

For this IPDMA we will build on the Psychosocial ADHD INTervention IPD (PAINT-IPD) database which is registered in prospero: https://www.crd.york.ac.uk/PROSPERO/display_record.php?ID=CRD42017069877&ID=CRD42017069877. In this project we collect data on psychosocial treatments for children with ADHD: the search is regularly updated and the database continues to expand. For the purpose of the current IPDMA on parenting outcomes, we will use the same methods and search strategy. This protocol is written in line with the PRISMA-P 2015 checklist [42] (see supplementary checklist). The current study is planned to commence April 2020.

Inclusion criteria



For peer review only - http://bmjopen.bmj.com/site/about/guidelines.xhtml

We will include RCTs of behavioral treatments of individuals aged below 18 with ADHD, corroborated by clinical cutoffs on questionnaires or (semi)-structured interviews. We will include studies that compared BPT with a control condition (i.e., all conditions that are labeled control, including active treatment), and studies that compared BPT to another behavioral intervention (head to head comparisons). We excluded studies or intervention arms that used optimized medication treatment next to BPT as part of their study design or as a control condition. We define BPT as interventions directed at changing children's/adolescents' behaviors (i.e., increasing desirable behaviors and decreasing undesirable behaviors), using (cognitive) behavioral therapeutic techniques which parents are trained in [43]. Multimodal interventions (consisting of both parent and/or teacher and/or child training), will be included if the time spent on parent training within the intervention was at least equal to other types of training.

Selection and screening of studies

The last systematic search was performed on August 1st 2019. Currently, we are contacting the authors of the newly identified studies. Two authors (AG and RH) performed the selection and screening of studies, disagreement was resolved by consensus. A two-step approach to identifying relevant articles was used. First, Medline, CINAHL, PsycINFO, EMBASE+EMBASE CLASSIC, ERIC, Web of Science [Science Citation Index Expanded] was searched for relevant papers using a combination of the following search terms and their synonyms, as well as hierarchical family form (e.g. MeSH terms): treatment specific terms (e.g., behavioral treatment, psychosocial treatment, and parent training), ADHD, child and adolescent, and randomized controlled trial. No date restrictions were applied. English, German and Dutch language publications published in peer-reviewed journals were included. Second, literature lists of all selected studies and relevant systematic reviews and meta-analyses were hand-searched to identify possible missing articles (complete search criteria for each database are available in Supplement 1).

**Data collection and management**

Author contact

We will contact the corresponding authors of all eligible trials to ask for their participation in the current IPD meta-analyses. If after several weeks we have had no response, we will send a reminder to the corresponding author. If we have failed to establish contact with the corresponding author, we will email the other authors of the study. Furthermore, we will contact researchers during conferences, and through our personal network to retrieve all eligible databases.

Data format and management



For peer review only - http://bmjopen.bmj.com/site/about/guidelines.xhtml

Part of the data has already been received in light of our previous IPDMA [5]. For newly identified data that we have not yet received, we will use the best, safest way to transfer the data. The most convenient way for most authors will be to transfer the encrypted data per email to the project management, but we will be open to other options (e.g. face to face transfer). A copy of our data collection manual for the PAINT-IPD database can be found in Supplement 2. We will allow authors to send the data in all possible formats, although the most preferable format would be one in which each subject represents a row and each variable a column.

If authors are not included in the PAINT collaborators group yet [5], we will offer one or two authors of each included study a place in our PAINT collaborators group. We plan yearly telephone calls and/or meetings at large international conferences to keep the working group up to date and discuss design and methodological issues. Members of the working group who have provided data on BPT trials with parenting outcomes will have the opportunity to provide feedback on the first draft of the manuscript and will be sent a copy of the final manuscript before submission. Only authors in the IPD steering committee group (APG, BvdH, SvdO, ML and PH) will have access to the data.

## Ethics and Dissemination

The original investigators will be asked for de-identified data, so that only the original investigator knows the link between data and participant. We will only include RCTs where ethical approval has been given and participants signed consent forms. Since the current IPDMA is an extension of the original purpose of the eligible studies, we do not expect any ethical issues with the current IPDMA.

An expert panel consisting of parents of children with ADHD was organized at the conception of the idea in which feedback was given to the plans and changes were made accordingly. Results of our study will be disseminated through peer-reviewed scientific journals and presentations and (inter)national scientific and/or clinical expert meetings. Our results will be communicated to clinicians, clients and their parents through newsletters, and through the academic workplace “ADHD en druk gedrag” (also see https://adhdendrukgedrag.nl/) in which many parents, client organizations, and mental health care professionals are represented.

## Variables

For the PAINT-IPD database, a data request form will be sent to all authors of the original studies, containing a list of variables that will be requested (see Supplement 2a). This list was determined by reviewing the literature and the IPDMA steering committee (authors APG, SvdO, ML , PH and BvdH) assessed these outcomes domains for suitability and interest. The final list of variables will depend on the available data of all studies.



For peer review only - http://bmjopen.bmj.com/site/about/guidelines.xhtml

For the current protocol, the following parenting variables will be selected:

*Positive and Negative Parenting* will be assessed using unblinded and blinded measures. For unblinded parenting measures, we will select questionnaires such as the Alabama Parenting Questionnaire [10]. For blinded measures, we will select assessments used to observe and code parent behavior, such as the Dyadic Parent-Child Interaction Coding System [44].

*Positive and Negative Parental Affect* will be assessed with measures specifically designed to capture parental affect (e.g. 5 min speech sample of expressed emotion; [15]) or a subscale of an existing parenting scale (e.g. attachment domain of the Parenting-Relationship Questionnaire [45]).

*Parental Stress* will be assessed using measures specifically designed to capture stress from parenting, such as the Parenting Stress Index (PSI; [46]), but also measures assessing stress in the caregiver more generally (Depression and Anxiety Stress Scales, DASS; [47]).

*Parenting Self-Efficacy* will be assessed using measures specifically designed to capture parenting self-efficacy, such as the Parental Sense of Competence Scale (PSOC; [48]), a subscale of an existing parenting scale (e.g. the Parenting-Relationship Questionnaire, [45]), or a scale designed to assess parenting competence in specific contexts, such as education (e.g. Parent as Educator Scale, [49]).

*Parental Depression* will be assessed using measures to capture depressive symptoms, such as the Beck Depression Inventory (BDI; [50]), a subscale of an existing mental health questionnaire (e.g. depression subscale of the Depression and Anxiety Stress Scales, DASS; [51]) or a mental health questionnaire for which the overall score can be used as a proxy for depression (e.g. the General Health Questionnaire, GHQ; [52]).

*Parental ADHD* will be assessed using an adult measure of ADHD, such as the Adult Self-Report Scale (ASRS) Screener [53].

The remaining parent variables, including *single parenthood* and *socio-economic status,* are commonly assessed with demographic questionnaires or items.

Note that *Parenting, Parental Affect, Parental Stress* and *Parenting Self-efficacy* will serve as baseline moderators (to predict parenting outcomes), mediators (in the relationship between BPT and child outcomes) and outcome variables (in the moderator analysis). *Parental Depression, Parental ADHD, single parenthood and socio-economic status* will serve as baseline moderators to predict parenting outcomes.

Additionally the following child variables will be selected:

*Child ADHD severity* will be assessed using a parent-rated measure of childhood ADHD, such as the ADHD subscale of the Connors Parent Rating Scale [54].



For peer review only - http://bmjopen.bmj.com/site/about/guidelines.xhtml

*Comorbidity* will be assessed using symptoms of Oppositional Defiant Disorder (ODD) and/or Conduct Disorder (CD), by for example the ODD subscale of the Connors Parent Rating Scale [54], or the CD subscale of the Disruptive Behavior Disorder Rating Scale [55]. In addition, internalizing symptoms will be assessed, using for example the internalizing subscale of the Child Behavior Checklist [56].

*Global Impairment* will be assessed using a parent- or clinician-rated measure of global impairment, such as the Impairment Rating Scale [57].

The remaining child variables, including *child age, medication use and IQ* are commonly assessed with demographic questionnaires or items.

*Child ADHD severity, comorbidity and global impairment* will serve as baseline moderators (to predict parenting outcomes) and outcome variables (in the mediation analysis). *Child age, medication use and IQ* will solely serve as baseline moderators to predict parenting outcomes.

For all variables (moderators and outcomes), when a study reports multiple measures to capture the same concept, the measure which is most often used by other studies will be included. Regarding harmonization, for each dataset continuous measures will be converted into z-scores, using pre-intervention-score standard deviations within studies.

Quality assessment

Quality assessment will be done independently by three authors (a combination of AG, RH and LAS) using Cochrane risk of bias. Any disagreement will be resolved by consensus. Once the data has been received, all raw data sets will be checked for impossible, missing, or extreme values. We will collect data on all randomized participants, and subsequently adopt the intention to treat principle. As this will possibly reintroduce participants who were previously excluded, we will also check randomization parameters (e.g., age, gender, and ADHD severity of the participants). If any unexpected deviations are found between our results and the published results, the original researcher will be contacted to locate the origin of this deviation.

Analysis

Effects of BPT will be calculated using a one-stage IPDMA, in which data from participants across studies will be analyzed in one stage, clustered by study. A linear multilevel analysis will be used to examine the effects of BPT on parenting outcome measures. A random intercept for study will be added to each model. Post intervention outcome measures will be used as dependent variables in these models, and pre-intervention outcome measures and intervention group will be added. The interaction between intervention group and moderators of interest will be added to the model to assess their moderating effect on treatment outcomes. To analyze changes in parenting as



For peer review only - http://bmjopen.bmj.com/site/about/guidelines.xhtml

a potential mediator in the relationship between BPT and child outcomes, the change in parenting (post-treatment score – baseline score) will be used. An interaction between intervention group and change in parenting will be added to the model to determine whether change in parenting had a main effect on child outcomes and/or an interactive effective with treatment [58,59]. We will conduct sensitivity analyses between studies that provided data and those that did not, for demographic characteristics (e.g., age) and reported effect sizes.

**Discussion**

The current protocol presents the first IPDMA to synthesize research findings on treatment effects of BPT for children with ADHD on parenting outcomes. Both child and parent moderators of parenting outcomes will be explored and a mediation analysis will be conducted to examine whether changes in parenting mediate the effect of BPT on child behaviors and impairment. Previous efforts to identify moderators of parenting outcomes are scarce, as there are few well powered RCTs and only two meta-analyses on this topic [3,6]. The heterogeneity found in parenting outcomes in previous meta-analyses suggest that there is not a “one size fits all” solution of BPT for children with ADHD. Parents and children who differ with regard to their personal, clinical and demographic characteristics, are also likely to differ in their response to BPT. Clearly, an IPDMA approach is needed to further examine the effects of BPT on parenting outcomes, to elucidate potential sources of heterogeneity amongst children and parents, and to investigate potential mechanisms of change in BPT for children with ADHD. A previous IPDMA of our group [5] has already shown that behavioral interventions indeed have worse outcomes for the child, when the presence and severity of comorbid (symptoms of) conduct disorder is considered, in addition to the severity of ADHD symptoms and/or single-parent status. Ultimately, both research and clinical practice may be informed by the knowledge of which child and/or parent responds best to a certain treatment, thereby contributing to the overall goal of providing the best care to children with ADHD and their parents.

An additional important aim of the current IPDMA will be to examine a crucial assumption about the working mechanism of BPT, namely whether improvements in parenting mediate improvements in child behavior and impairment. So far, single studies have examined this assumption, finding some supportive evidence [12,19], but this hypothesis has never been addressed in a meta-analysis, let alone by using an IPDMA approach. By synthesizing all available (raw) data from RCTs, we will conduct highly sophisticated and powered statistical analyses, to provide insight into the working mechanisms of BPT. This knowledge will allow us to further improve and refine BPT programs for children with ADHD.



For peer review only - http://bmjopen.bmj.com/site/about/guidelines.xhtml

Despite clear strengths of the IPDMA approach, such as high-powered moderator, mediation and subgroup analyses, there are some limitations as well [24,26,60]. First, IPDMA cannot change anything about the way the study was originally conducted. Second, it is unclear whether enough studies measured the variables of interest, and thus whether all intended outcomes can be examined. Third, if investigators of trials are unwilling to share their data (or may not be able to share their data), not all relevant data can be included in the synthesis.

Notwithstanding these limitations, this study has the potential to elucidate clinically relevant questions concerning the efficacy of BPT for children with ADHD and their parents and to provide insight in moderators and mediators of treatment effects. This knowledge may improve and optimize current treatment programs and could eventually lead to advances in personalized treatment for children with ADHD.

## References


1 Wolraich M, Brown L, Brown RT, *et al.* ADHD: Clinical practice guideline for the diagnosis, evaluation, and treatment of attention-deficit/ hyperactivity disorder in children and adolescents. Pediatrics. 2011. doi:10.1542/peds.2011-2654

2 NICE NI for H and CE. Attention deficit hyperactivity disorder: diagnosis and management (NICE Guideline 87). Natl. Inst. Heal. Care Excell. 2018.

3 Daley D, Van Der Oord S, Ferrin M, *et al.* Behavioral interventions in attention-deficit/hyperactivity disorder: A meta-analysis of randomized controlled trials across multiple outcome domains. J. Am. Acad. Child Adolesc. Psychiatry. 2014. doi:10.1016/j.jaac.2014.05.013

4 Kazdin AE. Parent management training: Evidence, outcomes, and issues. *J Am Acad Child Adolesc Psychiatry* Published Online First: 1997. doi:10.1097/00004583-199710000-00016

5 Groenman AP, Hornstra R, Hoekstra PJ, *et al.* Behavioral treatments for children and adolescents with attention-deficit/hyperactivity disorder: an individual participant data meta-analysis. *Submitted*

6 Rimestad ML, Lambek R, Zacher Christiansen H, *et al.* Short- and Long-Term Effects of Parent Training for Preschool Children With or at Risk of ADHD: A Systematic Review and Meta-Analysis. J. Atten. Disord. 2019. doi:10.1177/1087054716648775

7 Sonuga-Barke EJ, Brandeis D, Cortese S, *et al.* Nonpharmacological interventions for ADHD: Systematic review and meta-analyses of randomized controlled trials of dietary and psychological treatments. Am. J. Psychiatry. 2013. doi:10.1176/appi.ajp.2012.12070991

8 Mulqueen JM, Bartley CA, Bloch MH. Meta-Analysis: Parental Interventions for Preschool




For peer review only - http://bmjopen.bmj.com/site/about/guidelines.xhtml

ADHD. *J Atten Disord* Published Online First: 2015. doi:10.1177/1087054713504135

9 Daley D, Van Der Oord S, Ferrin M, *et al.* Practitioner review: Current best practice in the use of parent training and other behavioural interventions in the treatment of children and adolescents with attention deficit hyperactivity disorder. *J Child Psychol Psychiatry Allied Discip* Published Online First: 2017. doi:10.1111/jcpp.12825

10 Frick P. The Alabama Parenting Questionnaire. *Unpubl Rat scale, Univ Alabama* 1991.

11 Morris AS, Silk JS, Steinberg L, *et al.* Temperamental vulnerability and negative parenting as interacting predictors of child adjustment. J. Marriage Fam. 2002. doi:10.1111/j.1741-3737.2002.00461.x

12 Hinshaw SP, Owens EB, Wells KC, *et al.* Family processes and treatment outcome in the MTA: Negative/ineffective parenting practices in relation to multimodal treatment. *J Abnorm Child Psychol* 2000;**28**:555–68. doi:10.1023/A:1005183115230

13 Sanders MR. Triple P-Positive Parenting Program as a Public Health Approach to Strengthening Parenting. *J Fam Psychol* Published Online First: 2008. doi:10.1037/0893-3200.22.3.506

14 Wells KC, Epstein JN, Hinshaw SP, *et al.* Parenting and Family stress treatment outcomes in attention deficit hyperactivity disorder. *J Abnorm Child Psychol* 2000;**6**:543–53.https://link.springer.com/content/pdf/10.1023%2FA%3A1005131131159.pdf

15 Daley D, Sonuga-Barke EJS, Thompson M. Assessing expressed emotion in mothers of preschool AD/HD children: Psychometric properties of a modified speech sample. *Br J Clin Psychol* Published Online First: 2003. doi:10.1348/014466503762842011

16 Herbert SD, Harvey EA, Roberts JL, *et al.* A Randomized Controlled Trial of a Parent Training and Emotion Socialization Program for Families of Hyperactive Preschool-Aged Children. *Behav Ther* 2013;**44**:302–16. doi:10.1016/j.beth.2012.10.004

17 Deater-Deckard K, Smith J, Ivy L, *et al.* Differential perceptions of and feelings about sibling children: Implications for research on parenting stress. Infant Child Dev. 2005. doi:10.1002/icd.389

18 Johnson JH, Reader SK. Assessing stress in families of children with ADHD: Preliminary development of the disruptive behavior stress inventory (DBSI). *J Clin Psychol Med Settings* Published Online First: 2002. doi:10.1023/A:1014136029697

19 Heath CL, Curtis DF, Fan W, *et al.* The Association Between Parenting Stress, Parenting Self-Efficacy, and the Clinical Significance of Child ADHD Symptom Change Following Behavior Therapy. *Child Psychiatry Hum Dev* 2014;**46**:118–29. doi:10.1007/s10578-014-0458-2

20 Gershy N, Meehan KB, Omer H, *et al.* Randomized Clinical Trial of Mindfulness Skills Augmentation in Parent Training. *Child Youth Care Forum* 2017;**46**:783–803.



For peer review only - http://bmjopen.bmj.com/site/about/guidelines.xhtml

doi:10.1007/s10566-017-9411-4

21 Van Den Hoofdakker BJ, Nauta MH, Veen-Mulders L Van Der, *et al.* Behavioral parent training as an adjunct to routine care in children with attention-deficit/hyperactivity disorder: Moderators of treatment response. *J Pediatr Psychol* Published Online First: 2010. doi:10.1093/jpepsy/jsp060

22 Veenman B, Luman M, Oosterlaan J. Moderators influencing the effectiveness of a behavioral teacher program. *Front Psychol* Published Online First: 2018. doi:10.3389/fpsyg.2018.00298

23 Owens EB, Hinshaw SP, Arnold LE, *et al.* Which treatment for whom for ADHD? Moderators of treatment response in the MTA. J. Consult. Clin. Psychol. 2003. doi:10.1037/0022-006X.71.3.540

24 Riley RD, Lambert PC, Abo-Zaid G. Meta-analysis of individual participant data: Rationale, conduct, and reporting. *BMJ* Published Online First: 2010. doi:10.1136/bmj.c221

25 Higgins JPT, Jackson D, Barrett JK, *et al.* Consistency and inconsistency in network meta-analysis: concepts and models for multi-arm studies. *Res Synth Methods* 2012;**3**:98–110. doi:10.1002/jrsm.1044

26 Stewart LA, Tierney JF. To IPD or not to IPD? Advantages and disadvantages of systematic reviews using individual patient data. Eval. Heal. Prof. 2002. doi:10.1177/016327870202500106

27 Owens EB, Hinshaw SP, McBurnett K, *et al.* Predictors of Response to Behavioral Treatments Among Children With ADHD-Inattentive Type. *J Clin Child Adolesc Psychol* Published Online First: 2018. doi:10.1080/15374416.2016.1228461

28 Jensen, Arnold, Richters, Severe, Vereen V al. (1999). Moderators and mediators of treatment response for children with attention-deficit/hyperactivity disorder: The multimodal treatment study of children with attention-deficit/hyperactivity disorder. *Arch Gen Psychiatry* Published Online First: 1999. doi:10.1001/archpsyc.56.12.1088

29 Hallahan DP. Cognitive-behavioral therapy for impulsive children. *Child Youth Serv Rev* Published Online First: 1986. doi:10.1016/0190-7409(86)90045-9

30 Kazdin AE, Crowley MJ. Moderators of treatment outcome in cognitively based treatment of antisocial children. *Cognit Ther Res* Published Online First: 1997. doi:10.1023/A:1021832729480

31 Van der Oord S, Prins PJM, Oosterlaan J, *et al.* Efficacy of methylphenidate, psychosocial treatments and their combination in school-aged children with ADHD: A meta-analysis. Clin. Psychol. Rev. 2008. doi:10.1016/j.cpr.2007.10.007

32 Lee SS, Falk AE, Aguirre VP. Association of comorbid anxiety with social functioning in school-age children with and without attention-deficit/hyperactivity disorder (ADHD). *Psychiatry Res*

For peer review only



For peer review only - http://bmjopen.bmj.com/site/about/guidelines.xhtml

Published Online First: 2012. doi:10.1016/j.psychres.2012.01.018

33 Langberg JM, Evans SW, Schultz BK, *et al.* Trajectories and Predictors of Response to the Challenging Horizons Program for Adolescents With ADHD. *Behav Ther* Published Online First: 2016. doi:10.1016/j.beth.2016.01.001

34 Chronis-Tuscano A, O'Brien KA, Johnston C, *et al.* The relation between maternal ADHD symptoms & improvement in child behavior following brief behavioral parent training is mediated by change in negative parenting. *J Abnorm Child Psychol* 2011;**39**:1047–57. doi:10.1007/s10802-011-9518-2

35 Sonuga-Barke EJS, Daley D, Thompson M. Does Maternal ADHD Reduce the Effectiveness of Parent Training for Preschool Children's ADHD? *J Am Acad Child Adolesc Psychiatry* Published Online First: 2002. doi:10.1097/00004583-200206000-00009

36 Farmer CA, Brown N V., Gadow KD, *et al.* Comorbid Symptomatology Moderates Response to Risperidone, Stimulant, and Parent Training in Children with Severe Aggression, Disruptive Behavior Disorder, and Attention-Deficit/Hyperactivity Disorder. *J Child Adolesc Psychopharmacol* Published Online First: 2015. doi:10.1089/cap.2014.0109

37 Dumas JE, Wahler RG. Predictors of treatment outcome in parent training: Mother insularity and socioeconomic disadvantage. *Behav Assess* 1983.

38 Webster-Stratton C, Hammond M. Predictors of treatment outcome in parent training for families with conduct problem children. *Behav Ther* Published Online First: 1990. doi:10.1016/S0005-7894(05)80334-X

39 Webster-Stratton C. Predictors of treatment outcome in parent training for conduct disordered children. *Behav Ther* Published Online First: 1985. doi:10.1016/S0005-7894(85)80048-4

40 van den Hoofdakker BJ, Hoekstra PJ, van der Veen-Mulders L, *et al.* Paternal influences on treatment outcome of behavioral parent training in children with attention-deficit/hyperactivity disorder. *Eur Child Adolesc Psychiatry* 2014;**23**:1071–9. doi:10.1007/s00787-014-0557-4

41 Booster GD, Mautone JA, Nissley-Tsiopinis J, *et al.* Reductions in negative parenting practices mediate the effect of a family-school intervention for children with attention deficit hyperactivity disorder. *School Psych Rev* Published Online First: 2016. doi:10.17105/SPR45-2.192-208

42 Shamseer L, Moher D, Clarke M, *et al.* Preferred reporting items for systematic review and meta-analysis protocols (prisma-p) 2015: Elaboration and explanation. BMJ. 2015. doi:10.1136/bmj.g7647

43 Evans SW, Owens JS, Bunford N. Evidence-Based Psychosocial Treatments for Children and



For peer review only - http://bmjopen.bmj.com/site/about/guidelines.xhtml

Adolescents with Attention-Deficit/Hyperactivity Disorder. *J Clin Child Adolesc Psychol* Published Online First: 2014. doi:10.1080/15374416.2013.850700

44 Eyberg SM, Nelson MMD, Duke M, *et al.* Manual for the Dyadic Parent-Child Interaction Coding System Third Edition. *System* 2004.

45 Rubinic D, Schwickrath H. Test Review: Kamphaus, R. W., & Reynolds, C. R. (2006). Parenting Relationship Questionnaire. Minneapolis, MN: NCS Pearson. J. Psychoeduc. Assess. 2010. doi:10.1177/0734282909346718

46 Abidin RR. Parenting Stress and the Utilization of Pediatric Services. *Child Heal Care* 1982;**11**:70–3. doi:10.1207/s15326888chc1102_5

47 Clara IP, Cox BJ, Enns MW. Confirmatory Factor Analysis of the Depression-Anxiety-Stress Scales in Depressed and Anxious Patients. *J Psychopathol Behav Assess* 2001;**23**:61–7. doi:10.1023/A:1011095624717

48 Ohan JL, Leung DW, Johnston C. The parenting sense of competence scale: Evidence of a stable factor structure and validity. *Can J Behav Sci* Published Online First: 2000. doi:10.1037/h0087122

49 Mautone JA, Marcelle E, Tresco KE, *et al.* Assessing the quality of parent-teacher relationships for students with ADHD. *Psychol Sch* Published Online First: 2015. doi:10.1002/pits.21817

50 Beck AT, Steer RA, Carbin MG. Psychometric properties of the Beck Depression Inventory: Twenty-five years of evaluation. *Clin Psychol Rev* Published Online First: 1988. doi:10.1016/0272-7358(88)90050-5

51 Henry JD, Crawford JR. The short-form version of the Depression Anxiety Stress Scales (DASS-21): construct validity and normative data in a large non-clinical sample. *Br J Clin Psychol* 2005;**44**:227–39. doi:10.1348/014466505X29657

52 Goldberg DP, Hillier VF. A scaled version of the General Health Questionnaire. *Psychol Med* Published Online First: 1979. doi:10.1017/S0033291700021644

53 Kessler RC, Adler L, Ames M, *et al.* The World Health Organization adult ADHD self-report scale (ASRS): A short screening scale for use in the general population. *Psychol Med* Published Online First: 2005. doi:10.1017/S0033291704002892

54 Kao GS, Thomas HM. Test Review: C. Keith Conners Conners 3rd Edition Toronto, Ontario, Canada: Multi-Health Systems, 2008. *J Psychoeduc Assess* Published Online First: 2010. doi:10.1177/0734282909360011

55 Pelham Jr. WE, Gnagy EM, Greenslade KE, *et al.* Teacher ratings of DSM-III-R symptoms for the disruptive behavior disorders.[erratum appears in J Am Acad Child Adolesc Psychiatry 1992 Nov;31(6):1177]. *J Am Acad Child Adolesc Psychiatry* 1992.

56 Achenbach TM. *Manual for the Youth Self Report and 1991 Profile*. 1991.

For peer review only

57 Fabiano GA, Pelham WE, Waschbusch DA, *et al.* A practical measure of impairment: Psychometric properties of the impairment rating scale in samples of children with attention deficit hyperactivity disorder and two school-based samples. *J Clin Child Adolesc Psychol* Published Online First: 2006. doi:10.1207/s15374424jccp3503_3

58 HC K, GT W, CG F, *et al.* Mediators and moderators of treatment effects in randomized clinical trials. *Arch Gen Psychiatry* 2002.

59 Haasova M, Warren FC, Ussher M, *et al.* The acute effects of physical activity on cigarette cravings: Exploration of potential moderators, mediators and physical activity attributes using individual participant data (IPD) meta-analyses. Psychopharmacology (Berl). 2014. doi:10.1007/s00213-014-3450-4

60 Higgins JPT, Whitehead A, Turner RM, *et al.* Meta-analysis of continuous outcome data from individual patients. *Stat Med* Published Online First: 2001. doi:10.1002/sim.918

For peer review only



For peer review only - http://bmjopen.bmj.com/site/about/guidelines.xhtml

Supplement 1- Search terms per database

**Behavioral interventions**

**PubMed**
("Attention Deficit Disorder with Hyperactivity"[Mesh] OR ADHD[tiab] OR ADD[tiab] OR attention deficit*[tiab] OR hyperactiv*[tiab] OR hyperkinetic*[tiab] OR minimal brain deficit*[tiab] OR minimal brain dysfunction*[tiab])
**AND**

(("Psychotherapy"[Mesh] OR psychotherap*[tiab] OR psychological therap*[tiab] OR psychological intervent*[tiab] OR psychoeduca*[tiab] OR mentoring*[tiab] OR coaching*[tiab] OR mindful*[tiab] OR relax*[tiab] OR meditat*[tiab])
OR
((parent*[tiab] OR mother[tiab] OR father[tiab] OR teacher*[tiab] OR school*[tiab])
*AND*
(program*[tiab] OR train*[tiab] OR educa*[tiab] OR therapy*[tiab] OR therapies*[tiab] OR therapeu*[tiab]OR intervention*[tiab] OR coaching*[tiab] OR counseling*[tiab]))
OR
((behavio*[tiab] OR cognit*[tiab] OR “acceptance and commitment”[tiab] OR dialectica*[tiab])
*AND*
(program*[tiab] OR therapy*[tiab] OR therapies*[tiab] OR therapeu*[tiab] OR intervention*[tiab] OR treatment*[tiab] OR train*[tiab]))
OR
(psychosocial[tiab]
AND
(treatment*[tiab] OR therapy*[tiab] OR therapies*[tiab] OR therapeu*[tiab] OR train*[tiab] OR intervention*[tiab] OR program*[tiab]))
OR
((skill*[tiab] OR organization*[tiab] OR organisation*[tiab] OR planning*[tiab] OR play*[tiab])
AND
(train*[tiab] OR intervention*[tiab] OR program*[tiab])))

**AND**

("Adolescent"[Mesh] OR "Child"[Mesh] OR child*[tiab] OR school*[tiab] OR infan*[tiab] OR adolescen*[tiab] OR pediatri*[tiab] OR paediatr*[tiab] OR boy[tiab] OR boys[tiab] OR boyhood[tiab] OR girl[tiab] OR girls[tiab] OR girlhood[tiab] OR youth[tiab] OR youths[tiab] OR teen[tiab] OR teens[tiab] OR teenage*[tiab] OR puberty[tiab] OR preschool*[tiab] OR toddler*[tiab] OR juvenile*[tiab] OR kids[tiab])
**AND**

("Controlled Clinical Trial" [Publication Type] OR "Randomized Controlled Trials as Topic"[Mesh] OR "Follow-Up Studies"[Mesh] OR follow-up[tiab] OR followup[tiab] OR control group*[tiab] OR "Random Allocation"[Mesh] OR random*[tiab] OR trial[ti])

For peer review only

**EMBASE EMBASE.com** (losse delen combineren op website)
('attention deficit disorder'/exp OR (ADHD OR ADD OR 'attention deficit*'
OR hyperactiv* OR hyperkinetic* OR 'minimal brain deficit*' OR 'minimal brain dysfunction*'):ab,ti)
**AND**

('psychotherapy'/exp OR (psychotherap* OR 'psychological therap*' OR 'psychological intervent*'
OR psychoeduca* OR mentoring* OR coaching* OR mindful* OR relax*):ab,ti)
OR
((((parent* OR mother OR father OR teacher* OR school*)
*AND*
(program* OR train* OR educa* OR therapy* OR therapies* OR therapeu*OR intervention*
OR coaching* OR counseling*))
OR
((behavio* OR cognit* OR 'acceptance and commitment' OR dialectica*)
*AND*
(program* OR therapy* OR therapies* OR therapeu* OR intervention* OR treatment* OR
train*))
OR
(psychosocial
AND
(treatment* OR therapy* OR therapies* OR therapeu* OR train* OR intervention* OR
program*))
OR
((skill* OR organization* OR organisation* OR planning* OR play*)
AND
(train* OR intervention* OR program*))):ab,ti)

**AND**
('juvenile'/exp OR (child* OR school* OR infan* OR adolescen* OR pediatri* OR paediatr* OR boy OR
boys OR boyhood OR girl OR girls OR girlhood OR youth OR youths OR teen OR teens OR teenage*
OR puberty OR preschool* OR toddler* OR juvenile* OR kids):ab,ti)

**AND**
('controlled clinical trial'/exp OR 'randomization'/exp OR ('control group*' OR random*):ab,ti OR
trial:ti)

For peer review only

**EBSCO PsycINFO**
(DE "Attention Deficit Disorder" OR DE "Attention Deficit Disorder with Hyperactivity" OR ADHD OR ADD OR “attention deficit*” OR hyperactiv* OR hyperkinetic* OR “minimal brain deficit*” OR “minimal brain dysfunction*”)
**AND**
(DE "Psychotherapy" OR DE "Child Psychotherapy" OR DE "Play Therapy" OR DE "Behavior Therapy" OR DE "Aversion Therapy" OR DE "Conversion Therapy" OR DE "Dialectical Behavior Therapy" OR DE "Exposure Therapy" OR DE "Implosive Therapy" OR DE "Reciprocal Inhibition Therapy" OR DE "Response Cost" OR DE "Systematic Desensitization Therapy" AND DE "Cognitive Behavior Therapy" OR DE "Cognitive Therapy" OR DE "Group Psychotherapy" OR DE "Neurotherapy" OR psychotherap* OR “psychological therap*” OR “psychological intervent*” OR psychoeduca* OR mentoring* OR coaching* OR mindful* OR relax*)

OR

((parent* OR mother OR father OR teacher* OR school*)

*AND*

(program* OR train* OR educa* OR therapy* OR therapies* OR therapeu*OR intervention* OR coaching* OR counseling*))

OR

((behavio* OR cognit* OR ‘acceptance and commitment’ OR dialectica*)

*AND*

(program* OR therapy* OR therapies* OR therapeu* OR intervention* OR treatment* OR train*))

OR

(psychosocial

AND

(treatment* OR therapy* OR therapies* OR therapeu* OR train* OR intervention* OR program*))

OR

((skill* OR organization* OR organisation* OR planning* OR play*)

AND

(train* OR intervention* OR program*))

**AND**
(AG (childhood OR adolescence) OR (child* OR school* OR infan* OR adolescen* OR pediatri* OR paediatr* OR boy OR boys OR boyhood OR girl OR girls OR girlhood OR youth OR youths OR teen OR teens OR teenage* OR puberty OR preschool* OR toddler* OR juvenile* OR kids))
**AND**
(“control group*” OR random* OR "controlled trial" OR "controlled study" OR "experimental study" OR "experimental design" OR TI trial)

**EBSCO CINAHL**
(MH "Attention Deficit Hyperactivity Disorder" OR "Attention Deficit Disorder with Hyperactivity" OR ADHD OR ADD OR “attention deficit*” OR hyperactiv* OR hyperkinetic* OR “minimal brain deficit*” OR “minimal brain dysfunction*”)
**AND**
(MH "Psychotherapy+" OR psychotherap* OR “psychological therap*” OR “psychological intervent*” OR psychoeduca* OR mentoring* OR coaching* OR mindful* OR relax*)

OR

((parent* OR mother OR father OR teacher* OR school*)

*AND*

For peer review only

(program* OR train* OR educa* OR therapy* OR therapies* OR therapeu*OR intervention* OR coaching* OR counseling*))
OR
((behavio* OR cognit* OR ‘acceptance and commitment’ OR dialectica*)
*AND*
(program* OR therapy* OR therapies* OR therapeu* OR intervention* OR treatment* OR train*))
OR
(psychosocial
AND
(treatment* OR therapy* OR therapies* OR therapeu* OR train* OR intervention* OR program*))
OR
((skill* OR organization* OR organisation* OR planning* OR play*)
AND
(train* OR intervention* OR program*))
**AND**
(AG child OR (child* OR school* OR infan* OR adolescen* OR pediatri* OR paediatr* OR boy OR boys OR boyhood OR girl OR girls OR girlhood OR youth OR youths OR teen OR teens OR teenage* OR puberty OR preschool* OR toddler* OR juvenile* OR kids))
**AND**
(MH "Clinical Trials+" OR “control group*” OR random* OR "controlled trial" OR "controlled study" OR "experimental study" OR "experimental design" OR TI trial)

**EBSCO ERIC**
(DE "Attention Deficit Disorder" OR DE "Attention Deficit Hyperactivity Disorder" OR ADHD OR ADD OR “attention deficit*” OR hyperactiv* OR hyperkinetic* OR “minimal brain deficit*” OR “minimal brain dysfunction*”)
**AND**

(DE "Psychotherapy" OR psychotherap* OR “psychological therap*” OR “psychological intervent*” OR psychoeduca* OR mentoring* OR coaching* OR mindful* OR relax*)
OR
((parent* OR mother OR father OR teacher* OR school*)
*AND*
(program* OR train* OR educa* OR therapy* OR therapies* OR therapeu*OR intervention* OR coaching* OR counseling*))
OR
((behavio* OR cognit* OR ‘acceptance and commitment’ OR dialectica*)
*AND*
(program* OR therapy* OR therapies* OR therapeu* OR intervention* OR treatment* OR train*))
OR
(psychosocial
AND
(treatment* OR therapy* OR therapies* OR therapeu* OR train* OR intervention* OR program*))
OR
((skill* OR organization* OR organisation* OR planning* OR play*)
AND

For peer review only

(train* OR intervention* OR program*))

**AND**

(child* OR school* OR infan* OR adolescen* OR pediatri* OR paediatr* OR boy OR boys OR boyhood OR girl OR girls OR girlhood OR youth OR youths OR teen OR teens OR teenager* OR puberty OR preschool* OR toddler* OR juvenile* OR kids))

**AND**

(“control group*” OR random* OR "controlled trial" OR "controlled study" OR "experimental study" OR "experimental design" OR TI trial)

**Web of Science**

(TS=("Attention Deficit Disorder with Hyperactivity" OR ADHD OR ADD OR “attention deficit*” OR hyperactiv* OR hyperkinetic* OR “minimal brain deficit*” OR “minimal brain dysfunction*”))

**AND**

(TS=(psychotherap* OR psychotherap* OR “psychological therap*” OR “psychological intervent*” OR psychoeduca* OR mentoring* OR coaching* OR mindful* OR relax*

OR

((parent* OR mother OR father OR teacher* OR school*)

*AND*

(program* OR train* OR educa* OR therapy* OR therapies* OR therapeu*OR intervention* OR coaching* OR counseling*))

OR

((behavio* OR cognit* OR ‘acceptance and commitment’ OR dialectica*)

*AND*

(program* OR therapy* OR therapies* OR therapeu* OR intervention* OR treatment* OR train*))

OR

(psychosocial

AND

(treatment* OR therapy* OR therapies* OR therapeu* OR train* OR intervention* OR program*))

OR

((skill* OR organization* OR organisation* OR planning* OR play*)

AND

(train* OR intervention* OR program*))))

**AND**

(TS=(child* OR school* OR infan* OR adolescen* OR pediatri* OR paediatr* OR boy OR boys OR boyhood OR girl OR girls OR girlhood OR youth OR youths OR teen OR teens OR teenager* OR puberty OR preschool* OR toddler* OR juvenile* OR kids))

**AND**

(TS=(“clinical trial*” OR “control group*” OR random* OR "controlled trial" OR "controlled study" OR "experimental study" OR "experimental design"))

For peer review only

**Cochrane Central register of Controlled Trials** *(CENTRAL)*

("Attention Deficit Disorder with Hyperactivity" OR ADHD OR ADD OR “attention deficit*” OR hyperactiv* OR hyperkinetic* OR “minimal brain deficit*” OR “minimal brain dysfunction*”)

**AND**

((psychotherap* OR “psychological therap*” OR “psychological intervent*” OR psychoeduca* OR mentoring* OR coaching* OR mindful* OR relax*)

OR

((parent* OR mother OR father OR teacher* OR school*)

*AND*

(program* OR train* OR educa* OR therapy* OR therapies* OR therapeu*OR intervention* OR coaching* OR counseling*))

OR

((behavio* OR cognit* OR ‘acceptance and commitment’ OR dialectica*)

*AND*

(program* OR therapy* OR therapies* OR therapeu* OR intervention* OR treatment* OR train*))

OR

((psychosocial)

AND

(treatment* OR therapy* OR therapies* OR therapeu* OR train* OR intervention* OR program*))

OR

((skill* OR organization* OR organisation* OR planning* OR play*)

AND

(train* OR intervention* OR program*)))

**AND**

(child* OR school* OR infan* OR adolescen* OR pediatri* OR paediatr* OR boy OR boys OR boyhood OR girl OR girls OR girlhood OR youth OR youths OR teen OR teens OR teenage* OR puberty OR preschool* OR toddler* OR juvenile* OR kids)

For peer review only

Supplement 2. Data sharing manual

**Data collection form**

**Content**

**Introduction**
**How do I transfer my data?**
**Which participants do we need?**
**Which data do we need?**
**What will we do with the data?**
**Supplement 3A. Measures**

Thank you for taking place in our Psychosocial ADHD INTerverntions (PAINT) collaborators group. We are very grateful that you are willing to put your time into this project. We believe that this group will be fruitful and successful collaboration with the aim to get more insight into psychosocial interventions for ADHD.
With this manual, we hope to provide you with guidance on the data collection. However, if you have any questions regarding the data handling, despite reading this manual, please let us know so we can help you further, and adapt our manual accordingly. You can reach us at paint@accare.nl.

Most of the choices we have made concerning data collection are driven by a need to keep methods and measures similar across trials. Every study is designed slightly different, with different measures, different in- and exclusion criteria, different methods to handle missing data, etc..
To harmonize measures and methods, at some point we will have to make choices regarding data analysis, that differ from the choices made in the original study. For example, we will do intention to treat analysis, while it may be possible that you did not do this in your original analysis. This means that, if available, we will ask for data or participants not included in the original analysis. Furthermore, it is important to mention that we will not report on results from a single trial, although we can report a forestplot displaying all effect sizes of included studies.

How do I transfer my data?
Preferably, we would receive the information electronically. We can work with any kind of data file (STATA, SPSS, etc.), but we prefer a layout, where each line represents a subject.

You can email the data file once you encrypted it using a program such as 7-zip (an explanation on how to do this is included in supplement B). It is important that the password with which you will protect your data file, will be send in a separate email from the email that contains the data file (as attachment). Another important point in transferring the data is that the data should be anonymized. This means that there is no information with which we could trace a single participant back to the individual. This means that no names should be included in the file. However, all other information that can identify a person should be left out of this file. This can mean combinations of variables, for example postal code and date of birth. Please make sure that this kind of information is not present in the data file. If these kinds of examples arise, please contact us, so we can think with you on what best to do.

Which participants do we need?
We want the data of all included participants. This means all participants that have a baseline measure and were randomized. It could be possible that this is a different approach than you took in

For peer review only

your analysis of the data. Because we want to perform intention to treat analysis, we need all data from included participants.

Which data do we need?

We ask you for clean data (please contact us if this provides you with any questions). We want sum scores and if available norm-scores of questionnaires. Proper labeling is important. In the Table below we provide you with an overview of constructs that we are interested in. It could be possible that the measure or instrument used in your study is not mentioned in this Table, this does not mean that we are not interested, the measures mentioned are mere examples. If there are any remarks on this measure (e.g. certain items were not used) please mention these in the column “remarks”. Please indicate which informants filled out the questionnaires, when multiple informants are used please provide us with data from all informants. If multiple instruments have been used to measure a certain construct, we would really like to receive all instruments (for example a self and a proxy version, or multiple ADHD measures). This could aid us in the harmonization process (see above). If a construct is measured with an in-house instrument or if you made any adjustments on the instrument, could you also provide us this instrument?

For questionnaires, we would like to receive the, sum scores and norm-scores and if available item scores. We would always like to receive the full questionnaire if possible, and not just certain subscales. In the future, it could be possible that we will ask you about norming tables, we hope however, that we will not need these.

For (structured) interviews we would like to receive items scores, sum scores and outcomes. We would like to receive information on the full interview, not only certain outcomes.

Concerning cognitive measures, we would like to receive calculated outcomes variables across trials and conditions, not mere trial to trial performance. For example, in the stop task, we would like to receive measures such as SSRT, mean (and SD) of reaction time on Go trials, number of commissions and omissions, but not the individual performance on a single trial of the stop task. We fill also need information about the task such as number of conditions, number of trials, and how the outcome variables were calculated.

For variables that are available on multiple time points, please provide us with all relevant data. For example, if an ADHD measure is taken at baseline, right after the intervention and after 3 months, we would like to receive this measure assessed at all time points. Again, proper labelling is very important. Please label the data in English (or Dutch).

The constructs and variables that we are interested in for the IPD meta-analysis are shown below. We understand that not all studies have included all measures. The table below will provide you with a list you can keep on hand when preparing the data before sending it. Next to the constructs are examples of instruments. Please remember that this is not a complete list, and that it could be possible that you have used an instrument that is not in our list, but that would still be very valuable for this IPD meta-analysis. Please remember that you do not have to recode your data. We will do this for all datasets.

What will we do with the data?

Once we receive the data we will do some basic data checks. These will include checking missing and impossible values. If we will have any questions concerning the data during or after these checks we will contact you for clarification. After that we will harmonize the data with the other datasets. This harmonization process depends on the available data. For example, this could mean that we have to dichotomize data, or making norm deviation scores (translating raw questionnaire scores on one

For peer review only

scale into raw questionnaire scores on another scale using norm scores). Harmonization is easiest if the same measure has been used across studies. If you have used multiple instruments to measure a certain outcome (e.g. more than 1 ADHD scale), we ask you to provide us both measures. This could mean that we may be able use a latent variable for some measures, or we could calculate correlations between measures.

The original dataset will be saved as the original file. Any harmonization will take place in a separate file, which are only later merged into one big data file.

For peer review only

Supplement 2A.

Tabel 1 Measures.

| **Descriptive measures** | |
|---|---|
| Participant ID | |
| Date of randomization | Dd/mm/yyyy |
| Date of treatment start | Dd/mm/yyyy |
| Date post-intervention measurement | Dd/mm/yyyy |
| Date follow up measurement | Dd/mm/yyyy |
| Date baseline measurement | Dd/mm/yyyy |
| Treatment allocation | 0- Control<br>1- Intervention |
| Age | Age in years |
| Gender | 1-Male<br>2-Female |
| IQ | Norm score of WISC |
| Verbal IQ | Norm score of WISC |
| Performance IQ | Norm score of WISC |
| Ethnicity | |
| Socioeconomic status | |
| ADHD severity at baseline | |
| Comorbidities | ODD/CD/Anxiety/Depression/PDD/Tics/ etc. |
| Number of children living with the family | Total number of children in the household |
| Primary care-taker education | |
| Secundary care-taker education | |
| Household income | Combined income of primary caretaker and (if applicable) secundary caretaker |
| Parental psychopathology (diagnosis) | Parental ADHD/ anxiety/depression/ etc |
| Marital status | |
| Education primary caretaker | |
| Education secondary caretaker | |
| Age Father | Age in years |
| Age mother | Age in years |
| Psychoactive Medication use pre- intervention | Yes/no, if yes which medication, mg MPH if applicable, x times per day, for the last month, |

For peer review only

| | |
|---|---|
| | duration |
| Previous care use of the child | Care use in mental health. |
| Medication use during intervention | Yes/no, if yes, mg per day |
| Treatment expectancy | |
| Stable medication use during intervention | Yes/No/NA |
| Other descriptive measures | |
| **Individual child outcomes- behavioral** | |
| **Measured constructs** | **Examples of instruments** |
| **ADHD** | Conners, CBCL, DBD, ACTeRS, ARS, BASC, SNAP, SWAN, Werry Weiss Peters activity scale, Behavioral checklist, PACS, Parent daily report, KSADS, standardized behavioral observations |
| Disruptive disorders/ behavior | Conners, CBCL, DBD, EDBI, SNAP, SSRS, ACTeRS, KSADS, behavior problem checklist, aggression and conduct problems scale, behavioral checklist, PACS, parent daily reports, standardized behavioral observations |
| Social skills | DSAS, Behavior problem checklist, BASC, ACTeRS, SSRS, CBCL |
| Internalizing problems | Conners, CBCL, SSRS, BASC, Behavior problem checklist, MASC, PACS, KSADS, SCARED, CDI |
| Attachment | |
| Quality of life | HONOSCA, kidsscreen, EQ-5d, IRS |
| Selfconcept | Piers-Harris |
| Which other child behavioral outcomes are available? | |
| **Individual child outcomes- academic outcomes** | |
| Measured Construct | Examples of measures |
| Grades on core courses | Math, Spelling, Reading |
| Standardized Academic Achievement tests | Reading, spelling, math scores, woodcock johnson, WRAT, WISC Durrel |
| On and off task behaviors | Classroom observations, SOAPs |
| Academic competence | Academic rating scale, classroom performance survey |
| Which other academic outcomes are available? | |

For peer review only

| | |
|---|---|
| **Individual child outcomes- cognitive outcomes[a]** | |
| Higher cognitive functions | Working memory, executive functions, inhibition, planning, set shifting, reward sensitivity, emotion regulation |
| Intellectual functioning | IQ (WISC, RAVEN) |
| Motor skills | Fine motor skills, precision, gross motor skills |
| Memory | Visuospatial, verbal |
| Basic cognitive functions | Attention, reaction time, speed, perception |
| **Child individual outcomes- other measures** | |
| Adverse events | Types of adverse events reported |
| Treatment fidelity | |
| Treatment satisfaction | |
| Number of sessions attended | |
| Drop out | Yes/no |
| Date of discontinuation of the intervention | |
| Reason for discontinuation | |
| **Parental outcome measures** | |
| Measured constructs | Examples of measures |
| Parental psychopathology | DASS, GHQ, BDI, PSI, CSS, AARS, SCL90, |
| Parent child interaction, parenting behaviors, parenting style, parenting affect | DPICS or other observational measure, parenting practices inventory (PPI), parent interaction style, PS (parenting scale), measures parental sensitivity/reponsivity |
| Parental competence/ self efficacy | PSI-subscale parental competence, PSOC |
| Spouse relationship | locke wallace marital adjustment, PSI-subscale |
| Parental self esteem | Rosenberg self esteem scale (RSE) |
| Parenting stress | PSI |
| Which other parenting outcomes are available? | |

[a] concerning cognitive measures, we would like to receive calculated outcomes measures, not mere trial to trial performance. For example, in the stop task, we would like to receive measures such as SSRT, mean reaction time, number of commissions and omissions, but not the individual performance on a single trial of the stop task. We will also need information on the conditions of the task, how the outcome was calculated, etc.

For peer review only

PRISMA-P (preferred reporting items for systematic review and meta-analysis protocols) 2015 checklist: recommended items to address in a systematic review protocol

| Section and topic | Item No | Checklist item | Location in manuscript |
|---|---|---|---|
| **Administrative information** | | | |
| Title: | | | |
| Identification | 1a | Identify the report as a protocol of a systematic review | Page 1 |
| Update | 1b | If the protocol is for an update of a previous systematic review, identify as such | NA |
| Registration | 2 | If registered, provide the name of the registry (such as PROSPERO) and registration number | Page 8 |
| Authors: | | | |
| Contact | 3a | Provide name, institutional affiliation, e-mail address of all protocol authors; provide physical mailing address of corresponding author | Page 1 |
| Contributions | 3b | Describe contributions of protocol authors and identify the guarantor of the review | Page 2 |
| Amendments | 4 | If the protocol represents an amendment of a previously completed or published protocol, identify as such and list changes; otherwise, state plan for documenting important protocol amendments | NA |
| Support: | | | |
| Sources | 5a | Indicate sources of financial or other support for the review | Page 2 |
| Sponsor | 5b | Provide name for the review funder and/or sponsor | Page 2 |
| Role of sponsor or funder | 5c | Describe roles of funder(s), sponsor(s), and/or institution(s), if any, in developing the protocol | Page 2 |
| **Introduction** | | | |

| Section and topic | Item No | Checklist item | Location in manuscript |
|---|---|---|---|
| Rationale | 6 | Describe the rationale for the review in the context of what is already known | Page 5-8 |
| Objectives | 7 | Provide an explicit statement of the question(s) the review will address with reference to participants, interventions, comparators, and outcomes (PICO) | Page 8 |
| **Methods** | | | |
| Eligibility criteria | 8 | Specify the study characteristics (such as PICO, study design, setting, time frame) and report characteristics (such as years considered, language, publication status) to be used as criteria for eligibility for the review | Page 9 |
| Information sources | 9 | Describe all intended information sources (such as electronic databases, contact with study authors, trial registers or other grey literature sources) with planned dates of coverage | Page 9 |
| Search strategy | 10 | Present draft of search strategy to be used for at least one electronic database, including planned limits, such that it could be repeated | Page 9 (and supplement) |
| Study records: | | | |
| Data management | 11a | Describe the mechanism(s) that will be used to manage records and data throughout the review | Page 10 |
| Selection process | 11b | State the process that will be used for selecting studies (such as two independent reviewers) through each phase of the review (that is, screening, eligibility and inclusion in meta-analysis) | Page 9 |
| Data collection process | 11c | Describe planned method of extracting data from reports (such as piloting forms, done | Page 8-9 (and supplement) |

| Section and topic | Item No | Checklist item | Location in manuscript |
|---|---|---|---|
| | | independently, in duplicate), any processes for obtaining and confirming data from investigators | |
| Data items | 12 | List and define all variables for which data will be sought (such as PICO items, funding sources), any pre-planned data assumptions and simplifications | Page 10-12 (and supplement) |
| Outcomes and prioritization | 13 | List and define all outcomes for which data will be sought, including prioritization of main and additional outcomes, with rationale | Page 10-12 |
| Risk of bias in individual studies | 14 | Describe anticipated methods for assessing risk of bias of individual studies, including whether this will be done at the outcome or study level, or both; state how this information will be used in data synthesis | Page 12 |
| Data synthesis | 15a | Describe criteria under which study data will be quantitatively synthesised | Page 12-13 |
| | 15b | If data are appropriate for quantitative synthesis, describe planned summary measures, methods of handling data and methods of combining data from studies, including any planned exploration of consistency (such as $I^2$, Kendall's $\tau$) | Page 12-13 |
| | 15c | Describe any proposed additional analyses (such as sensitivity or subgroup analyses, meta-regression) | Page 12-13 |
| | 15d | If quantitative synthesis is not appropriate, describe the type of summary planned | NA |
| Meta-bias(es) | 16 | Specify any planned assessment of meta-bias(es) (such as publication bias | Page 13 |

| Section and topic | Item No | Checklist item | Location in manuscript |
|---|---|---|---|
| | | across studies, selective reporting within studies) | |
| Confidence in cumulative evidence | 17 | Describe how the strength of the body of evidence will be assessed (such as GRADE) | Page 12 |

For peer review only

BMJ Open

# Effects of Behavioral Parent Training for children with attention-deficit/hyperactivity disorder on Parenting Behavior: A protocol for an Individual Participant Data Meta-analysis



| | |
|---|---|
| Journal: | *BMJ Open* |
| Manuscript ID | bmjopen-2020-037749.R1 |
| Article Type: | Protocol |
| Date Submitted by the Author: | 30-Jun-2020 |



| | |
|---|---|
| Complete List of Authors: | Steenhuis, Laura; University of Groningen, Department of Child and Adolescent Psychiatry<br>Groenman, Annabeth; University Medical Center Groningen, Accare, child and adolescent psychiatry<br>Hoekstra, Pieter J; University of Groningen<br>Hornstra, Rianne; University Medical Center Groningen, Accare, child and adolescent psychiatry<br>Luman, Marjolein; VU medisch centrum,<br>van der Oord, Saskia; KU Leuven, Clinical Psychology<br>van den Hoofdakker, Barbara; University Medical Center Groningen, Accare, child and adolescent psychiatry |



| | |
|---|---|
| <b>Primary Subject Heading</b>: | Mental health |
| Secondary Subject Heading: | Paediatrics |
| Keywords: | Child & adolescent psychiatry < PSYCHIATRY, MENTAL HEALTH, PAEDIATRICS, PSYCHIATRY |
| | |


SCHOLARONE™
Manuscripts

I, the Submitting Author has the right to grant and does grant on behalf of all authors of the Work (as defined in the below author licence), an exclusive licence and/or a non-exclusive licence for contributions from authors who are: i) UK Crown employees; ii) where BMJ has agreed a CC-BY licence shall apply, and/or iii) in accordance with the terms applicable for US Federal Government officers or employees acting as part of their official duties; on a worldwide, perpetual, irrevocable, royalty-free basis to BMJ Publishing Group Ltd ("BMJ") its licensees and where the relevant Journal is co-owned by BMJ to the co-owners of the Journal, to publish the Work in this journal and any other BMJ products and to exploit all rights, as set out in our licence.

The Submitting Author accepts and understands that any supply made under these terms is made by BMJ to the Submitting Author unless you are acting as an employee on behalf of your employer or a postgraduate student of an affiliated institution which is paying any applicable article publishing charge ("APC") for Open Access articles. Where the Submitting Author wishes to make the Work available on an Open Access basis (and intends to pay the relevant APC), the terms of reuse of such Open Access shall be governed by a Creative Commons licence – details of these licences and which Creative Commons licence will apply to this Work are set out in our licence referred to above.

Other than as permitted in any relevant BMJ Author's Self Archiving Policies, I confirm this Work has not been accepted for publication elsewhere, is not being considered for publication elsewhere and does not duplicate material already published. I confirm all authors consent to publication of this Work and authorise the granting of this licence.

For peer review only

# Effects of Behavioral Parent Training for children with attention-deficit/hyperactivity disorder on Parenting Behavior: A protocol for an Individual Participant Data Meta-analysis

**Laura A. Steenhuis°**, University of Groningen, University Medical Center Groningen, Department of Child and Adolescent Psychiatry, Groningen, the Netherlands
**Annabeth P. Groenman°,** University of Groningen, University Medical Center Groningen, Department of Child and Adolescent Psychiatry, Groningen, the Netherlands
**Pieter J. Hoekstra,** University of Groningen, University Medical Center Groningen, Department of Child and Adolescent Psychiatry, Groningen, the Netherlands
**Rianne Hornstra,** University of Groningen, University Medical Center Groningen, Department of Child and Adolescent Psychiatry, Groningen, the Netherlands
**Marjolein Luman*,** Department of Clinical, Neuro, and Developmental Psychology, Vrije Universiteit Amsterdam, Amsterdam, The Netherlands. De Bascule, academic centre for child and adolescent psychiatry, The Netherlands.
**Saskia vd Oord*,** Department of Clinical Psychology, KU Leuven; Developmental Psychology, University of Amsterdam
**Barbara J. vd Hoofdakker*,** University of Groningen, University Medical Center Groningen, Department of Child and Adolescent Psychiatry, Groningen, the Netherlands; University of Groningen, Department of Clinical Psychology and Experimental Psychopathology, Groningen, The Netherlands

**° shared first author, authors made equal contributions to the project.**
* Shared last author, authors made equal contributions to the project.

**Corresponding Author:** Annabeth P. Groenman,

Address: Accare, Department of Child and Adolescent Psychiatry, Freepost 176, 9700 VB Groningen, the Netherlands

E-mail address: a.groenman@gmail.com

**Word count main body: 3999 [max 4000]**
**Word count abstract: 290 [max 300]**

For peer review only

**Conflicts of interest:**
APG, LAS, PJH, RH, ML, SVDO, BvdH have no conflicts of interest to report.

**Funding:** This research was funded by The Dutch Organization for Health Research and Development (ZonMw) under grant number 729300013. The funder had no role in the design of this protocol. The funder will have no input on the collection of data, the data analysis or the interpretation or publication of the study results.

**Author contributions:** BvdH is the guarantor. APG wrote the first draft of the manuscript, and LAS wrote a revised and final version. APG, RH and LAS are performing the systematic search and data extraction. PJH, ML, SvdO, BvdH acquired funding for the PAINT-IPDMA project; an international database including individual participant data from RCTs examining psychosocial interventions for the treatment of ADHD in children. The current study is a part of the PAINT-IPDMA project. PJH, ML, SvdO, BvdH, and APG setup the initial design for the IPDMA. All authors worked together to design this IPDMA, contributed to the intellectual content of the current manuscript and approved the final version.

**Acknowledgements**
We would like to thank all collaborators of the PAINT-IPDMA project for providing input to this protocol and for providing data for this project. We thank our parents of clients' expert panel for their valuable input during the conception of the idea for the PAINT-IPDMA.

**Exclusive License**
I, the Submitting Author have the right to grant and do grant on behalf of all authors of the Work (as defined in the below author licence), an exclusive licence and/or a non-exclusive licence for contributions from authors who are: i) UK Crown employees; ii) where BMJ has agreed a CC-BY licence shall apply, and/or iii) in accordance with the terms applicable for US Federal Government officers or employees acting as part of their official duties; on a worldwide, perpetual, irrevocable, royalty-free basis to BMJ Publishing Group Ltd ("BMJ") its licensees and where the relevant Journal is co-owned by BMJ to the co-owners of the Journal, to publish the Work in BMJ Open and any other BMJ products and to exploit all rights, as set out in our licence.

The Submitting Author accepts and understands that any supply made under these terms is made by BMJ to the Submitting Author unless you are acting as an employee on behalf of your employer or a postgraduate student of an affiliated institution which is paying any applicable article publishing charge ("APC") for Open Access articles. Where the Submitting Author wishes to make the Work available on an Open Access basis (and intends to pay the relevant APC), the terms of reuse of such Open Access shall be governed by a Creative Commons licence – details of these licences and which Creative Commons licence will apply to this Work are set out in our licence referred to above.



For peer review only - http://bmjopen.bmj.com/site/about/guidelines.xhtml

**Abstract**

Introduction: Behavioral Parent Training (BPT) is a well-established treatment for children with attention-deficit/hyperactivity disorder (ADHD). BPT is based on the hypothesis that improvements in parenting are mediators of improvements in children's behaviors. However, meta-analyses show considerate heterogeneity in effects of BPT on child outcomes, and meta-analyses on parenting outcomes are scarce. Also, few studies have investigated parenting factors as mediators of child outcomes. This study aims to examine the effects and moderators of BPT on parenting outcomes and whether improvements in parenting mediate amelioration of behavior and impairment in children with ADHD.

Methods and analyses: We will conduct an individual participant data meta-analysis (IPDMA), making use of individual data of existing trials, and giving the opportunity for highly powered moderator analyses. This IPDMA will be performed by the Psychosocial ADHD INTervention (PAINT) collaboration. We will include randomized controlled trials of BPT, for individuals with ADHD below 18 years old. Systematic searches have been performed to locate relevant papers. Authors are currently contacted to share their data with the PAINT-IPDMA project. We will examine effects of BPT on parenting outcomes (e.g., positive and negative parenting, management of affect, perceived parenting competence, parenting stress), moderators of these effects (e.g., parental depression, parenting stress, severity of the child's ADHD symptoms) and subsequently perform mediation analyses where parenting outcomes are modelled as mediators of child outcomes (e.g., symptoms and severity of ADHD, comorbid psychopathology, and impairment).

Ethics and Dissemination: We will include data from RCTs for which ethical approval has been received and consent forms have been signed. De-identified data will be provided by the original investigators. We aim to disseminate our findings through peer-reviewed scientific journals, presentations at (inter)national scientific meetings, newsletters, the website of our project and the Dutch academic workspace ADHD.

For peer review only

## Article summary

*Strengths and limitations of this study:*

- To our knowledge this is the first IPDMA that examines the effects of behavioral parent training for ADHD on parenting outcomes.
- By using IPDMA, we are able to conduct highly powered moderation analysis.
- By collecting individual participant data from randomized controlled trials, we have the opportunity to perform uniform analyses across studies.
- Our IPDMA includes only variables that are reported consistently across the majority of studies in the database.
- Representativeness of the database will depend partly on the willingness and ability of investigators to share their data.

For peer review only



For peer review only - http://bmjopen.bmj.com/site/about/guidelines.xhtml

## Introduction

Behavioral parent training (BPT) is a well-established intervention for children with attention-deficit/hyperactivity disorder (ADHD) and recommended as first line treatment by many international guidelines [1–3]. In BPT, parents are trained to apply behavioral techniques meant to increase prosocial and adaptive child behaviors and to reduce disruptive and maladaptive child behaviors. In addition, BPT focusses on nurturing and positive parent-child relationships [4]. Several meta-analyses on BPT for children with ADHD have shown medium to large effect sizes on parent reported reductions of ADHD symptoms, comorbid problems, impairment and parenting behaviors [3,5–7]. However, the full range of relevant parenting outcomes of BPT has not been assessed in meta-analyses [7,8]. Moreover, while improvements in parenting behaviors are thought to mediate improvements in children's behaviors and associated impairments [3,9], studies investigating these mediation effects are scarce. Another unresolved issue is that individual studies commonly lack the statistical power to adequately assess moderators of improvements of parenting outcomes. Knowing which moderators impact the effectiveness of BPT for child [5] and parenting outcomes [3,6], in addition to knowing which mediators drive the effects of BPT will give more insight into for whom BPT works best and into the working mechanisms underlying this, therefore contributing to personalized treatment of children with ADHD.

BPT aims to increase the demonstration of positive parenting behaviors and to inhibit negative parenting behaviors, improve the management of affect, reduce parenting stress and enhance parenting self-efficacy [9], in order to positively influence children's behaviors and decrease impairment. Parents are trained to modify environmental antecedents (e.g., providing structure) and consequences (e.g., positive rewards for adaptive behavior) of behavior. Positive parenting behaviors consist of providing praise, encouragement, effective communication, demonstrating positive affect and  offering physical affirmations [10,11], whereas negative parenting behaviors are described as providing inconsistent discipline, verbal criticism, corporal punishment, poor supervision, and demonstrating negative affect [12–14]. Essential to positive parenting behavior is the effective management of parental affect, which is often a specific target of BPT [15]. Parents are taught to express positive emotions (e.g., love, affection and warmth) and to inhibit expressions of negative and unsupportive emotions (e.g., anger, frustration and irritability) [16]. Parenting stress (i.e. stress arising from the feeling that the demands of parenting outweigh the resources [17]) is often reported by parents of children with ADHD [18] and is also an important target of BPT. Lastly, parenting self-efficacy is an important target of BPT [19]; parents should feel more confident and competent in carrying out their parenting tasks.



For peer review only - http://bmjopen.bmj.com/site/about/guidelines.xhtml

When assessing parenting outcomes of BPT, it is important to consider a range of outcomes, particularly positive and negative parenting behaviors, management of affect, parenting stress and parenting self-efficacy. However, treatment trials of BPT have not uniformly assessed all parenting outcomes that are explicit targets of BPT [3,6], although BPT outcomes may differ for these different domains. For example, a recent systematic review investigated multiple parenting outcomes (display of parental affect, parenting stress, and parenting self-efficacy) of BPT and showed positive results for outcomes most closest to the target of BPT (e.g., parenting self-efficacy), but less so for more distal outcomes (e.g., parental psychopathology) [20]. Notably, studies in that review were not specifically conducted in ADHD samples, nor were outcomes quantified using a (individual participant data) meta-analytic approach, which will be the analytic approach in the current study. Recently, two meta-analyses examined the effectiveness of behavioral interventions (mostly consisting of BPT) on parenting outcomes in samples of children with ADHD, either assessed by raters unblinded to the treatment condition (e.g., parents involved in BPT [9]) or by blinded raters (e.g., independent raters coding video-taped interactions between the parent and child [3,6]). The first meta-analysis [3] considered effects on positive and negative parenting behaviors and parenting self-efficacy, immediately post-intervention. Results indicated improvements in positive parenting behaviors (medium effect sizes (ES) of 0.68 and 0.63 for unblinded and blinded raters, respectively), reductions in negative parenting behaviors (medium to small ES of 0.57 and 0.43 for unblinded and for blinded raters, respectively), and improvements in parenting self-efficacy (small ES of 0.37 for unblinded raters). The other meta-analysis [6] investigated the effects of BPT for preschool children with (or at risk for) ADHD, and reported medium to small ES of 0.63 and 0.33 by unblinded and blinded raters, respectively, for reductions in negative parenting behaviors. In addition, long-term effects (up to 12 months) on negative parenting behaviors were reported by unblinded raters with an ES of 0.12 (very small). Overall, both unblinded and blinded parent outcomes show improvements after BPT, although the effects on blinded outcomes generally appeared somewhat smaller than the effects on unblinded outcomes. Improvements on other relevant domains such as parenting stress and display of parental affect have not been addressed in meta-analyses, although individual studies demonstrated that parenting stress can be effectively alleviated with BPT [19], and that mothers who participated in BPT have less negative affect and better emotion regulation abilities post-treatment [21].

Heterogeneity in effect sizes is common in meta-analyses investigating parenting outcomes of BPT for children with ADHD [3,6]. More knowledge on factors associated with treatment effects will yield more insight into for whom BPT works best, and may allow clinicians to make better treatment choices tailored to individuals. So far, very few randomized control trials (RCTs) were adequately powered for moderator analyses, and existing moderator analyses were mostly limited to



For peer review only - http://bmjopen.bmj.com/site/about/guidelines.xhtml

child behavioral outcomes [22–24]. While traditional methods of addressing heterogeneity in meta-analyses (such as excluding extreme ES, subgroup analyses, or meta-regression) may resolve heterogeneity (i.e. reduce $I^2$), they reveal little about the cause of this heterogeneity. Individual participant data meta-analysis (IPDMA) includes data at an individual level rather than at study level, which enables exploration of moderators, therefore yielding more information about the cause of heterogeneity. Moreover, IPDMA performs a uniform analysis across all studies. IPDMA also has enough power to perform subgroup analyses, which most individual RCTs lack. Due to the collaborative nature of an IPDMA, collaborators can provide input on all phases of the research (including design, analyses, interpretation and manuscript preparation), leading to a high quality product [25–27]. So far, no IPDMA has been conducted for parenting outcomes of BPT for children with ADHD.

In this paper, we present our protocol for an IPDMA on BPT for children with ADHD. The current IPDMA will explore several child- and parent-related moderators on parenting outcomes of BPT. Given that the examination of moderators on parenting outcomes is scarce, the choice of possible moderators to be investigated in this IPDMA will also be drawn from BPT trials on child outcomes. First, age of the child might be an important moderator [28], as parents may have more influence on younger children, and younger children may have less engrained symptoms. A meta-analysis indeed demonstrated that BPT had more effect on positive parenting behaviors for younger compared to older children with ADHD [3]. Second, medication use of the child may moderate BPT outcomes, as parents of children on medication might find it easier to adapt their parenting styles and experience less resistance when doing so, although there could also be a floor effect as that children with medication might already function better. Previous studies suggested that medication use may positively contribute to BPT outcomes on child's ADHD symptoms [29], although results have been mixed [22]. Third, it seems plausible that intelligence of the child is positively associated with a treatment that involves learning associations between behaviors and consequences and new skills [30]. There is some evidence that higher children's IQ is associated with more improvement in ADHD symptoms following behavioral treatment, but only for specific subgroups of children, such as girls with more anti-social symptoms [24,31,32]. Fourth, pretreatment ADHD severity and presence of comorbidities could moderate BPT, as parents might find it easier to change their parenting behavior when the child has less complex symptomatology. Previous individual studies have indeed confirmed that comorbidities at baseline negatively impacted the outcome of BPT treatment with regard to child symptoms [22,23,33].

There are several parent-related moderators that may have an effect on parenting outcomes of BPT. First, parental mental health problems (depression, ADHD, parental stress) are likely to affect how well parents are able to grasp new information and impact their ability to learn new methods of



For peer review only - http://bmjopen.bmj.com/site/about/guidelines.xhtml

parenting behaviors and skills [22,23,34–36]. Second, low socio-economic status (SES) and limited parental education may moderate outcomes of BPT, as this may increase family strain and impact the availability of family resources. There is mixed evidence of the moderating effect of low SES on the effects of BPT on ADHD symptoms and related problems, showing either no effect [24], or better outcomes for parents with lower education [37]. Effects of low SES in relation to parenting outcomes of BPT have not been investigated. Third, single parenthood may also moderate outcomes of BPT, as individual studies have demonstrated that single mothers are less likely to respond to BPT [38,39], and maintain treatment gains over time [40] in terms of parenting behavior. Indeed, research suggests that single parents benefit more from enhanced BPT programs, suited to their specific needs (STEPP; [41,42]), compared to standard BPT programs. Fourth, there is evidence that lower parenting self-efficacy at baseline has a negative impact on improvements in behavioral problems in children with ADHD following BPT [43] and one could expect similar results for parenting outcomes. On the other hand, it is also possible that lower parenting abilities at baseline yields more room for improvement in BPT, and thus both directions of the effect could be expected.

Following the investigation of parenting outcomes of BPT as part of our IPDMA, the next step will be to examine mediators of improvements in child's behavior following BPT. Currently, there are no meta-analyses analyzing whether improvements in parenting behavior mediate improvements of BPT on symptoms of ADHD of the child. Some evidence of individual studies shows that reductions in parenting behaviors or parenting attributions mediate improvements on child outcomes in behavioral interventions [12,35,44]. This IPDMA aims to synthesize the available data regarding the association between improvements in parent outcomes and improvements in child's outcomes following BPT.

The specific aims of our IPDMA include:

(1) to investigate effects of BPT on parenting outcomes (positive and negative parenting behaviors, display of parental affect, parenting stress and parenting self-efficacy). Given that BPT for ADHD has shown different effects when assessed by unblinded raters and by more blinded raters [3,6,7], we aim to distinguish (if possible) between unblinded and blinded assessments of parenting behavior and display of parental affect;

(2) to investigate possible child (including age, medication use, IQ, ADHD severity, presence and severity of comorbidities) and parent (including depression, ADHD, socio-economic status, single parenthood, and parenting measures) moderators of parenting outcomes;

(3) to investigate whether improvements in parenting behaviors mediate the effect of BPT on behavioral child outcomes and impairment following treatment.

For aims 1-3, outcomes of BPT will be examined immediately post-treatment and at long-term where possible.



For peer review only - http://bmjopen.bmj.com/site/about/guidelines.xhtml

**Methods and Analysis**

For this IPDMA we will build on the Psychosocial ADHD INTervention IPD (PAINT-IPD) database which is registered in prospero: https://www.crd.york.ac.uk/PROSPERO/display_record.php?ID=CRD42017069877&ID=CRD42017069877. In this project we collect data on psychosocial treatments for children with ADHD: the search is regularly updated and the database continues to expand. For the purpose of the current IPDMA on parenting outcomes, we will use the same methods and search strategy. This protocol is written in line with the PRISMA-P 2015 checklist [45] (see supplementary checklist). The current study is planned to commence September 2020.

Inclusion criteria

We will include RCTs of behavioral treatments of individuals aged below 18 with ADHD, corroborated by clinical cutoffs on questionnaires or (semi)-structured interviews. We will include studies that compared BPT with a control condition (i.e., all conditions that are labeled control, including active treatment), and studies that compared BPT to another behavioral intervention (head to head comparisons). We excluded studies or intervention arms that used optimized medication treatment next to BPT as part of their study design or as a control condition. We define BPT as interventions directed at changing children's behaviors (i.e., increasing desirable behaviors and decreasing undesirable behaviors), using (cognitive) behavioral therapeutic techniques which parents are trained in [46]. Multimodal interventions (consisting of both parent and/or teacher and/or child training), will be included if the time spent on parent training within the intervention was at least equal to other types of training.

Selection and screening of studies

The last systematic search was performed on August 1st 2019. Currently, we are contacting the authors of the newly identified studies. Two authors (AG and RH) performed the selection and screening of studies, disagreement was resolved by consensus. A two-step approach to identifying relevant articles was used. First, Medline, CINAHL, PsycINFO, EMBASE+EMBASE CLASSIC, ERIC, Web of Science [Science Citation Index Expanded] was searched for relevant papers using a combination of the following search terms and their synonyms, as well as hierarchical family form (e.g., MeSH terms): treatment specific terms (e.g., behavioral treatment, psychosocial treatment, and parent training), ADHD, child, and randomized controlled trial. No date restrictions were applied. English, German and Dutch language publications published in peer-reviewed journals were included.



For peer review only - http://bmjopen.bmj.com/site/about/guidelines.xhtml

Second, literature lists of all selected studies and relevant systematic reviews and meta-analyses were hand-searched to identify possible missing articles (complete search criteria for each database are available in Supplement 1).

**Data collection and management**

Author contact

We will contact the corresponding authors of all eligible trials to ask for their participation in the current IPD meta-analyses. If after several weeks we have had no response, we will send a reminder to the corresponding author. If we have failed to establish contact with the corresponding author, we will email the other authors of the study. Furthermore, we will contact researchers during conferences, and through our personal network to retrieve all eligible databases.

Data format and management

Part of the data has already been received in light of our previous IPDMA [5]. For newly identified data that we have not yet received, we will use the best, safest way to transfer the data. The most convenient way for most authors will be to transfer the encrypted data per email to the project management, but we will be open to other options (e.g., face to face transfer). A copy of our data collection manual for the PAINT-IPD database can be found in Supplement 2. We will allow authors to send the data in all possible formats, although the most preferable format would be one in which each subject represents a row and each variable a column.

If authors are not included in the PAINT collaborators group yet [5], we will offer one or two authors of each included study a place in our PAINT collaborators group. We plan yearly telephone calls and/or meetings at large international conferences to keep the working group up to date and discuss design and methodological issues. Members of the working group who have provided data on BPT trials with parenting outcomes will have the opportunity to provide feedback on the first draft of the manuscript and will be sent a copy of the final manuscript before submission. Only authors in the IPD steering committee group (APG, BvdH, SvdO, ML and PH) will have access to the data.

Ethics and Dissemination

The original investigators will be asked for de-identified data, so that only the original investigator knows the link between data and participant. We will only include RCTs where ethical approval has been given and participants signed consent forms. Since the current IPDMA is an extension of the original purpose of the eligible studies, we do not expect any ethical issues with the current IPDMA. Results of our study will be disseminated through peer-reviewed scientific journals, and presentations on (inter)national scientific and/or clinical expert meetings.



For peer review only - http://bmjopen.bmj.com/site/about/guidelines.xhtml

Patient and Public Involvement

An expert panel consisting of parents of children with ADHD was organized at the conception of the idea in which feedback was given to the plans and changes were made accordingly. Our results will be communicated to clinicians, clients and their parents through newsletters, and through the academic workplace "ADHD en druk gedrag" (also see https://adhdendrukgedrag.nl/) in which many parents, client organizations, and mental health care professionals are represented.

Variables

For the PAINT-IPD database, a data request form will be sent to all authors of the original studies, containing a list of variables that will be requested (see Supplement 2a). This list was determined by reviewing the literature and the IPDMA steering committee (authors APG, SvdO, ML, PH and BvdH) assessed these outcomes domains for suitability and interest. The final list of variables will depend on the available data of all studies.

For the current protocol, the following parenting variables will be selected:

*Positive and negative parenting behaviors* will be assessed using unblinded and blinded measures. For unblinded parenting measures, we will select questionnaires such as the Alabama Parenting Questionnaire [10]. For blinded measures, we will select assessments used to observe and code parent behavior, such as the Dyadic Parent-Child Interaction Coding System [47].

*Display of Positive and negative parental affect* will be assessed with measures specifically designed to capture parental affect (e.g., 5 min speech sample of expressed emotion; [15]) or a subscale of an existing parenting scale (e.g., attachment domain of the Parenting-Relationship Questionnaire [48]).

*Parental stress* will be assessed using measures specifically designed to capture stress from parenting, such as the Parenting Stress Index (PSI; [49]), but also measures assessing stress in the caregiver more generally (Depression and Anxiety Stress Scales, DASS; [50]).

*Parenting self-efficacy* will be assessed using measures specifically designed to capture parenting self-efficacy, such as the Parental Sense of Competence Scale (PSOC; [51]), a subscale of an existing parenting scale (e.g., the Parenting-Relationship Questionnaire, [48]), or a scale designed to assess parenting competence in specific contexts, such as education (e.g., Parent as Educator Scale, [52]).

*Parental depression* will be assessed using measures to capture depressive symptoms, such as the Beck Depression Inventory (BDI; [53]), a subscale of an existing mental health questionnaire (e.g., depression subscale of the Depression and Anxiety Stress Scales, DASS; [54]) or a mental health



For peer review only - http://bmjopen.bmj.com/site/about/guidelines.xhtml

questionnaire for which the overall score can be used as a proxy for depression (e.g., the General Health Questionnaire, GHQ; [55]).

*Parental ADHD* will be assessed using an adult measure of ADHD, such as the Adult Self-Report Scale (ASRS) Screener [56].

The remaining parent variables, including *single parenthood* and *socio-economic status (SES),* are commonly assessed with demographic questionnaires or items. SES will be conceptualized in line with previous work of our group and following recommendations in health research [5,57], as low (<high school), medium (high school graduate or college education), or high (>college graduate).

Note that *parenting behaviors, parental affect, parental stress* and *parenting self-efficacy* will serve as moderators (to predict parenting outcomes), mediators (in the relationship between BPT and child outcomes) and outcome variables (in the moderator analysis). *Parental depression, parental ADHD, single parenthood and socio-economic status* will serve as baseline moderators to predict parenting outcomes.

Additionally the following child variables will be selected:

*Child ADHD severity* will be assessed using a parent-rated measure of childhood ADHD, such as the ADHD subscale of the Connors Parent Rating Scale [58].

*Comorbidity* will be assessed using symptoms of Oppositional Defiant Disorder (ODD) and/or Conduct Disorder (CD), by for example the ODD subscale of the Connors Parent Rating Scale [58], or the CD subscale of the Disruptive Behavior Disorder Rating Scale [59]. In addition, internalizing symptoms will be assessed, using for example the internalizing subscale of the Child Behavior Checklist [60].

*Global impairment* will be assessed using a parent- or clinician-rated measure of global impairment, such as the Impairment Rating Scale [61].

The remaining child variables, including *child age, medication use and IQ* are commonly assessed with demographic questionnaires or items.

*Child ADHD severity, comorbidity and global impairment* will serve as moderators (to predict parenting outcomes) and outcome variables (in the mediation analysis). *Child age, medication use and IQ* will solely serve as moderators to predict parenting outcomes.

For all variables (moderators and outcomes), when a study reports multiple measures to capture the same concept, the measure which is most often used by other studies will be included. If multiple raters (mothers and fathers) are included to assess the same concept, the assessment by the mother will be preferred as they are more often the primary caregiver and more often take part in BPT. Regarding harmonization, for each dataset continuous measures will be converted into z-scores, using pre-intervention-score standard deviations within studies.



For peer review only - http://bmjopen.bmj.com/site/about/guidelines.xhtml

Quality assessment

Quality assessment will be done independently by three authors (a combination of AG, RH and LAS) using Cochrane risk of bias. Any disagreement will be resolved by consensus. Once the data has been received, all raw data sets will be checked for impossible, missing, or extreme values. We will collect data on all randomized participants, and subsequently adopt the intention to treat principle. As this will possibly reintroduce participants who were previously excluded, we will also check randomization parameters (e.g., age, gender, and ADHD severity of the participants). If any unexpected deviations are found between our results and the published results, the original researcher will be contacted to locate the origin of this deviation.

Analysis

Effects of BPT will be calculated using a one-stage IPDMA, in which data from participants across studies will be analyzed in one stage, clustered by study. A linear multilevel analysis will be used to examine the effects of BPT on parenting outcome measures. A random intercept for study will be added to each model. Post intervention outcome measures will be used as dependent variables in these models, and pre-intervention outcome measures and intervention group will be added. The interaction between intervention group and moderators of interest will be added to the model to assess their moderating effect on treatment outcomes. To analyze changes in parenting behaviors as a potential mediator in the relationship between BPT and child outcomes, the change in parenting behaviors (post-treatment score – baseline score) will be used. An interaction between intervention group and change in parenting behaviors will be added to the model to determine whether change in parenting behaviors had a main effect on child outcomes and/or an interactive effective with treatment [62,63]. We will conduct sensitivity analyses between studies that provided data and those that did not, for demographic characteristics (e.g., age), and for inclusion criteria (cut-off on measures versus meeting diagnostic criteria for ADHD) and reported effect sizes. If sufficient data is available, a subgroup analysis for child age will be conducted, where all analyses will be repeated separately for pre-school children (<6 years of age), school aged children (6-12 years of age) and adolescents (>12 years of age).

**Discussion**

The current protocol presents the first IPDMA to synthesize research findings on treatment effects of BPT for children with ADHD on parenting outcomes. Both child and parent moderators of parenting outcomes will be explored and a mediation analysis will be conducted to examine whether changes in parenting behaviors mediate the effect of BPT on child behaviors and impairment.



For peer review only - http://bmjopen.bmj.com/site/about/guidelines.xhtml

Previous efforts to identify moderators of parenting outcomes are scarce, as there are few well powered RCTs and only two meta-analyses on this topic [3,6]. The heterogeneity found in parenting outcomes in previous meta-analyses suggest that there is not a “one size fits all” solution of BPT for children with ADHD. Parents and children who differ with regard to their personal, clinical and demographic characteristics, are also likely to differ in their response to BPT. Clearly, an IPDMA approach is needed to further examine the effects of BPT on parenting outcomes, to elucidate potential sources of heterogeneity amongst children and parents, and to investigate potential mechanisms of change in BPT for children with ADHD. Ultimately, both research and clinical practice may be informed by the knowledge of which child and/or parent responds best to a certain treatment, thereby contributing to the overall goal of providing the best care to children with ADHD and their parents.

An additional important aim of the current IPDMA will be to examine a crucial assumption about the working mechanism of BPT, namely whether improvements in parenting behaviors mediate improvements in child behavior and impairment. So far, single studies have examined this assumption, finding some supportive evidence [12,19], but this hypothesis has never been addressed in a meta-analysis, let alone by using an IPDMA approach. By synthesizing all available (raw) data from RCTs, we will conduct highly sophisticated and powered statistical analyses, to provide insight into the working mechanisms of BPT. This knowledge will allow us to further improve and refine BPT programs for children with ADHD.

Despite clear strengths of the IPDMA approach, such as high-powered moderator, mediation and subgroup analyses, there are some limitations as well [25,27,64]. First, IPDMA cannot change anything about the way the study was originally conducted. Second, it is unclear whether enough studies measured the variables of interest, and thus whether all intended outcomes can be examined. Third, if investigators of trials are unwilling to share their data (or may not be able to share their data), not all relevant data can be included in the synthesis. Fourth, not all factors which may affect study outcomes can be conceptualized as a moderator, such as efficacy versus effectiveness trials (i.e., studies often do not fit exactly into one of both categories).

Notwithstanding these limitations, this study has the potential to elucidate clinically relevant questions concerning the efficacy of BPT for children with ADHD and their parents and to provide insight in moderators and mediators of treatment effects. This knowledge may improve and optimize current treatment programs and could eventually lead to advances in personalized treatment for children with ADHD.



For peer review only - http://bmjopen.bmj.com/site/about/guidelines.xhtml

## References


1 Wolraich M, Brown L, Brown RT, *et al.* ADHD: Clinical practice guideline for the diagnosis, evaluation, and treatment of attention-deficit/ hyperactivity disorder in children and adolescents. Pediatrics. 2011. doi:10.1542/peds.2011-2654

2 NICE NI for H and CE. Attention deficit hyperactivity disorder: diagnosis and management (NICE Guideline 87). Natl. Inst. Heal. Care Excell. 2018.

3 Daley D, Van Der Oord S, Ferrin M, *et al.* Behavioral interventions in attention-deficit/hyperactivity disorder: A meta-analysis of randomized controlled trials across multiple outcome domains. J. Am. Acad. Child Adolesc. Psychiatry. 2014. doi:10.1016/j.jaac.2014.05.013

4 Kazdin AE. Parent management training: Evidence, outcomes, and issues. *J Am Acad Child Adolesc Psychiatry* Published Online First: 1997. doi:10.1097/00004583-199710000-00016

5 Groenman AP, Hornstra R, Hoekstra PJ, *et al.* Behavioral treatments for children and adolescents with attention-deficit/hyperactivity disorder: an individual participant data meta-analysis. *Submitted*

6 Rimestad ML, Lambek R, Zacher Christiansen H, *et al.* Short- and Long-Term Effects of Parent Training for Preschool Children With or at Risk of ADHD: A Systematic Review and Meta-Analysis. J. Atten. Disord. 2019. doi:10.1177/1087054716648775

7 Sonuga-Barke EJ, Brandeis D, Cortese S, *et al.* Nonpharmacological interventions for ADHD: Systematic review and meta-analyses of randomized controlled trials of dietary and psychological treatments. Am. J. Psychiatry. 2013. doi:10.1176/appi.ajp.2012.12070991

8 Mulqueen JM, Bartley CA, Bloch MH. Meta-Analysis: Parental Interventions for Preschool ADHD. *J Atten Disord* Published Online First: 2015. doi:10.1177/1087054713504135

9 Daley D, Van Der Oord S, Ferrin M, *et al.* Practitioner review: Current best practice in the use of parent training and other behavioural interventions in the treatment of children and adolescents with attention deficit hyperactivity disorder. *J Child Psychol Psychiatry Allied Discip* Published Online First: 2017. doi:10.1111/jcpp.12825

10 Frick P. The Alabama Parenting Questionnaire. *Unpubl Rat scale, Univ Alabama* 1991.

11 Morris AS, Silk JS, Steinberg L, *et al.* Temperamental vulnerability and negative parenting as interacting predictors of child adjustment. J. Marriage Fam. 2002. doi:10.1111/j.1741-3737.2002.00461.x

12 Hinshaw SP, Owens EB, Wells KC, *et al.* Family processes and treatment outcome in the MTA: Negative/ineffective parenting practices in relation to multimodal treatment. *J Abnorm Child Psychol* 2000;**28**:555–68. doi:10.1023/A:1005183115230

13 Sanders MR. Triple P-Positive Parenting Program as a Public Health Approach to




For peer review only - http://bmjopen.bmj.com/site/about/guidelines.xhtml

Strengthening Parenting. *J Fam Psychol* Published Online First: 2008. doi:10.1037/0893-3200.22.3.506

14 Wells KC, Epstein JN, Hinshaw SP, *et al.* Parenting and Family stress treatment outcomes in attention deficit hyperactivity disorder. *J Abnorm Child Psychol* 2000;**6**:543–53.https://link.springer.com/content/pdf/10.1023%2FA%3A1005131131159.pdf

15 Daley D, Sonuga-Barke EJS, Thompson M. Assessing expressed emotion in mothers of preschool AD/HD children: Psychometric properties of a modified speech sample. *Br J Clin Psychol* Published Online First: 2003. doi:10.1348/014466503762842011

16 Herbert SD, Harvey EA, Roberts JL, *et al.* A Randomized Controlled Trial of a Parent Training and Emotion Socialization Program for Families of Hyperactive Preschool-Aged Children. *Behav Ther* 2013;**44**:302–16. doi:10.1016/j.beth.2012.10.004

17 Deater-Deckard K, Smith J, Ivy L, *et al.* Differential perceptions of and feelings about sibling children: Implications for research on parenting stress. Infant Child Dev. 2005. doi:10.1002/icd.389

18 Johnson JH, Reader SK. Assessing stress in families of children with ADHD: Preliminary development of the disruptive behavior stress inventory (DBSI). *J Clin Psychol Med Settings* Published Online First: 2002. doi:10.1023/A:1014136029697

19 Heath CL, Curtis DF, Fan W, *et al.* The Association Between Parenting Stress, Parenting Self-Efficacy, and the Clinical Significance of Child ADHD Symptom Change Following Behavior Therapy. *Child Psychiatry Hum Dev* 2014;**46**:118–29. doi:10.1007/s10578-014-0458-2

20 Colalillo S, Johnston C. Parenting Cognition and Affective Outcomes Following Parent Management Training: A Systematic Review. Clin. Child Fam. Psychol. Rev. 2016. doi:10.1007/s10567-016-0208-z

21 Gershy N, Meehan KB, Omer H, *et al.* Randomized Clinical Trial of Mindfulness Skills Augmentation in Parent Training. *Child Youth Care Forum* 2017;**46**:783–803. doi:10.1007/s10566-017-9411-4

22 Van Den Hoofdakker BJ, Nauta MH, Veen-Mulders L Van Der, *et al.* Behavioral parent training as an adjunct to routine care in children with attention-deficit/hyperactivity disorder: Moderators of treatment response. *J Pediatr Psychol* Published Online First: 2010. doi:10.1093/jpepsy/jsp060

23 Veenman B, Luman M, Oosterlaan J. Moderators influencing the effectiveness of a behavioral teacher program. *Front Psychol* Published Online First: 2018. doi:10.3389/fpsyg.2018.00298

24 Owens EB, Hinshaw SP, Arnold LE, *et al.* Which treatment for whom for ADHD? Moderators of treatment response in the MTA. J. Consult. Clin. Psychol. 2003. doi:10.1037/0022-006X.71.3.540



For peer review only - http://bmjopen.bmj.com/site/about/guidelines.xhtml

For peer review only

25 Riley RD, Lambert PC, Abo-Zaid G. Meta-analysis of individual participant data: Rationale, conduct, and reporting. *BMJ* Published Online First: 2010. doi:10.1136/bmj.c221

26 Higgins JPT, Jackson D, Barrett JK, *et al.* Consistency and inconsistency in network meta-analysis: concepts and models for multi-arm studies. *Res Synth Methods* 2012;**3**:98–110. doi:10.1002/jrsm.1044

27 Stewart LA, Tierney JF. To IPD or not to IPD? Advantages and disadvantages of systematic reviews using individual patient data. Eval. Heal. Prof. 2002. doi:10.1177/016327870202500106

28 Owens EB, Hinshaw SP, McBurnett K, *et al.* Predictors of Response to Behavioral Treatments Among Children With ADHD-Inattentive Type. *J Clin Child Adolesc Psychol* Published Online First: 2018. doi:10.1080/15374416.2016.1228461

29 Jensen, Arnold, Richters, Severe, Vereen V al. (1999). Moderators and mediators of treatment response for children with attention-deficit/hyperactivity disorder: The multimodal treatment study of children with attention-deficit/hyperactivity disorder. *Arch Gen Psychiatry* Published Online First: 1999. doi:10.1001/archpsyc.56.12.1088

30 Hallahan DP. Cognitive-behavioral therapy for impulsive children. *Child Youth Serv Rev* Published Online First: 1986. doi:10.1016/0190-7409(86)90045-9

31 Kazdin AE, Crowley MJ. Moderators of treatment outcome in cognitively based treatment of antisocial children. *Cognit Ther Res* Published Online First: 1997. doi:10.1023/A:1021832729480

32 Van der Oord S, Prins PJM, Oosterlaan J, *et al.* Efficacy of methylphenidate, psychosocial treatments and their combination in school-aged children with ADHD: A meta-analysis. Clin. Psychol. Rev. 2008. doi:10.1016/j.cpr.2007.10.007

33 Lee SS, Falk AE, Aguirre VP. Association of comorbid anxiety with social functioning in school-age children with and without attention-deficit/hyperactivity disorder (ADHD). *Psychiatry Res* Published Online First: 2012. doi:10.1016/j.psychres.2012.01.018

34 Langberg JM, Evans SW, Schultz BK, *et al.* Trajectories and Predictors of Response to the Challenging Horizons Program for Adolescents With ADHD. *Behav Ther* Published Online First: 2016. doi:10.1016/j.beth.2016.01.001

35 Chronis-Tuscano A, O'Brien KA, Johnston C, *et al.* The relation between maternal ADHD symptoms & improvement in child behavior following brief behavioral parent training is mediated by change in negative parenting. *J Abnorm Child Psychol* 2011;**39**:1047–57. doi:10.1007/s10802-011-9518-2

36 Sonuga-Barke EJS, Daley D, Thompson M. Does Maternal ADHD Reduce the Effectiveness of Parent Training for Preschool Children's ADHD? *J Am Acad Child Adolesc Psychiatry* Published



For peer review only - http://bmjopen.bmj.com/site/about/guidelines.xhtml

Online First: 2002. doi:10.1097/00004583-200206000-00009

37 Farmer CA, Brown N V., Gadow KD, *et al.* Comorbid Symptomatology Moderates Response to Risperidone, Stimulant, and Parent Training in Children with Severe Aggression, Disruptive Behavior Disorder, and Attention-Deficit/Hyperactivity Disorder. *J Child Adolesc Psychopharmacol* Published Online First: 2015. doi:10.1089/cap.2014.0109

38 Dumas JE, Wahler RG. Predictors of treatment outcome in parent training: Mother insularity and socioeconomic disadvantage. *Behav Assess* 1983.

39 Webster-Stratton C, Hammond M. Predictors of treatment outcome in parent training for families with conduct problem children. *Behav Ther* Published Online First: 1990. doi:10.1016/S0005-7894(05)80334-X

40 Webster-Stratton C. Predictors of treatment outcome in parent training for conduct disordered children. *Behav Ther* Published Online First: 1985. doi:10.1016/S0005-7894(85)80048-4

41 Chacko A, Wymbs BT, Wymbs FA, *et al.* Enhancing traditional behavioral parent training for single mothers of children with ADHD. *J Clin Child Adolesc Psychol* Published Online First: 2009. doi:10.1080/15374410802698388

42 Chacko A, Uderman JZ, Zwilling A. Lessons Learned in Enhancing Behavioral Parent Training for High-Risk Families of Youth with ADHD. *ADHD Rep* Published Online First: 2013. doi:10.1521/adhd.2013.21.4.6

43 van den Hoofdakker BJ, Hoekstra PJ, van der Veen-Mulders L, *et al.* Paternal influences on treatment outcome of behavioral parent training in children with attention-deficit/hyperactivity disorder. *Eur Child Adolesc Psychiatry* 2014;**23**:1071–9. doi:10.1007/s00787-014-0557-4

44 Booster GD, Mautone JA, Nissley-Tsiopinis J, *et al.* Reductions in negative parenting practices mediate the effect of a family-school intervention for children with attention deficit hyperactivity disorder. *School Psych Rev* Published Online First: 2016. doi:10.17105/SPR45-2.192-208

45 Shamseer L, Moher D, Clarke M, *et al.* Preferred reporting items for systematic review and meta-analysis protocols (prisma-p) 2015: Elaboration and explanation. BMJ. 2015. doi:10.1136/bmj.g7647

46 Evans SW, Owens JS, Bunford N. Evidence-Based Psychosocial Treatments for Children and Adolescents with Attention-Deficit/Hyperactivity Disorder. *J Clin Child Adolesc Psychol* Published Online First: 2014. doi:10.1080/15374416.2013.850700

47 Eyberg SM, Nelson MMD, Duke M, *et al.* Manual for the Dyadic Parent-Child Interaction Coding System Third Edition. *System* 2004.



For peer review only - http://bmjopen.bmj.com/site/about/guidelines.xhtml

48 Rubinic D, Schwickrath H. Test Review: Kamphaus, R. W., & Reynolds, C. R. (2006). Parenting Relationship Questionnaire. Minneapolis, MN: NCS Pearson. J. Psychoeduc. Assess. 2010. doi:10.1177/0734282909346718

49 Abidin RR. Parenting Stress and the Utilization of Pediatric Services. *Child Heal Care* 1982;**11**:70–3. doi:10.1207/s15326888chc1102_5

50 Clara IP, Cox BJ, Enns MW. Confirmatory Factor Analysis of the Depression-Anxiety-Stress Scales in Depressed and Anxious Patients. *J Psychopathol Behav Assess* 2001;**23**:61–7. doi:10.1023/A:1011095624717

51 Ohan JL, Leung DW, Johnston C. The parenting sense of competence scale: Evidence of a stable factor structure and validity. *Can J Behav Sci* Published Online First: 2000. doi:10.1037/h0087122

52 Mautone JA, Marcelle E, Tresco KE, *et al.* Assessing the quality of parent-teacher relationships for students with ADHD. *Psychol Sch* Published Online First: 2015. doi:10.1002/pits.21817

53 Beck AT, Steer RA, Carbin MG. Psychometric properties of the Beck Depression Inventory: Twenty-five years of evaluation. *Clin Psychol Rev* Published Online First: 1988. doi:10.1016/0272-7358(88)90050-5

54 Henry JD, Crawford JR. The short-form version of the Depression Anxiety Stress Scales (DASS-21): construct validity and normative data in a large non-clinical sample. *Br J Clin Psychol* 2005;**44**:227–39. doi:10.1348/014466505X29657

55 Goldberg DP, Hillier VF. A scaled version of the General Health Questionnaire. *Psychol Med* Published Online First: 1979. doi:10.1017/S0033291700021644

56 Kessler RC, Adler L, Ames M, *et al.* The World Health Organization adult ADHD self-report scale (ASRS): A short screening scale for use in the general population. *Psychol Med* Published Online First: 2005. doi:10.1017/S0033291704002892

57 Oakes JM, Rossi PH. The measurement of SES in health research: Current practice and steps toward a new approach. *Soc Sci Med* Published Online First: 2003. doi:10.1016/S0277-9536(02)00073-4

58 Kao GS, Thomas HM. Test Review: C. Keith Conners Conners 3rd Edition Toronto, Ontario, Canada: Multi-Health Systems, 2008. *J Psychoeduc Assess* Published Online First: 2010. doi:10.1177/0734282909360011

59 Pelham Jr. WE, Gnagy EM, Greenslade KE, *et al.* Teacher ratings of DSM-III-R symptoms for the disruptive behavior disorders.[erratum appears in J Am Acad Child Adolesc Psychiatry 1992 Nov;31(6):1177]. *J Am Acad Child Adolesc Psychiatry* 1992.

60 Achenbach TM. *Manual for the Youth Self Report and 1991 Profile*. 1991.

61 Fabiano GA, Pelham WE, Waschbusch DA, *et al.* A practical measure of impairment:




For peer review only - http://bmjopen.bmj.com/site/about/guidelines.xhtml

Psychometric properties of the impairment rating scale in samples of children with attention deficit hyperactivity disorder and two school-based samples. *J Clin Child Adolesc Psychol* Published Online First: 2006. doi:10.1207/s15374424jccp3503_3

62 HC K, GT W, CG F, *et al.* Mediators and moderators of treatment effects in randomized clinical trials. *Arch Gen Psychiatry* 2002.

63 Haasova M, Warren FC, Ussher M, *et al.* The acute effects of physical activity on cigarette cravings: Exploration of potential moderators, mediators and physical activity attributes using individual participant data (IPD) meta-analyses. Psychopharmacology (Berl). 2014. doi:10.1007/s00213-014-3450-4

64 Higgins JPT, Whitehead A, Turner RM, *et al.* Meta-analysis of continuous outcome data from individual patients. *Stat Med* Published Online First: 2001. doi:10.1002/sim.918

For peer review only

Supplement 1- Search terms per database

**Behavioral interventions**

**PubMed**

("Attention Deficit Disorder with Hyperactivity"[Mesh] OR ADHD[tiab] OR ADD[tiab] OR attention deficit*[tiab] OR hyperactiv*[tiab] OR hyperkinetic*[tiab] OR minimal brain deficit*[tiab] OR minimal brain dysfunction*[tiab])

**AND**

(("Psychotherapy"[Mesh] OR psychotherap*[tiab] OR psychological therap*[tiab] OR psychological intervent*[tiab] OR psychoeduca*[tiab] OR mentoring*[tiab] OR coaching*[tiab] OR mindful*[tiab] OR relax*[tiab] OR meditat*[tiab])

OR

((parent*[tiab] OR mother[tiab] OR father[tiab] OR teacher*[tiab] OR school*[tiab])

*AND*

(program*[tiab] OR train*[tiab] OR educa*[tiab] OR therapy*[tiab] OR therapies*[tiab] OR therapeu*[tiab]OR intervention*[tiab] OR coaching*[tiab] OR counseling*[tiab]))

OR

((behavio*[tiab] OR cognit*[tiab] OR “acceptance and commitment”[tiab] OR dialectica*[tiab])

*AND*

(program*[tiab] OR therapy*[tiab] OR therapies*[tiab] OR therapeu*[tiab] OR intervention*[tiab] OR treatment*[tiab] OR train*[tiab]))

OR

(psychosocial[tiab]

AND

(treatment*[tiab] OR therapy*[tiab] OR therapies*[tiab] OR therapeu*[tiab] OR train*[tiab] OR intervention*[tiab] OR program*[tiab]))

OR

((skill*[tiab] OR organization*[tiab] OR organisation*[tiab] OR planning*[tiab] OR play*[tiab])

AND

(train*[tiab] OR intervention*[tiab] OR program*[tiab])))

**AND**

("Adolescent"[Mesh] OR "Child"[Mesh] OR child*[tiab] OR school*[tiab] OR infan*[tiab] OR adolescen*[tiab] OR pediatri*[tiab] OR paediatr*[tiab] OR boy[tiab] OR boys[tiab] OR boyhood[tiab] OR girl[tiab] OR girls[tiab] OR girlhood[tiab] OR youth[tiab] OR youths[tiab] OR teen[tiab] OR teens[tiab] OR teenage*[tiab] OR puberty[tiab] OR preschool*[tiab] OR toddler*[tiab] OR juvenile*[tiab] OR kids[tiab])

**AND**

("Controlled Clinical Trial" [Publication Type] OR "Randomized Controlled Trials as Topic"[Mesh] OR "Follow-Up Studies"[Mesh] OR follow-up[tiab] OR followup[tiab] OR control group*[tiab] OR "Random Allocation"[Mesh] OR random*[tiab] OR trial[ti])

For peer review only

**EMBASE EMBASE.com** (losse delen combineren op website)
('attention deficit disorder'/exp OR (ADHD OR ADD OR 'attention deficit*'
OR hyperactiv* OR hyperkinetic* OR 'minimal brain deficit*' OR 'minimal brain dysfunction*'):ab,ti)
**AND**

('psychotherapy'/exp OR (psychotherap* OR 'psychological therap*' OR 'psychological intervent*'
OR psychoeduca* OR mentoring* OR coaching* OR mindful* OR relax*):ab,ti)
OR
((((parent* OR mother OR father OR teacher* OR school*)
*AND*
(program* OR train* OR educa* OR therapy* OR therapies* OR therapeu*OR intervention*
OR coaching* OR counseling*))
OR
((behavio* OR cognit* OR 'acceptance and commitment' OR dialectica*)
*AND*
(program* OR therapy* OR therapies* OR therapeu* OR intervention* OR treatment* OR
train*))
OR
(psychosocial
AND
(treatment* OR therapy* OR therapies* OR therapeu* OR train* OR intervention* OR
program*))
OR
((skill* OR organization* OR organisation* OR planning* OR play*)
AND
(train* OR intervention* OR program*))):ab,ti)

**AND**
('juvenile'/exp OR (child* OR school* OR infan* OR adolescen* OR pediatri* OR paediatr* OR boy OR
boys OR boyhood OR girl OR girls OR girlhood OR youth OR youths OR teen OR teens OR teenage*
OR puberty OR preschool* OR toddler* OR juvenile* OR kids):ab,ti)

**AND**
('controlled clinical trial'/exp OR 'randomization'/exp OR ('control group*' OR random*):ab,ti OR
trial:ti)

For peer review only

**EBSCO PsycINFO**
(DE "Attention Deficit Disorder" OR DE "Attention Deficit Disorder with Hyperactivity" OR ADHD OR ADD OR “attention deficit*” OR hyperactiv* OR hyperkinetic* OR “minimal brain deficit*” OR “minimal brain dysfunction*”)
**AND**
(DE "Psychotherapy" OR DE "Child Psychotherapy" OR DE "Play Therapy" OR DE "Behavior Therapy" OR DE "Aversion Therapy" OR DE "Conversion Therapy" OR DE "Dialectical Behavior Therapy" OR DE "Exposure Therapy" OR DE "Implosive Therapy" OR DE "Reciprocal Inhibition Therapy" OR DE "Response Cost" OR DE "Systematic Desensitization Therapy" AND DE "Cognitive Behavior Therapy" OR DE "Cognitive Therapy" OR DE "Group Psychotherapy" OR DE "Neurotherapy" OR psychotherap* OR “psychological therap*” OR “psychological intervent*” OR psychoeduca* OR mentoring* OR coaching* OR mindful* OR relax*)
OR
((parent* OR mother OR father OR teacher* OR school*)
*AND*
(program* OR train* OR educa* OR therapy* OR therapies* OR therapeu*OR intervention* OR coaching* OR counseling*))
OR
((behavio* OR cognit* OR ‘acceptance and commitment’ OR dialectica*)
*AND*
(program* OR therapy* OR therapies* OR therapeu* OR intervention* OR treatment* OR train*))
OR
(psychosocial
AND
(treatment* OR therapy* OR therapies* OR therapeu* OR train* OR intervention* OR program*))
OR
((skill* OR organization* OR organisation* OR planning* OR play*)
AND
(train* OR intervention* OR program*))
**AND**
(AG (childhood OR adolescence) OR (child* OR school* OR infan* OR adolescen* OR pediatri* OR paediatr* OR boy OR boys OR boyhood OR girl OR girls OR girlhood OR youth OR youths OR teen OR teens OR teenage* OR puberty OR preschool* OR toddler* OR juvenile* OR kids))
**AND**
(“control group*” OR random* OR "controlled trial" OR "controlled study" OR "experimental study" OR "experimental design" OR TI trial)

**EBSCO CINAHL**
(MH "Attention Deficit Hyperactivity Disorder" OR "Attention Deficit Disorder with Hyperactivity" OR ADHD OR ADD OR “attention deficit*” OR hyperactiv* OR hyperkinetic* OR “minimal brain deficit*” OR “minimal brain dysfunction*”)
**AND**
(MH "Psychotherapy+" OR psychotherap* OR “psychological therap*” OR “psychological intervent*” OR psychoeduca* OR mentoring* OR coaching* OR mindful* OR relax*)
OR
((parent* OR mother OR father OR teacher* OR school*)
*AND*

For peer review only

(program* OR train* OR educa* OR therapy* OR therapies* OR therapeu*OR intervention* OR coaching* OR counseling*))
OR
((behavio* OR cognit* OR ‘acceptance and commitment’ OR dialectica*)
*AND*
(program* OR therapy* OR therapies* OR therapeu* OR intervention* OR treatment* OR train*))
OR
(psychosocial
AND
(treatment* OR therapy* OR therapies* OR therapeu* OR train* OR intervention* OR program*))
OR
((skill* OR organization* OR organisation* OR planning* OR play*)
AND
(train* OR intervention* OR program*))

**AND**

(AG child OR (child* OR school* OR infan* OR adolescen* OR pediatri* OR paediatr* OR boy OR boys OR boyhood OR girl OR girls OR girlhood OR youth OR youths OR teen OR teens OR teenage* OR puberty OR preschool* OR toddler* OR juvenile* OR kids))

**AND**

(MH "Clinical Trials+" OR “control group*” OR random* OR "controlled trial" OR "controlled study" OR "experimental study" OR "experimental design" OR TI trial)

**EBSCO ERIC**

(DE "Attention Deficit Disorder" OR DE "Attention Deficit Hyperactivity Disorder" OR ADHD OR ADD OR “attention deficit*” OR hyperactiv* OR hyperkinetic* OR “minimal brain deficit*” OR “minimal brain dysfunction*”)

**AND**

(DE "Psychotherapy" OR psychotherap* OR “psychological therap*” OR “psychological intervent*” OR psychoeduca* OR mentoring* OR coaching* OR mindful* OR relax*)
OR
((parent* OR mother OR father OR teacher* OR school*)
*AND*
(program* OR train* OR educa* OR therapy* OR therapies* OR therapeu*OR intervention* OR coaching* OR counseling*))
OR
((behavio* OR cognit* OR ‘acceptance and commitment’ OR dialectica*)
*AND*
(program* OR therapy* OR therapies* OR therapeu* OR intervention* OR treatment* OR train*))
OR
(psychosocial
AND
(treatment* OR therapy* OR therapies* OR therapeu* OR train* OR intervention* OR program*))
OR
((skill* OR organization* OR organisation* OR planning* OR play*)
AND

For peer review only

(train* OR intervention* OR program*))

**AND**

(child* OR school* OR infan* OR adolescen* OR pediatri* OR paediatr* OR boy OR boys OR boyhood OR girl OR girls OR girlhood OR youth OR youths OR teen OR teens OR teenager* OR puberty OR preschool* OR toddler* OR juvenile* OR kids))

**AND**

("control group*" OR random* OR "controlled trial" OR "controlled study" OR "experimental study" OR "experimental design" OR TI trial)

**Web of Science**

(TS=("Attention Deficit Disorder with Hyperactivity" OR ADHD OR ADD OR "attention deficit*" OR hyperactiv* OR hyperkinetic* OR "minimal brain deficit*" OR "minimal brain dysfunction*"))

**AND**

(TS=(psychotherap* OR psychotherap* OR "psychological therap*" OR "psychological intervent*" OR psychoeduca* OR mentoring* OR coaching* OR mindful* OR relax*

OR

((parent* OR mother OR father OR teacher* OR school*)

*AND*

(program* OR train* OR educa* OR therapy* OR therapies* OR therapeu*OR intervention* OR coaching* OR counseling*))

OR

((behavio* OR cognit* OR 'acceptance and commitment' OR dialectica*)

*AND*

(program* OR therapy* OR therapies* OR therapeu* OR intervention* OR treatment* OR train*))

OR

(psychosocial

AND

(treatment* OR therapy* OR therapies* OR therapeu* OR train* OR intervention* OR program*))

OR

((skill* OR organization* OR organisation* OR planning* OR play*)

AND

(train* OR intervention* OR program*))))

**AND**

(TS=(child* OR school* OR infan* OR adolescen* OR pediatri* OR paediatr* OR boy OR boys OR boyhood OR girl OR girls OR girlhood OR youth OR youths OR teen OR teens OR teenager* OR puberty OR preschool* OR toddler* OR juvenile* OR kids))

**AND**

(TS=("clinical trial*" OR "control group*" OR random* OR "controlled trial" OR "controlled study" OR "experimental study" OR "experimental design"))

For peer review only

**Cochrane Central register of Controlled Trials** *(CENTRAL)*
("Attention Deficit Disorder with Hyperactivity" OR ADHD OR ADD OR "attention deficit*" OR hyperactiv* OR hyperkinetic* OR "minimal brain deficit*" OR "minimal brain dysfunction*")
**AND**

((psychotherap* OR "psychological therap*" OR "psychological intervent*" OR psychoeduca* OR mentoring* OR coaching* OR mindful* OR relax*)
OR
((parent* OR mother OR father OR teacher* OR school*)
*AND*
(program* OR train* OR educa* OR therapy* OR therapies* OR therapeu*OR intervention* OR coaching* OR counseling*))
OR
((behavio* OR cognit* OR 'acceptance and commitment' OR dialectica*)
*AND*
(program* OR therapy* OR therapies* OR therapeu* OR intervention* OR treatment* OR train*))
OR
((psychosocial)
AND
(treatment* OR therapy* OR therapies* OR therapeu* OR train* OR intervention* OR program*))
OR
((skill* OR organization* OR organisation* OR planning* OR play*)
AND
(train* OR intervention* OR program*)))
**AND**

(child* OR school* OR infan* OR adolescen* OR pediatri* OR paediatr* OR boy OR boys OR boyhood OR girl OR girls OR girlhood OR youth OR youths OR teen OR teens OR teenage* OR puberty OR preschool* OR toddler* OR juvenile* OR kids)

For peer review only

Supplement 2. Data sharing manual

**Data collection form**

**Content**

**Introduction**
**How do I transfer my data?**
**Which participants do we need?**
**Which data do we need?**
**What will we do with the data?**
**Supplement 3A. Measures**

Thank you for taking place in our Psychosocial ADHD INTerverntions (PAINT) collaborators group. We are very grateful that you are willing to put your time into this project. We believe that this group will be fruitful and successful collaboration with the aim to get more insight into psychosocial interventions for ADHD.
With this manual, we hope to provide you with guidance on the data collection. However, if you have any questions regarding the data handling, despite reading this manual, please let us know so we can help you further, and adapt our manual accordingly. You can reach us at paint@accare.nl.

Most of the choices we have made concerning data collection are driven by a need to keep methods and measures similar across trials. Every study is designed slightly different, with different measures, different in- and exclusion criteria, different methods to handle missing data, etc..
To harmonize measures and methods, at some point we will have to make choices regarding data analysis, that differ from the choices made in the original study. For example, we will do intention to treat analysis, while it may be possible that you did not do this in your original analysis. This means that, if available, we will ask for data or participants not included in the original analysis. Furthermore, it is important to mention that we will not report on results from a single trial, although we can report a forestplot displaying all effect sizes of included studies.

How do I transfer my data?
Preferably, we would receive the information electronically. We can work with any kind of data file (STATA, SPSS, etc.), but we prefer a layout, where each line represents a subject.

You can email the data file once you encrypted it using a program such as 7-zip (an explanation on how to do this is included in supplement B). It is important that the password with which you will protect your data file, will be send in a separate email from the email that contains the data file (as attachment). Another important point in transferring the data is that the data should be anonymized. This means that there is no information with which we could trace a single participant back to the individual. This means that no names should be included in the file. However, all other information that can identify a person should be left out of this file. This can mean combinations of variables, for example postal code and date of birth. Please make sure that this kind of information is not present in the data file.  If these kinds of examples arise, please contact us, so we can think with you on what best to do.

Which participants do we need?
We want the data of all included participants. This means all participants that have a baseline measure and were randomized. It could be possible that this is a different approach than you took in

For peer review only

your analysis of the data. Because we want to perform intention to treat analysis, we need all data from included participants.

Which data do we need?

We ask you for clean data (please contact us if this provides you with any questions). We want sum scores and if available norm-scores of questionnaires. Proper labeling is important. In the Table below we provide you with an overview of constructs that we are interested in. It could be possible that the measure or instrument used in your study is not mentioned in this Table, this does not mean that we are not interested, the measures mentioned are mere examples. If there are any remarks on this measure (e.g. certain items were not used) please mention these in the column “remarks”. Please indicate which informants filled out the questionnaires, when multiple informants are used please provide us with data from all informants. If multiple instruments have been used to measure a certain construct, we would really like to receive all instruments (for example a self and a proxy version, or multiple ADHD measures). This could aid us in the harmonization process (see above). If a construct is measured with an in-house instrument or if you made any adjustments on the instrument, could you also provide us this instrument?

For questionnaires, we would like to receive the, sum scores and norm-scores and if available item scores. We would always like to receive the full questionnaire if possible, and not just certain subscales. In the future, it could be possible that we will ask you about norming tables, we hope however, that we will not need these.

For (structured) interviews we would like to receive items scores, sum scores and outcomes. We would like to receive information on the full interview, not only certain outcomes.

Concerning cognitive measures, we would like to receive calculated outcomes variables across trials and conditions, not mere trial to trial performance. For example, in the stop task, we would like to receive measures such as SSRT, mean (and SD) of reaction time on Go trials, number of commissions and omissions, but not the individual performance on a single trial of the stop task. We fill also need information about the task such as number of conditions, number of trials, and how the outcome variables were calculated.

For variables that are available on multiple time points, please provide us with all relevant data. For example, if an ADHD measure is taken at baseline, right after the intervention and after 3 months, we would like to receive this measure assessed at all time points. Again, proper labelling is very important. Please label the data in English (or Dutch).

The constructs and variables that we are interested in for the IPD meta-analysis are shown below. We understand that not all studies have included all measures. The table below will provide you with a list you can keep on hand when preparing the data before sending it. Next to the constructs are examples of instruments. Please remember that this is not a complete list, and that it could be possible that you have used an instrument that is not in our list, but that would still be very valuable for this IPD meta-analysis. Please remember that you do not have to recode your data. We will do this for all datasets.

What will we do with the data?

Once we receive the data we will do some basic data checks. These will include checking missing and impossible values. If we will have any questions concerning the data during or after these checks we will contact you for clarification. After that we will harmonize the data with the other datasets. This harmonization process depends on the available data. For example, this could mean that we have to dichotomize data, or making norm deviation scores (translating raw questionnaire scores on one

scale into raw questionnaire scores on another scale using norm scores). Harmonization is easiest if the same measure has been used across studies. If you have used multiple instruments to measure a certain outcome (e.g. more than 1 ADHD scale), we ask you to provide us both measures. This could mean that we may be able use a latent variable for some measures, or we could calculate correlations between measures.
The original dataset will be saved as the original file. Any harmonization will take place in a separate file, which are only later merged into one big data file.

For peer review only

Supplement 2A.

Tabel 1 Measures.

| **Descriptive measures** | |
|---|---|
| Participant ID | |
| Date of randomization | Dd/mm/yyyy |
| Date of treatment start | Dd/mm/yyyy |
| Date post-intervention measurement | Dd/mm/yyyy |
| Date follow up measurement | Dd/mm/yyyy |
| Date baseline measurement | Dd/mm/yyyy |
| Treatment allocation | 0- Control<br>1- Intervention |
| Age | Age in years |
| Gender | 1-Male<br>2-Female |
| IQ | Norm score of WISC |
| Verbal IQ | Norm score of WISC |
| Performance IQ | Norm score of WISC |
| Ethnicity | |
| Socioeconomic status | |
| ADHD severity at baseline | |
| Comorbidities | ODD/CD/Anxiety/Depression/PDD/Tics/ etc. |
| Number of children living with the family | Total number of children in the household |
| Primary care-taker education | |
| Secundary care-taker education | |
| Household income | Combined income of primary caretaker and (if applicable) secundary caretaker |
| Parental psychopathology (diagnosis) | Parental ADHD/ anxiety/depression/ etc |
| Marital status | |
| Education primary caretaker | |
| Education secondary caretaker | |
| Age Father | Age in years |
| Age mother | Age in years |

For peer review only

| | |
|---|---|
| Psychoactive Medication use pre- intervention | Yes/no, if yes which medication, mg MPH if applicable, x times per day, for the last month, duration |
| Previous care use of the child | Care use in mental health. |
| Medication use during intervention | Yes/no, if yes, mg per day |
| Treatment expectancy | |
| Stable medication use during intervention | Yes/No/NA |
| Other descriptive measures | |
| **Individual child outcomes- behavioral** | |
| **Measured constructs** | **Examples of instruments** |
| **ADHD** | Conners, CBCL, DBD, ACTeRS, ARS, BASC, SNAP, SWAN, Werry Weiss Peters activity scale, Behavioral checklist, PACS, Parent daily report, KSADS, standardized behavioral observations |
| Disruptive disorders/ behavior | Conners, CBCL, DBD, EDBI, SNAP, SSRS, ACTeRS, KSADS, behavior problem checklist, aggression and conduct problems scale, behavioral checklist, PACS, parent daily reports, standardized behavioral observations |
| Social skills | DSAS, Behavior problem checklist, BASC, ACTeRS, SSRS, CBCL |
| Internalizing problems | Conners, CBCL, SSRS, BASC, Behavior problem checklist, MASC, PACS, KSADS, SCARED, CDI |
| Attachment | |
| Quality of life | HONOSCA, kidsscreen, EQ-5d, IRS |
| Selfconcept | Piers-Harris |
| Which other child behavioral outcomes are available? | |
| **Individual child outcomes- academic outcomes** | |
| Measured Construct | Examples of measures |
| Grades on core courses | Math, Spelling, Reading |
| Standardized Academic Achievement tests | Reading, spelling, math scores, woodcock johnson, WRAT, WISC Durrel |
| On and off task behaviors | Classroom observations, SOAPs |
| Academic competence | Academic rating scale, classroom performance survey |

For peer review only

| | |
|---|---|
| Which other academic outcomes are available? | |
| **Individual child outcomes- cognitive outcomes[a]** | |
| Higher cognitive functions | Working memory, executive functions, inhibition, planning, set shifting, reward sensitivity, emotion regulation |
| Intellectual functioning | IQ (WISC, RAVEN) |
| Motor skills | Fine motor skills, precision, gross motor skills |
| Memory | Visuospatial, verbal |
| Basic cognitive functions | Attention, reaction time, speed, perception |
| **Child individual outcomes- other measures** | |
| Adverse events | Types of adverse events reported |
| Treatment fidelity | |
| Treatment satisfaction | |
| Number of sessions attended | |
| Drop out | Yes/no |
| Date of discontinuation of the intervention | |
| Reason for discontinuation | |
| **Parental outcome measures** | |
| Measured constructs | Examples of measures |
| Parental psychopathology | DASS, GHQ, BDI, PSI, CSS, AARS, SCL90, |
| Parent child interaction, parenting behaviors, parenting style, parenting affect | DPICS or other observational measure, parenting practices inventory (PPI), parent interaction style, PS (parenting scale), measures parental sensitivity/reponsivity |
| Parental competence/ self efficacy | PSI-subscale parental competence, PSOC |
| Spouse relationship | locke wallace marital adjustment, PSI-subscale |
| Parental self esteem | Rosenberg self esteem scale (RSE) |
| Parenting stress | PSI |
| Which other parenting outcomes are available? | |

[a] concerning cognitive measures, we would like to receive calculated outcomes measures, not mere trial to trial performance. For example, in the stop task, we would like to receive measures such as SSRT, mean reaction time, number of commissions and omissions, but not the individual performance on a single trial of the stop task. We will also need information on the conditions of the task, how the outcome was calculated, etc.

For peer review only

PRISMA-P (preferred reporting items for systematic review and meta-analysis protocols) 2015 checklist: recommended items to address in a systematic review protocol

| Section and topic | Item No | Checklist item | Location in manuscript |
|---|---|---|---|
| **Administrative information** | | | |
| Title: | | | |
| Identification | 1a | Identify the report as a protocol of a systematic review | Page 1 |
| Update | 1b | If the protocol is for an update of a previous systematic review, identify as such | NA |
| Registration | 2 | If registered, provide the name of the registry (such as PROSPERO) and registration number | Page 8 |
| Authors: | | | |
| Contact | 3a | Provide name, institutional affiliation, e-mail address of all protocol authors; provide physical mailing address of corresponding author | Page 1 |
| Contributions | 3b | Describe contributions of protocol authors and identify the guarantor of the review | Page 2 |
| Amendments | 4 | If the protocol represents an amendment of a previously completed or published protocol, identify as such and list changes; otherwise, state plan for documenting important protocol amendments | NA |
| Support: | | | |
| Sources | 5a | Indicate sources of financial or other support for the review | Page 2 |
| Sponsor | 5b | Provide name for the review funder and/or sponsor | Page 2 |
| Role of sponsor or funder | 5c | Describe roles of funder(s), sponsor(s), and/or institution(s), if any, in developing the protocol | Page 2 |
| **Introduction** | | | |

| Section and topic | Item No | Checklist item | Location in manuscript |
|---|---|---|---|
| Rationale | 6 | Describe the rationale for the review in the context of what is already known | Page 5-8 |
| Objectives | 7 | Provide an explicit statement of the question(s) the review will address with reference to participants, interventions, comparators, and outcomes (PICO) | Page 8 |
| **Methods** | | | |
| Eligibility criteria | 8 | Specify the study characteristics (such as PICO, study design, setting, time frame) and report characteristics (such as years considered, language, publication status) to be used as criteria for eligibility for the review | Page 9 |
| Information sources | 9 | Describe all intended information sources (such as electronic databases, contact with study authors, trial registers or other grey literature sources) with planned dates of coverage | Page 9 |
| Search strategy | 10 | Present draft of search strategy to be used for at least one electronic database, including planned limits, such that it could be repeated | Page 9 (and supplement) |
| Study records: | | | |
| Data management | 11a | Describe the mechanism(s) that will be used to manage records and data throughout the review | Page 10 |
| Selection process | 11b | State the process that will be used for selecting studies (such as two independent reviewers) through each phase of the review (that is, screening, eligibility and inclusion in meta-analysis) | Page 9 |
| Data collection process | 11c | Describe planned method of extracting data from reports (such as piloting forms, done | Page 8-9 (and supplement) |

| Section and topic | Item No | Checklist item | Location in manuscript |
|---|---|---|---|
| | | independently, in duplicate), any processes for obtaining and confirming data from investigators | |
| Data items | 12 | List and define all variables for which data will be sought (such as PICO items, funding sources), any pre-planned data assumptions and simplifications | Page 10-12 (and supplement) |
| Outcomes and prioritization | 13 | List and define all outcomes for which data will be sought, including prioritization of main and additional outcomes, with rationale | Page 10-12 |
| Risk of bias in individual studies | 14 | Describe anticipated methods for assessing risk of bias of individual studies, including whether this will be done at the outcome or study level, or both; state how this information will be used in data synthesis | Page 12 |
| Data synthesis | 15a | Describe criteria under which study data will be quantitatively synthesised | Page 12-13 |
| | 15b | If data are appropriate for quantitative synthesis, describe planned summary measures, methods of handling data and methods of combining data from studies, including any planned exploration of consistency (such as $I^2$, Kendall's $\tau$) | Page 12-13 |
| | 15c | Describe any proposed additional analyses (such as sensitivity or subgroup analyses, meta-regression) | Page 12-13 |
| | 15d | If quantitative synthesis is not appropriate, describe the type of summary planned | NA |
| Meta-bias(es) | 16 | Specify any planned assessment of meta-bias(es) (such as publication bias | Page 13 |

| Section and topic | Item No | Checklist item | Location in manuscript |
|---|---|---|---|
| | | across studies, selective reporting within studies) | |
| Confidence in cumulative evidence | 17 | Describe how the strength of the body of evidence will be assessed (such as GRADE) | Page 12 |

For peer review only

BMJ Open

# Effects of Behavioral Parent Training for children with attention-deficit/hyperactivity disorder on Parenting Behavior: A protocol for an Individual Participant Data Meta-analysis



| | |
|---|---|
| Journal: | *BMJ Open* |
| Manuscript ID | bmjopen-2020-037749.R2 |
| Article Type: | Protocol |
| Date Submitted by the Author: | 21-Aug-2020 |
| Complete List of Authors: | Steenhuis, Laura; University of Groningen, Department of Child and Adolescent Psychiatry<br>Groenman, Annabeth; University Medical Centre Groningen, Accare, child and adolescent psychiatry<br>Hoekstra, Pieter J; University Medical Centre Groningen<br>Hornstra, Rianne; University Medical Centre Groningen, Accare, child and adolescent psychiatry<br>Luman, Marjolein; VU medisch centrum,<br>van der Oord, Saskia; KU Leuven, Clinical Psychology<br>van den Hoofdakker, Barbara; University Medical Centre Groningen, Accare, child and adolescent psychiatry |
| <b>Primary Subject Heading</b>: | Mental health |
| Secondary Subject Heading: | Paediatrics |
| Keywords: | Child & adolescent psychiatry < PSYCHIATRY, MENTAL HEALTH, PAEDIATRICS, PSYCHIATRY |
| | |



SCHOLARONE™
Manuscripts

I, the Submitting Author has the right to grant and does grant on behalf of all authors of the Work (as defined in the below author licence), an exclusive licence and/or a non-exclusive licence for contributions from authors who are: i) UK Crown employees; ii) where BMJ has agreed a CC-BY licence shall apply, and/or iii) in accordance with the terms applicable for US Federal Government officers or employees acting as part of their official duties; on a worldwide, perpetual, irrevocable, royalty-free basis to BMJ Publishing Group Ltd ("BMJ") its licensees and where the relevant Journal is co-owned by BMJ to the co-owners of the Journal, to publish the Work in this journal and any other BMJ products and to exploit all rights, as set out in our licence.

The Submitting Author accepts and understands that any supply made under these terms is made by BMJ to the Submitting Author unless you are acting as an employee on behalf of your employer or a postgraduate student of an affiliated institution which is paying any applicable article publishing charge ("APC") for Open Access articles. Where the Submitting Author wishes to make the Work available on an Open Access basis (and intends to pay the relevant APC), the terms of reuse of such Open Access shall be governed by a Creative Commons licence – details of these licences and which Creative Commons licence will apply to this Work are set out in our licence referred to above.

Other than as permitted in any relevant BMJ Author's Self Archiving Policies, I confirm this Work has not been accepted for publication elsewhere, is not being considered for publication elsewhere and does not duplicate material already published. I confirm all authors consent to publication of this Work and authorise the granting of this licence.

For peer review only

# Effects of Behavioral Parent Training for children with attention-deficit/hyperactivity disorder on Parenting Behavior: A protocol for an Individual Participant Data Meta-analysis

**Laura A. Steenhuis°**, University of Groningen, University Medical Center Groningen, Department of Child and Adolescent Psychiatry, Groningen, the Netherlands
**Annabeth P. Groenman°,** University of Groningen, University Medical Center Groningen, Department of Child and Adolescent Psychiatry, Groningen, the Netherlands
**Pieter J. Hoekstra,** University of Groningen, University Medical Center Groningen, Department of Child and Adolescent Psychiatry, Groningen, the Netherlands
**Rianne Hornstra,** University of Groningen, University Medical Center Groningen, Department of Child and Adolescent Psychiatry, Groningen, the Netherlands
**Marjolein Luman*,** Department of Clinical, Neuro, and Developmental Psychology, Vrije Universiteit Amsterdam, Amsterdam, The Netherlands. De Bascule, academic centre for child and adolescent psychiatry, The Netherlands.
**Saskia vd Oord*,** Department of Clinical Psychology, KU Leuven; Developmental Psychology, University of Amsterdam
**Barbara J. vd Hoofdakker*,** University of Groningen, University Medical Center Groningen, Department of Child and Adolescent Psychiatry, Groningen, the Netherlands; University of Groningen, Department of Clinical Psychology and Experimental Psychopathology, Groningen, The Netherlands

**° shared first author, authors made equal contributions to the project.**
* Shared last author, authors made equal contributions to the project.

**Corresponding Author:** Annabeth P. Groenman,

Address: Accare, Department of Child and Adolescent Psychiatry, Freepost 176, 9700 VB Groningen, the Netherlands

E-mail address: a.groenman@gmail.com

**Word count main body: 4385 [max 4000]**
**Word count abstract: 290 [max 300]**

For peer review only

**Acknowledgements**
We would like to thank all collaborators of the PAINT-IPDMA project for providing input to this protocol and for providing data for this project. We thank our parents of clients' expert panel for their valuable input during the conception of the idea for the PAINT-IPDMA.

**Exclusive License**
I, the Submitting Author have the right to grant and do grant on behalf of all authors of the Work (as defined in the below author licence), an exclusive licence and/or a non-exclusive licence for contributions from authors who are: i) UK Crown employees; ii) where BMJ has agreed a CC-BY licence shall apply, and/or iii) in accordance with the terms applicable for US Federal Government officers or employees acting as part of their official duties; on a worldwide, perpetual, irrevocable, royalty-free basis to BMJ Publishing Group Ltd ("BMJ") its licensees and where the relevant Journal is co-owned by BMJ to the co-owners of the Journal, to publish the Work in BMJ Open and any other BMJ products and to exploit all rights, as set out in our licence.

The Submitting Author accepts and understands that any supply made under these terms is made by BMJ to the Submitting Author unless you are acting as an employee on behalf of your employer or a postgraduate student of an affiliated institution which is paying any applicable article publishing charge ("APC") for Open Access articles. Where the Submitting Author wishes to make the Work available on an Open Access basis (and intends to pay the relevant APC), the terms of reuse of such Open Access shall be governed by a Creative Commons licence – details of these licences and which Creative Commons licence will apply to this Work are set out in our licence referred to above.

For peer review only

**Abstract**

Introduction: Behavioral Parent Training (BPT) is a well-established treatment for children with attention-deficit/hyperactivity disorder (ADHD). BPT is based on the hypothesis that improvements in parenting are mediators of improvements in children's behaviors. However, meta-analyses show considerate heterogeneity in effects of BPT on child outcomes, and meta-analyses on parenting outcomes are scarce. Also, few studies have investigated parenting factors as mediators of child outcomes. This study aims to examine the effects and moderators of BPT on parenting outcomes and whether improvements in parenting mediate amelioration of behavior and impairment in children with ADHD.

Methods and analyses: We will conduct an individual participant data meta-analysis (IPDMA), making use of individual data of existing trials, and giving the opportunity for highly powered moderator analyses. This IPDMA will be performed by the Psychosocial ADHD INTervention (PAINT) collaboration. We will include randomized controlled trials of BPT, for individuals with ADHD below 18 years old. Systematic searches have been performed to locate relevant papers. Authors are currently contacted to share their data with the PAINT-IPDMA project. We will examine effects of BPT on parenting outcomes (e.g., positive and negative parenting, management of affect, perceived parenting competence, parenting stress), moderators of these effects (e.g., parental depression, parenting stress, severity of the child's ADHD symptoms) and subsequently perform mediation analyses where parenting outcomes are modelled as mediators of child outcomes (e.g., symptoms and severity of ADHD, comorbid psychopathology, and impairment).

Ethics and Dissemination: We will include data from RCTs for which ethical approval has been received and consent forms have been signed. De-identified data will be provided by the original investigators. We aim to disseminate our findings through peer-reviewed scientific journals, presentations at (inter)national scientific meetings, newsletters, the website of our project and the Dutch academic workspace ADHD.

For peer review only



For peer review only - http://bmjopen.bmj.com/site/about/guidelines.xhtml

Article summary

*Strengths and limitations of this study:*

- To our knowledge this is the first IPDMA that examines the effects of behavioral parent training for ADHD on parenting outcomes.
- By using IPDMA, we are able to conduct highly powered moderation analysis.
- By collecting individual participant data from randomized controlled trials, we have the opportunity to perform uniform analyses across studies.
- Our IPDMA includes only variables that are reported consistently across the majority of studies in the database.
- Representativeness of the database will depend partly on the willingness and ability of investigators to share their data.

For peer review only



For peer review only - http://bmjopen.bmj.com/site/about/guidelines.xhtml

**Introduction**

Behavioral parent training (BPT) is a well-established intervention for children with attention-deficit/hyperactivity disorder (ADHD) and recommended as first line treatment by many international guidelines [1–3]. In BPT, parents are trained to apply behavioral techniques meant to increase prosocial and adaptive child behaviors and to reduce disruptive and maladaptive child behaviors. In addition, BPT focusses on nurturing and positive parent-child relationships [4]. Several meta-analyses on BPT for children with ADHD have shown medium to large effect sizes on parent reported reductions of ADHD symptoms, comorbid problems, impairment and parenting behaviors [3,5–7]. However, the full range of relevant parenting outcomes of BPT has not been assessed in meta-analyses [7,8]. Moreover, while improvements in parenting behaviors are thought to mediate improvements in children's behaviors and associated impairments [3,9], studies investigating these mediation effects are scarce. Another unresolved issue is that individual studies commonly lack the statistical power to adequately assess moderators of improvements of parenting outcomes. Knowing which moderators impact the effectiveness of BPT for child [5] and parenting outcomes [3,6], in addition to knowing which mediators drive the effects of BPT will give more insight into for whom BPT works best and into the working mechanisms underlying this, therefore contributing to personalized treatment of children with ADHD.

BPT aims to increase the demonstration of positive parenting behaviors and to inhibit negative parenting behaviors, improve the management of affect, reduce parenting stress and enhance parenting self-efficacy [9], in order to positively influence children's behaviors and decrease impairment. Parents are trained to modify environmental antecedents (e.g., providing structure) and consequences (e.g., positive rewards for adaptive behavior) of behavior. Positive parenting behaviors consist of providing praise, encouragement, effective communication, demonstrating positive affect and offering physical affirmations [10,11], whereas negative parenting behaviors are described as providing inconsistent discipline, verbal criticism, corporal punishment, poor supervision, and demonstrating negative affect [12–14]. Essential to positive parenting behavior is the effective management of parental affect, which is often a specific target of BPT [15]. Parents are taught to express positive emotions (e.g., love, affection and warmth) and to inhibit expressions of negative and unsupportive emotions (e.g., anger, frustration and irritability) [16]. Parenting stress (i.e. stress arising from the feeling that the demands of parenting outweigh the resources [17]) is often reported by parents of children with ADHD [18] and is also an important target of BPT. Lastly, parenting self-efficacy is an important target of BPT [19]; parents should feel more confident and competent in carrying out their parenting tasks.



For peer review only - http://bmjopen.bmj.com/site/about/guidelines.xhtml

When assessing parenting outcomes of BPT, it is important to consider a range of outcomes, particularly positive and negative parenting behaviors, management of affect, parenting stress and parenting self-efficacy. However, treatment trials of BPT have not uniformly assessed all parenting outcomes that are explicit targets of BPT [3,6], although BPT outcomes may differ for these different domains. For example, a recent systematic review investigated multiple parenting outcomes (display of parental affect, parenting stress, and parenting self-efficacy) of BPT and showed positive results for outcomes most closest to the target of BPT (e.g., parenting self-efficacy), but less so for more distal outcomes (e.g., parental psychopathology) [20]. Notably, studies in that review were not specifically conducted in ADHD samples, nor were outcomes quantified using a (individual participant data) meta-analytic approach, which will be the analytic approach in the current study. Recently, two meta-analyses examined the effectiveness of behavioral interventions (mostly consisting of BPT) on parenting outcomes in samples of children with ADHD, either assessed by raters unblinded to the treatment condition (e.g., parents involved in BPT [9]) or by blinded raters (e.g., independent raters coding video-taped interactions between the parent and child [3,6]). The first meta-analysis [3] considered effects on positive and negative parenting behaviors and parenting self-efficacy, immediately post-intervention. Results indicated improvements in positive parenting behaviors (medium effect sizes (ES) of 0.68 and 0.63 for unblinded and blinded raters, respectively), reductions in negative parenting behaviors (medium to small ES of 0.57 and 0.43 for unblinded and for blinded raters, respectively), and improvements in parenting self-efficacy (small ES of 0.37 for unblinded raters). The other meta-analysis [6] investigated the effects of BPT for preschool children with (or at risk for) ADHD, and reported medium to small ES of 0.63 and 0.33 by unblinded and blinded raters, respectively, for reductions in negative parenting behaviors. In addition, long-term effects (up to 12 months) on negative parenting behaviors were reported by unblinded raters with an ES of 0.12 (very small). Overall, both unblinded and blinded parent outcomes show improvements after BPT, although the effects on blinded outcomes generally appeared somewhat smaller than the effects on unblinded outcomes. Improvements on other relevant domains such as parenting stress and display of parental affect have not been addressed in meta-analyses, although individual studies demonstrated that parenting stress can be effectively alleviated with BPT [19], and that mothers who participated in BPT have less negative affect and better emotion regulation abilities post-treatment [21].

Heterogeneity in effect sizes is common in meta-analyses investigating parenting outcomes of BPT for children with ADHD [3,6]. More knowledge on factors associated with treatment effects will yield more insight into for whom BPT works best, and may allow clinicians to make better treatment choices tailored to individuals. So far, very few randomized control trials (RCTs) were adequately powered for moderator analyses, and existing moderator analyses were mostly limited to



For peer review only - http://bmjopen.bmj.com/site/about/guidelines.xhtml

child behavioral outcomes [22–24]. While traditional methods of addressing heterogeneity in meta-analyses (such as excluding extreme ES, subgroup analyses, or meta-regression) may resolve heterogeneity (i.e. reduce $I^2$), they reveal little about the cause of this heterogeneity. Individual participant data meta-analysis (IPDMA) includes data at an individual level rather than at study level, which enables exploration of moderators, therefore yielding more information about the cause of heterogeneity. Moreover, IPDMA performs a uniform analysis across all studies. IPDMA also has enough power to perform subgroup analyses, which most individual RCTs lack. Due to the collaborative nature of an IPDMA, collaborators can provide input on all phases of the research (including design, analyses, interpretation and manuscript preparation), leading to a high quality product [25–27]. So far, no IPDMA has been conducted for parenting outcomes of BPT for children with ADHD.

In this paper, we present our protocol for an IPDMA on BPT for children with ADHD. The current IPDMA will explore several child- and parent-related moderators on parenting outcomes of BPT. Given that the examination of moderators on parenting outcomes is scarce, the choice of possible moderators to be investigated in this IPDMA will also be drawn from BPT trials on child outcomes. First, age of the child might be an important moderator [28], as parents may have more influence on younger children, and younger children may have less engrained symptoms. A meta-analysis indeed demonstrated that BPT had more effect on positive parenting behaviors for younger compared to older children with ADHD [3]. Second, medication use of the child may moderate BPT outcomes, as parents of children on medication might find it easier to adapt their parenting styles and experience less resistance when doing so, although there could also be a floor effect as that children with medication might already function better. Previous studies suggested that medication use may positively contribute to BPT outcomes on child's ADHD symptoms [29], although results have been mixed [22]. Third, it seems plausible that intelligence of the child is positively associated with a treatment that involves learning associations between behaviors and consequences and new skills [30]. There is some evidence that higher children's IQ is associated with more improvement in ADHD symptoms following behavioral treatment, but only for specific subgroups of children, such as girls with more anti-social symptoms [24,31,32]. Fourth, pretreatment ADHD severity and presence of comorbidities could moderate BPT, as parents might find it easier to change their parenting behavior when the child has less complex symptomatology. Previous individual studies have indeed confirmed that comorbidities at baseline negatively impacted the outcome of BPT treatment with regard to child symptoms [22,23,33].

There are several parent-related moderators that may have an effect on parenting outcomes of BPT. First, parental mental health problems (depression, ADHD, parental stress) are likely to affect how well parents are able to grasp new information and impact their ability to learn new methods of



For peer review only - http://bmjopen.bmj.com/site/about/guidelines.xhtml

parenting behaviors and skills [22,23,34–36]. Second, low socio-economic status (SES) may moderate outcomes of BPT, as this may increase family strain and impact the availability of family resources. There is mixed evidence of the moderating effect of low SES on the effects of BPT on ADHD symptoms and related problems, showing either no effect [24], or better outcomes for parents with lower SES [37]. So far, effects of low SES in relation to parenting outcomes of BPT for children with ADHD have not been investigated. For the present IPDMA we will rely on an imperfect measure of SES, i.e., parental education, as we are reliant on the measures that are consistently used across studies Third, single parenthood may also moderate outcomes of BPT, as individual studies have demonstrated that single mothers are less likely to respond to BPT [38,39], and maintain treatment gains over time [40] in terms of parenting behavior. Indeed, research suggests that single parents benefit more from enhanced BPT programs, suited to their specific needs (STEPP; [41,42]), compared to standard BPT programs. Fourth, there is evidence that lower parenting self-efficacy at baseline has a negative impact on improvements in behavioral problems in children with ADHD following BPT [43] and one could expect similar results for parenting outcomes. On the other hand, it is also possible that lower parenting abilities at baseline yields more room for improvement in BPT, and thus both directions of the effect could be expected.

For peer review only

Following the investigation of parenting outcomes of BPT as part of our IPDMA, the next step will be to examine mediators of improvements in child's behavior following BPT. Currently, there are no meta-analyses analyzing whether improvements in parenting behavior mediate improvements of BPT on symptoms of ADHD of the child. Some evidence of individual studies shows that reductions in parenting behaviors or parenting attributions mediate improvements on child outcomes in behavioral interventions [12,35,44]. This IPDMA aims to synthesize the available data regarding the association between improvements in parent outcomes and improvements in child's outcomes following BPT.

The specific aims of our IPDMA include:

(1) to investigate effects of BPT on parenting outcomes (positive and negative parenting behaviors, display of parental affect, parenting stress and parenting self-efficacy). Given that BPT for ADHD has shown different effects when assessed by unblinded raters and by more blinded raters [3,6,7], we aim to distinguish (if possible) between unblinded and blinded assessments of parenting behavior and display of parental affect;

(2) to investigate possible child (including age, medication use, IQ, ADHD severity, presence and severity of comorbidities) and parent (including depression, ADHD, socio-economic status, single parenthood, and parenting measures) moderators of parenting outcomes;

(3) to investigate whether improvements in parenting behaviors mediate the effect of BPT on behavioral child outcomes and impairment following treatment.



For peer review only - http://bmjopen.bmj.com/site/about/guidelines.xhtml

For aims 1-3, outcomes of BPT will be examined immediately post-treatment and at long-term where possible.

## Methods and Analysis

For this IPDMA we will build on the Psychosocial ADHD INTervention IPD (PAINT-IPD) database which is registered in prospero: https://www.crd.york.ac.uk/PROSPERO/display_record.php?ID=CRD42017069877&ID=CRD42017069877. In this project we collect data on psychosocial treatments for children with ADHD: the search is regularly updated and the database continues to expand. For the purpose of the current IPDMA on parenting outcomes, we will use the same methods and search strategy. This protocol is written in line with the PRISMA-P 2015 checklist [45] (see supplementary checklist). The current study is planned to commence September 2020.

### Inclusion criteria

We will include RCTs of behavioral treatments of individuals aged below 18 with ADHD, corroborated by clinical cutoffs on questionnaires or (semi)-structured interviews. We will include studies that compared BPT with a control condition (i.e., all conditions that are labeled control, including active treatment), and studies that compared BPT to another behavioral intervention (head to head comparisons). We excluded studies or intervention arms that used optimized medication treatment next to BPT as part of their study design or as a control condition. We define BPT as interventions directed at changing children's behaviors (i.e., increasing desirable behaviors and decreasing undesirable behaviors), using (cognitive) behavioral therapeutic techniques which parents are trained in [46]. Multimodal interventions (consisting of both parent and/or teacher and/or child training), will be included if the time spent on parent training within the intervention was at least equal to other types of training.

### Selection and screening of studies

The last systematic search was performed on August 1st 2019. Currently, we are contacting the authors of the newly identified studies. Two authors (AG and RH) performed the selection and screening of studies, disagreement was resolved by consensus. A two-step approach to identifying relevant articles was used. First, Medline, CINAHL, PsycINFO, EMBASE+EMBASE CLASSIC, ERIC, Web of Science [Science Citation Index Expanded] was searched for relevant papers using a combination of the following search terms and their synonyms, as well as hierarchical family form (e.g., MeSH terms): treatment specific terms (e.g., behavioral treatment, psychosocial treatment, and parent



For peer review only - http://bmjopen.bmj.com/site/about/guidelines.xhtml

training), ADHD, child, and randomized controlled trial. No date restrictions were applied. English, German and Dutch language publications published in peer-reviewed journals were included. Second, literature lists of all selected studies and relevant systematic reviews and meta-analyses were hand-searched to identify possible missing articles (complete search criteria for each database are available in Supplement 1).

**Data collection and management**

Author contact

We will contact the corresponding authors of all eligible trials to ask for their participation in the current IPD meta-analyses. If after several weeks we have had no response, we will send a reminder to the corresponding author. If we have failed to establish contact with the corresponding author, we will email the other authors of the study. Furthermore, we will contact researchers during conferences, and through our personal network to retrieve all eligible databases.

Data format and management

Part of the data has already been received in light of our previous IPDMA [5]. For newly identified data that we have not yet received, we will use the best, safest way to transfer the data. The most convenient way for most authors will be to transfer the encrypted data per email to the project management, but we will be open to other options (e.g., face to face transfer). A copy of our data collection manual for the PAINT-IPD database can be found in Supplement 2. We will allow authors to send the data in all possible formats, although the most preferable format would be one in which each subject represents a row and each variable a column.

If authors are not included in the PAINT collaborators group yet [5], we will offer one or two authors of each included study a place in our PAINT collaborators group. We plan yearly telephone calls and/or meetings at large international conferences to keep the working group up to date and discuss design and methodological issues. Members of the working group who have provided data on BPT trials with parenting outcomes will have the opportunity to provide feedback on the first draft of the manuscript and will be sent a copy of the final manuscript before submission. Only authors in the IPD steering committee group (APG, BvdH, SvdO, ML and PH) will have access to the data.

Ethics and Dissemination

The original investigators will be asked for de-identified data, so that only the original investigator knows the link between data and participant. We will only include RCTs where ethical approval has been given and participants signed consent forms. Since the current IPDMA is an extension of the original purpose of the eligible studies, we do not expect any ethical issues with the



For peer review only - http://bmjopen.bmj.com/site/about/guidelines.xhtml

current IPDMA. Results of our study will be disseminated through peer-reviewed scientific journals, and presentations on (inter)national scientific and/or clinical expert meetings.

Patient and Public Involvement

An expert panel consisting of parents of children with ADHD was organized at the conception of the idea in which feedback was given to the plans and changes were made accordingly. Our results will be communicated to clinicians, clients and their parents through newsletters, and through the academic workplace "ADHD en druk gedrag" (also see https://adhdendrukgedrag.nl/) in which many parents, client organizations, and mental health care professionals are represented.

Variables

For the PAINT-IPD database, a data request form will be sent to all authors of the original studies, containing a list of variables that will be requested (see Supplement 2a). This list was determined by reviewing the literature and the IPDMA steering committee (authors APG, SvdO, ML, PH and BvdH) assessed these outcomes domains for suitability and interest. The final list of variables will depend on the available data of all studies.

For the current protocol, the following parenting variables will be selected:

*Positive and negative parenting behaviors* will be assessed using unblinded and blinded measures. For unblinded parenting measures, we will select questionnaires such as the Alabama Parenting Questionnaire [10]. For blinded measures, we will select assessments used to observe and code parent behavior, such as the Dyadic Parent-Child Interaction Coding System [47].

*Display of Positive and negative parental affect* will be assessed with measures specifically designed to capture parental affect (e.g., 5 min speech sample of expressed emotion; [15]) or a subscale of an existing parenting scale (e.g., attachment domain of the Parenting-Relationship Questionnaire [48]).

*Parental stress* will be assessed using measures specifically designed to capture stress from parenting, such as the Parenting Stress Index (PSI; [49]), but also measures assessing stress in the caregiver more generally (Depression and Anxiety Stress Scales, DASS; [50]).

*Parenting self-efficacy* will be assessed using measures specifically designed to capture parenting self-efficacy, such as the Parental Sense of Competence Scale (PSOC; [51]), a subscale of an existing parenting scale (e.g., the Parenting-Relationship Questionnaire, [48]), or a scale designed to assess parenting competence in specific contexts, such as education (e.g., Parent as Educator Scale, [52]).

*Parental depression* will be assessed using measures to capture depressive symptoms, such as the Beck Depression Inventory (BDI; [53]), a subscale of an existing mental health questionnaire



For peer review only - http://bmjopen.bmj.com/site/about/guidelines.xhtml

For peer review only

(e.g., depression subscale of the Depression and Anxiety Stress Scales, DASS; [54]) or a mental health questionnaire for which the overall score can be used as a proxy for depression (e.g., the General Health Questionnaire, GHQ; [55]).

*Parental ADHD* will be assessed using an adult measure of ADHD, such as the Adult Self-Report Scale (ASRS) Screener [56].

The remaining parent variables, including *single parenthood* and *socio-economic status (SES),* are commonly assessed with demographic questionnaires or items. SES can be assessed in different ways (e.g. based on income, occupation, neighborhood or education) and due to our IPDMA methodology we are reliant on choosing the assessment measure that is most consistently used across studies. Given that data on parental education is often available, and is deemed acceptable as a proxy for SES [5,57], parental education level will serve as a proxy measure for SES in the current study.SES will be conceptualized as low (<high school), medium (high school graduate or college education), or high (>college graduate).

Note that *parenting behaviors, parental affect, parental stress* and *parenting self-efficacy* will serve as moderators (to predict parenting outcomes), mediators (in the relationship between BPT and child outcomes) and outcome variables (in the moderator analysis). *Parental depression, parental ADHD, single parenthood and socio-economic status* will serve as baseline moderators to predict parenting outcomes.

Additionally the following child variables will be selected:

*Child ADHD severity* will be assessed using a parent-rated measure of childhood ADHD, such as the ADHD subscale of the Connors Parent Rating Scale [57].

*Comorbidity* will be assessed using symptoms of Oppositional Defiant Disorder (ODD) and/or Conduct Disorder (CD), by for example the ODD subscale of the Connors Parent Rating Scale [57], or the CD subscale of the Disruptive Behavior Disorder Rating Scale [58]. In addition, internalizing symptoms will be assessed, using for example the internalizing subscale of the Child Behavior Checklist [59].

*Global impairment* will be assessed using a parent- or clinician-rated measure of global impairment, such as the Impairment Rating Scale [60].

The remaining child variables, including *child age, medication use and IQ* are commonly assessed with demographic questionnaires or items.

*Child ADHD severity, comorbidity and global impairment* will serve as moderators (to predict parenting outcomes) and outcome variables (in the mediation analysis). *Child age, medication use and IQ* will solely serve as moderators to predict parenting outcomes.



For peer review only - http://bmjopen.bmj.com/site/about/guidelines.xhtml

For all variables (moderators and outcomes), when a study reports multiple measures to capture the same concept, the measure which is most often used by other studies will be included. If multiple raters (mothers and fathers) are included to assess the same concept, the assessment by the mother will be preferred as they are more often the primary caregiver and more often take part in BPT. Regarding harmonization, for each dataset continuous measures will be converted into z-scores, using pre-intervention-score standard deviations within studies.

Quality assessment

Quality assessment will be done independently by three authors (a combination of AG, RH and LAS) using Cochrane risk of bias. Any disagreement will be resolved by consensus. Once the data has been received, all raw data sets will be checked for impossible, missing, or extreme values. We will collect data on all randomized participants, and subsequently adopt the intention to treat principle. As this will possibly reintroduce participants who were previously excluded, we will also check randomization parameters (e.g., age, gender, and ADHD severity of the participants). If any unexpected deviations are found between our results and the published results, the original researcher will be contacted to locate the origin of this deviation.

Analysis

Effects of BPT will be calculated using a one-stage IPDMA, in which data from participants across studies will be analyzed in one stage, clustered by study. A linear multilevel analysis will be used to examine the effects of BPT on parenting outcome measures. A random intercept for study will be added to each model. Post intervention outcome measures will be used as dependent variables in these models, and pre-intervention outcome measures and intervention group will be added. The interaction between intervention group and moderators of interest will be added to the model to assess their moderating effect on treatment outcomes. To analyze changes in parenting behaviors as a potential mediator in the relationship between BPT and child outcomes, the change in parenting behaviors (post-treatment score – baseline score) will be used. An interaction between intervention group and change in parenting behaviors will be added to the model to determine whether change in parenting behaviors had a main effect on child outcomes and/or an interactive effective with treatment [61,62]. We will conduct sensitivity analyses between studies that provided data and those that did not, for demographic characteristics (e.g., age), and for inclusion criteria (cut-off on measures versus meeting diagnostic criteria for ADHD) and reported effect sizes. If sufficient data is available, a subgroup analysis for child age will be conducted, where all analyses will be repeated separately for pre-school children (<6 years of age), school aged children (6-12 years of age) and adolescents (>12 years of age).



For peer review only - http://bmjopen.bmj.com/site/about/guidelines.xhtml

## Discussion

The current protocol presents the first IPDMA to synthesize research findings on treatment effects of BPT for children with ADHD on parenting outcomes. Both child and parent moderators of parenting outcomes will be explored and a mediation analysis will be conducted to examine whether changes in parenting behaviors mediate the effect of BPT on child behaviors and impairment. Previous efforts to identify moderators of parenting outcomes are scarce, as there are few well powered RCTs and only two meta-analyses on this topic [3,6]. The heterogeneity found in parenting outcomes in previous meta-analyses suggest that there is not a "one size fits all" solution of BPT for children with ADHD. Parents and children who differ with regard to their personal, clinical and demographic characteristics, are also likely to differ in their response to BPT. Clearly, an IPDMA approach is needed to further examine the effects of BPT on parenting outcomes, to elucidate potential sources of heterogeneity amongst children and parents, and to investigate potential mechanisms of change in BPT for children with ADHD. Ultimately, both research and clinical practice may be informed by the knowledge of which child and/or parent responds best to a certain treatment, thereby contributing to the overall goal of providing the best care to children with ADHD and their parents.

An additional important aim of the current IPDMA will be to examine a crucial assumption about the working mechanism of BPT, namely whether improvements in parenting behaviors mediate improvements in child behavior and impairment. So far, single studies have examined this assumption, finding some supportive evidence [12,19], but this hypothesis has never been addressed in a meta-analysis, let alone by using an IPDMA approach. By synthesizing all available (raw) data from RCTs, we will conduct highly sophisticated and powered statistical analyses, to provide insight into the working mechanisms of BPT. This knowledge will allow us to further improve and refine BPT programs for children with ADHD.

Despite clear strengths of the IPDMA approach, such as high-powered moderator, mediation and subgroup analyses, there are some limitations as well [25,27,63]. First, IPDMA cannot change anything about the way the study was originally conducted. Second, it is unclear whether enough studies measured the variables of interest, and thus whether all intended outcomes can be examined. Third, if investigators of trials are unwilling to share their data (or may not be able to share their data), not all relevant data can be included in the synthesis. Fourth, not all factors which may affect study outcomes can be conceptualized as a moderator, such as efficacy versus effectiveness trials (i.e., studies often do not fit exactly into one of both categories). Fifth, since parental education levels are easy to collect, they are omnipresent in the data. Ideally several aspects



For peer review only - http://bmjopen.bmj.com/site/about/guidelines.xhtml

of SES are taken into account, such as income, occupation, and/or neighborhood [64,65], but this data is available in only few datasets.

Notwithstanding these limitations, this study has the potential to elucidate clinically relevant questions concerning the efficacy of BPT for children with ADHD and their parents and to provide insight in moderators and mediators of treatment effects. This knowledge may improve and optimize current treatment programs and could eventually lead to advances in personalized treatment for children with ADHD.

**Contributorship statement**

BvdH is the guarantor. APG wrote the first draft of the manuscript, and LAS wrote a revised and final version. APG, RH and LAS are performing the systematic search and data extraction. PJH, ML, SvdO, BvdH acquired funding for the PAINT-IPDMA project; an international database including individual participant data from RCTs examining psychosocial interventions for the treatment of ADHD in children. The current study is a part of the PAINT-IPDMA project. PJH, ML, SvdO, BvdH, and APG setup the initial design for the IPDMA. All authors worked together to design this IPDMA, contributed to the intellectual content of the current manuscript and approved the final version.

**Competing interests**

None declared.

**Funding**

This research was funded by The Dutch Organization for Health Research and Development (ZonMw) under grant number 729300013. The funder had no role in the design of this protocol. The funder will have no input on the collection of data, the data analysis or the interpretation or publication of the study results.

**Data sharing statement**

No data is available, as this is a study protocol.

**References**

1 Wolraich M, Brown L, Brown RT, *et al.* ADHD: Clinical practice guideline for the diagnosis, evaluation, and treatment of attention-deficit/ hyperactivity disorder in children and adolescents. Pediatrics. 2011. doi:10.1542/peds.2011-2654

2 NICE NI for H and CE. Attention deficit hyperactivity disorder: diagnosis and management (NICE Guideline 87). Natl. Inst. Heal. Care Excell. 2018.



For peer review only - http://bmjopen.bmj.com/site/about/guidelines.xhtml

3 Daley D, Van Der Oord S, Ferrin M, *et al.* Behavioral interventions in attention-deficit/hyperactivity disorder: A meta-analysis of randomized controlled trials across multiple outcome domains. J. Am. Acad. Child Adolesc. Psychiatry. 2014. doi:10.1016/j.jaac.2014.05.013

4 Kazdin AE. Parent management training: Evidence, outcomes, and issues. *J Am Acad Child Adolesc Psychiatry* Published Online First: 1997. doi:10.1097/00004583-199710000-00016

5 Groenman AP, Hornstra R, Hoekstra PJ, *et al.* Behavioral treatments for children and adolescents with attention-deficit/hyperactivity disorder: an individual participant data meta-analysis. *Submitted*

6 Rimestad ML, Lambek R, Zacher Christiansen H, *et al.* Short- and Long-Term Effects of Parent Training for Preschool Children With or at Risk of ADHD: A Systematic Review and Meta-Analysis. J. Atten. Disord. 2019. doi:10.1177/1087054716648775

7 Sonuga-Barke EJ, Brandeis D, Cortese S, *et al.* Nonpharmacological interventions for ADHD: Systematic review and meta-analyses of randomized controlled trials of dietary and psychological treatments. Am. J. Psychiatry. 2013. doi:10.1176/appi.ajp.2012.12070991

8 Mulqueen JM, Bartley CA, Bloch MH. Meta-Analysis: Parental Interventions for Preschool ADHD. *J Atten Disord* Published Online First: 2015. doi:10.1177/1087054713504135

9 Daley D, Van Der Oord S, Ferrin M, *et al.* Practitioner review: Current best practice in the use of parent training and other behavioural interventions in the treatment of children and adolescents with attention deficit hyperactivity disorder. *J Child Psychol Psychiatry Allied Discip* Published Online First: 2017. doi:10.1111/jcpp.12825

10 Frick P. The Alabama Parenting Questionnaire. *Unpubl Rat scale, Univ Alabama* 1991.

11 Morris AS, Silk JS, Steinberg L, *et al.* Temperamental vulnerability and negative parenting as interacting predictors of child adjustment. J. Marriage Fam. 2002. doi:10.1111/j.1741-3737.2002.00461.x

12 Hinshaw SP, Owens EB, Wells KC, *et al.* Family processes and treatment outcome in the MTA: Negative/ineffective parenting practices in relation to multimodal treatment. *J Abnorm Child Psychol* 2000;**28**:555–68. doi:10.1023/A:1005183115230

13 Sanders MR. Triple P-Positive Parenting Program as a Public Health Approach to Strengthening Parenting. *J Fam Psychol* Published Online First: 2008. doi:10.1037/0893-3200.22.3.506

14 Wells KC, Epstein JN, Hinshaw SP, *et al.* Parenting and Family stress treatment outcomes in attention deficit hyperactivity disorder. *J Abnorm Child Psychol* 2000;**6**:543–53.https://link.springer.com/content/pdf/10.1023%2FA%3A1005131131159.pdf

15 Daley D, Sonuga-Barke EJS, Thompson M. Assessing expressed emotion in mothers of

For peer review only

preschool AD/HD children: Psychometric properties of a modified speech sample. *Br J Clin Psychol* Published Online First: 2003. doi:10.1348/014466503762842011

16 Herbert SD, Harvey EA, Roberts JL, *et al.* A Randomized Controlled Trial of a Parent Training and Emotion Socialization Program for Families of Hyperactive Preschool-Aged Children. *Behav Ther* 2013;**44**:302–16. doi:10.1016/j.beth.2012.10.004

17 Deater-Deckard K, Smith J, Ivy L, *et al.* Differential perceptions of and feelings about sibling children: Implications for research on parenting stress. Infant Child Dev. 2005. doi:10.1002/icd.389

18 Johnson JH, Reader SK. Assessing stress in families of children with ADHD: Preliminary development of the disruptive behavior stress inventory (DBSI). *J Clin Psychol Med Settings* Published Online First: 2002. doi:10.1023/A:1014136029697

19 Heath CL, Curtis DF, Fan W, *et al.* The Association Between Parenting Stress, Parenting Self-Efficacy, and the Clinical Significance of Child ADHD Symptom Change Following Behavior Therapy. *Child Psychiatry Hum Dev* 2014;**46**:118–29. doi:10.1007/s10578-014-0458-2

20 Colalillo S, Johnston C. Parenting Cognition and Affective Outcomes Following Parent Management Training: A Systematic Review. Clin. Child Fam. Psychol. Rev. 2016. doi:10.1007/s10567-016-0208-z

21 Gershy N, Meehan KB, Omer H, *et al.* Randomized Clinical Trial of Mindfulness Skills Augmentation in Parent Training. *Child Youth Care Forum* 2017;**46**:783–803. doi:10.1007/s10566-017-9411-4

22 Van Den Hoofdakker BJ, Nauta MH, Veen-Mulders L Van Der, *et al.* Behavioral parent training as an adjunct to routine care in children with attention-deficit/hyperactivity disorder: Moderators of treatment response. *J Pediatr Psychol* Published Online First: 2010. doi:10.1093/jpepsy/jsp060

23 Veenman B, Luman M, Oosterlaan J. Moderators influencing the effectiveness of a behavioral teacher program. *Front Psychol* Published Online First: 2018. doi:10.3389/fpsyg.2018.00298

24 Owens EB, Hinshaw SP, Arnold LE, *et al.* Which treatment for whom for ADHD? Moderators of treatment response in the MTA. J. Consult. Clin. Psychol. 2003. doi:10.1037/0022-006X.71.3.540

25 Riley RD, Lambert PC, Abo-Zaid G. Meta-analysis of individual participant data: Rationale, conduct, and reporting. *BMJ* Published Online First: 2010. doi:10.1136/bmj.c221

26 Higgins JPT, Jackson D, Barrett JK, *et al.* Consistency and inconsistency in network meta-analysis: concepts and models for multi-arm studies. *Res Synth Methods* 2012;**3**:98–110. doi:10.1002/jrsm.1044

27 Stewart LA, Tierney JF. To IPD or not to IPD? Advantages and disadvantages of systematic



For peer review only - http://bmjopen.bmj.com/site/about/guidelines.xhtml

For peer review only

reviews using individual patient data. Eval. Heal. Prof. 2002. doi:10.1177/016327870202500100 6

28 Owens EB, Hinshaw SP, McBurnett K, *et al.* Predictors of Response to Behavioral Treatments Among Children With ADHD-Inattentive Type. *J Clin Child Adolesc Psychol* Published Online First: 2018. doi:10.1080/15374416.2016.1228461

29 Jensen, Arnold, Richters, Severe, Vereen V al. (1999). Moderators and mediators of treatment response for children with attention-deficit/hyperactivity disorder: The multimodal treatment study of children with attention-deficit/hyperactivity disorder. *Arch Gen Psychiatry* Published Online First: 1999. doi:10.1001/archpsyc.56.12.1088

30 Hallahan DP. Cognitive-behavioral therapy for impulsive children. *Child Youth Serv Rev* Published Online First: 1986. doi:10.1016/0190-7409(86)90045-9

31 Kazdin AE, Crowley MJ. Moderators of treatment outcome in cognitively based treatment of antisocial children. *Cognit Ther Res* Published Online First: 1997. doi:10.1023/A:1021832729480

32 Van der Oord S, Prins PJM, Oosterlaan J, *et al.* Efficacy of methylphenidate, psychosocial treatments and their combination in school-aged children with ADHD: A meta-analysis. Clin. Psychol. Rev. 2008. doi:10.1016/j.cpr.2007.10.007

33 Lee SS, Falk AE, Aguirre VP. Association of comorbid anxiety with social functioning in school-age children with and without attention-deficit/hyperactivity disorder (ADHD). *Psychiatry Res* Published Online First: 2012. doi:10.1016/j.psychres.2012.01.018

34 Langberg JM, Evans SW, Schultz BK, *et al.* Trajectories and Predictors of Response to the Challenging Horizons Program for Adolescents With ADHD. *Behav Ther* Published Online First: 2016. doi:10.1016/j.beth.2016.01.001

35 Chronis-Tuscano A, O'Brien KA, Johnston C, *et al.* The relation between maternal ADHD symptoms & improvement in child behavior following brief behavioral parent training is mediated by change in negative parenting. *J Abnorm Child Psychol* 2011;**39**:1047–57. doi:10.1007/s10802-011-9518-2

36 Sonuga-Barke EJS, Daley D, Thompson M. Does Maternal ADHD Reduce the Effectiveness of Parent Training for Preschool Children's ADHD? *J Am Acad Child Adolesc Psychiatry* Published Online First: 2002. doi:10.1097/00004583-200206000-00009

37 Farmer CA, Brown N V., Gadow KD, *et al.* Comorbid Symptomatology Moderates Response to Risperidone, Stimulant, and Parent Training in Children with Severe Aggression, Disruptive Behavior Disorder, and Attention-Deficit/Hyperactivity Disorder. *J Child Adolesc Psychopharmacol* Published Online First: 2015. doi:10.1089/cap.2014.0109

38 Dumas JE, Wahler RG. Predictors of treatment outcome in parent training: Mother insularity



For peer review only - http://bmjopen.bmj.com/site/about/guidelines.xhtml

and socioeconomic disadvantage. *Behav Assess* 1983.

39 Webster-Stratton C, Hammond M. Predictors of treatment outcome in parent training for families with conduct problem children. *Behav Ther* Published Online First: 1990. doi:10.1016/S0005-7894(05)80334-X

40 Webster-Stratton C. Predictors of treatment outcome in parent training for conduct disordered children. *Behav Ther* Published Online First: 1985. doi:10.1016/S0005-7894(85)80048-4

41 Chacko A, Wymbs BT, Wymbs FA, *et al.* Enhancing traditional behavioral parent training for single mothers of children with ADHD. *J Clin Child Adolesc Psychol* Published Online First: 2009. doi:10.1080/15374410802698388

42 Chacko A, Uderman JZ, Zwilling A. Lessons Learned in Enhancing Behavioral Parent Training for High-Risk Families of Youth with ADHD. *ADHD Rep* Published Online First: 2013. doi:10.1521/adhd.2013.21.4.6

43 van den Hoofdakker BJ, Hoekstra PJ, van der Veen-Mulders L, *et al.* Paternal influences on treatment outcome of behavioral parent training in children with attention-deficit/hyperactivity disorder. *Eur Child Adolesc Psychiatry* 2014;**23**:1071–9. doi:10.1007/s00787-014-0557-4

44 Booster GD, Mautone JA, Nissley-Tsiopinis J, *et al.* Reductions in negative parenting practices mediate the effect of a family-school intervention for children with attention deficit hyperactivity disorder. *School Psych Rev* Published Online First: 2016. doi:10.17105/SPR45-2.192-208

45 Shamseer L, Moher D, Clarke M, *et al.* Preferred reporting items for systematic review and meta-analysis protocols (prisma-p) 2015: Elaboration and explanation. BMJ. 2015. doi:10.1136/bmj.g7647

46 Evans SW, Owens JS, Bunford N. Evidence-Based Psychosocial Treatments for Children and Adolescents with Attention-Deficit/Hyperactivity Disorder. *J Clin Child Adolesc Psychol* Published Online First: 2014. doi:10.1080/15374416.2013.850700

47 Eyberg SM, Nelson MMD, Duke M, *et al.* Manual for the Dyadic Parent-Child Interaction Coding System Third Edition. *System* 2004.

48 Rubinic D, Schwickrath H. Test Review: Kamphaus, R. W., & Reynolds, C. R. (2006). Parenting Relationship Questionnaire. Minneapolis, MN: NCS Pearson. J. Psychoeduc. Assess. 2010. doi:10.1177/0734282909346718

49 Abidin RR. Parenting Stress and the Utilization of Pediatric Services. *Child Heal Care* 1982;**11**:70–3. doi:10.1207/s15326888chc1102_5

50 Clara IP, Cox BJ, Enns MW. Confirmatory Factor Analysis of the Depression-Anxiety-Stress



For peer review only - http://bmjopen.bmj.com/site/about/guidelines.xhtml

Scales in Depressed and Anxious Patients. *J Psychopathol Behav Assess* 2001;**23**:61–7. doi:10.1023/A:1011095624717

51 Ohan JL, Leung DW, Johnston C. The parenting sense of competence scale: Evidence of a stable factor structure and validity. *Can J Behav Sci* Published Online First: 2000. doi:10.1037/h0087122

52 Mautone JA, Marcelle E, Tresco KE, *et al.* Assessing the quality of parent-teacher relationships for students with ADHD. *Psychol Sch* Published Online First: 2015. doi:10.1002/pits.21817

53 Beck AT, Steer RA, Carbin MG. Psychometric properties of the Beck Depression Inventory: Twenty-five years of evaluation. *Clin Psychol Rev* Published Online First: 1988. doi:10.1016/0272-7358(88)90050-5

54 Henry JD, Crawford JR. The short-form version of the Depression Anxiety Stress Scales (DASS-21): construct validity and normative data in a large non-clinical sample. *Br J Clin Psychol* 2005;**44**:227–39. doi:10.1348/014466505X29657

55 Goldberg DP, Hillier VF. A scaled version of the General Health Questionnaire. *Psychol Med* Published Online First: 1979. doi:10.1017/S0033291700021644

56 Kessler RC, Adler L, Ames M, *et al.* The World Health Organization adult ADHD self-report scale (ASRS): A short screening scale for use in the general population. *Psychol Med* Published Online First: 2005. doi:10.1017/S0033291704002892

57 Kao GS, Thomas HM. Test Review: C. Keith Conners Conners 3rd Edition Toronto, Ontario, Canada: Multi-Health Systems, 2008. *J Psychoeduc Assess* Published Online First: 2010. doi:10.1177/0734282909360011

58 Pelham Jr. WE, Gnagy EM, Greenslade KE, *et al.* Teacher ratings of DSM-III-R symptoms for the disruptive behavior disorders.[erratum appears in J Am Acad Child Adolesc Psychiatry 1992 Nov;31(6):1177]. *J Am Acad Child Adolesc Psychiatry* 1992.

59 Achenbach TM. *Manual for the Youth Self Report and 1991 Profile*. 1991.

60 Fabiano GA, Pelham WE, Waschbusch DA, *et al.* A practical measure of impairment: Psychometric properties of the impairment rating scale in samples of children with attention deficit hyperactivity disorder and two school-based samples. *J Clin Child Adolesc Psychol* Published Online First: 2006. doi:10.1207/s15374424jccp3503_3

61 HC K, GT W, CG F, *et al.* Mediators and moderators of treatment effects in randomized clinical trials. *Arch Gen Psychiatry* 2002.

62 Haasova M, Warren FC, Ussher M, *et al.* The acute effects of physical activity on cigarette cravings: Exploration of potential moderators, mediators and physical activity attributes using individual participant data (IPD) meta-analyses. Psychopharmacology (Berl). 2014. doi:10.1007/s00213-014-3450-4



For peer review only - http://bmjopen.bmj.com/site/about/guidelines.xhtml

63 Higgins JPT, Whitehead A, Turner RM, *et al.* Meta-analysis of continuous outcome data from individual patients. *Stat Med* Published Online First: 2001. doi:10.1002/sim.918

64 Shavers VL. Measurement of socioeconomic status in health disparities research. *J Natl Med Assoc* Published Online First: 2007. doi:10.13016/avw3-9cvx

65 Braveman PA, Cubbin C, Egerter S, *et al.* Socioeconomic status in health research: One size does not fit all. *J Am Med Assoc* Published Online First: 2005. doi:10.1001/jama.294.22.2879

For peer review only



For peer review only - http://bmjopen.bmj.com/site/about/guidelines.xhtml

Supplement 1- Search terms per database

**Behavioral interventions**

**PubMed**
("Attention Deficit Disorder with Hyperactivity"[Mesh] OR ADHD[tiab] OR ADD[tiab] OR attention deficit*[tiab] OR hyperactiv*[tiab] OR hyperkinetic*[tiab] OR minimal brain deficit*[tiab] OR minimal brain dysfunction*[tiab])
**AND**

(("Psychotherapy"[Mesh] OR psychotherap*[tiab] OR psychological therap*[tiab] OR psychological intervent*[tiab] OR psychoeduca*[tiab] OR mentoring*[tiab] OR coaching*[tiab] OR mindful*[tiab] OR relax*[tiab] OR meditat*[tiab])
OR
((parent*[tiab] OR mother[tiab] OR father[tiab] OR teacher*[tiab] OR school*[tiab])
*AND*
(program*[tiab] OR train*[tiab] OR educa*[tiab] OR therapy*[tiab] OR therapies*[tiab] OR therapeu*[tiab]OR intervention*[tiab] OR coaching*[tiab] OR counseling*[tiab]))
OR
((behavio*[tiab] OR cognit*[tiab] OR “acceptance and commitment”[tiab] OR dialectica*[tiab])
*AND*
(program*[tiab] OR therapy*[tiab] OR therapies*[tiab] OR therapeu*[tiab] OR intervention*[tiab] OR treatment*[tiab] OR train*[tiab]))
OR
(psychosocial[tiab]
AND
(treatment*[tiab] OR therapy*[tiab] OR therapies*[tiab] OR therapeu*[tiab] OR train*[tiab] OR intervention*[tiab] OR program*[tiab]))
OR
((skill*[tiab] OR organization*[tiab] OR organisation*[tiab] OR planning*[tiab] OR play*[tiab])
AND
(train*[tiab] OR intervention*[tiab] OR program*[tiab])))

**AND**

("Adolescent"[Mesh] OR "Child"[Mesh] OR child*[tiab] OR school*[tiab] OR infan*[tiab] OR adolescen*[tiab] OR pediatri*[tiab] OR paediatr*[tiab] OR boy[tiab] OR boys[tiab] OR boyhood[tiab] OR girl[tiab] OR girls[tiab] OR girlhood[tiab] OR youth[tiab] OR youths[tiab] OR teen[tiab] OR teens[tiab] OR teenage*[tiab] OR puberty[tiab] OR preschool*[tiab] OR toddler*[tiab] OR juvenile*[tiab] OR kids[tiab])
**AND**

("Controlled Clinical Trial" [Publication Type] OR "Randomized Controlled Trials as Topic"[Mesh] OR "Follow-Up Studies"[Mesh] OR follow-up[tiab] OR followup[tiab] OR control group*[tiab] OR "Random Allocation"[Mesh] OR random*[tiab] OR trial[ti])

For peer review only

**EMBASE EMBASE.com** (losse delen combineren op website)
('attention deficit disorder'/exp OR (ADHD OR ADD OR ‘attention deficit*’
OR hyperactiv* OR hyperkinetic* OR ‘minimal brain deficit*’ OR ‘minimal brain dysfunction*’):ab,ti)
**AND**

('psychotherapy'/exp OR (psychotherap* OR ‘psychological therap*’ OR ‘psychological intervent*’
OR psychoeduca* OR mentoring* OR coaching* OR mindful* OR relax*):ab,ti)
OR
((((parent* OR mother OR father OR teacher* OR school*)
*AND*
(program* OR train* OR educa* OR therapy* OR therapies* OR therapeu*OR intervention*
OR coaching* OR counseling*))
OR
((behavio* OR cognit* OR ‘acceptance and commitment’ OR dialectica*)
*AND*
(program* OR therapy* OR therapies* OR therapeu* OR intervention* OR treatment* OR
train*))
OR
(psychosocial
AND
(treatment* OR therapy* OR therapies* OR therapeu* OR train* OR intervention* OR
program*))
OR
((skill* OR organization* OR organisation* OR planning* OR play*)
AND
(train* OR intervention* OR program*))):ab,ti)

**AND**
('juvenile'/exp OR (child* OR school* OR infan* OR adolescen* OR pediatri* OR paediatr* OR boy OR
boys OR boyhood OR girl OR girls OR girlhood OR youth OR youths OR teen OR teens OR teenage*
OR puberty OR preschool* OR toddler* OR juvenile* OR kids):ab,ti)

**AND**
('controlled clinical trial'/exp OR 'randomization'/exp OR (‘control group*’ OR random*):ab,ti OR
trial:ti)

For peer review only

**EBSCO PsycINFO**

(DE "Attention Deficit Disorder" OR DE "Attention Deficit Disorder with Hyperactivity" OR ADHD OR ADD OR “attention deficit*” OR hyperactiv* OR hyperkinetic* OR “minimal brain deficit*” OR “minimal brain dysfunction*”)

**AND**

(DE "Psychotherapy" OR DE "Child Psychotherapy" OR DE "Play Therapy" OR DE "Behavior Therapy" OR DE "Aversion Therapy" OR DE "Conversion Therapy" OR DE "Dialectical Behavior Therapy" OR DE "Exposure Therapy" OR DE "Implosive Therapy" OR DE "Reciprocal Inhibition Therapy" OR DE "Response Cost" OR DE "Systematic Desensitization Therapy" AND DE "Cognitive Behavior Therapy" OR DE "Cognitive Therapy" OR DE "Group Psychotherapy" OR DE "Neurotherapy" OR psychotherap* OR “psychological therap*” OR “psychological intervent*” OR psychoeduca* OR mentoring* OR coaching* OR mindful* OR relax*)

OR

((parent* OR mother OR father OR teacher* OR school*)

*AND*

(program* OR train* OR educa* OR therapy* OR therapies* OR therapeu*OR intervention* OR coaching* OR counseling*))

OR

((behavio* OR cognit* OR ‘acceptance and commitment’ OR dialectica*)

*AND*

(program* OR therapy* OR therapies* OR therapeu* OR intervention* OR treatment* OR train*))

OR

(psychosocial

AND

(treatment* OR therapy* OR therapies* OR therapeu* OR train* OR intervention* OR program*))

OR

((skill* OR organization* OR organisation* OR planning* OR play*)

AND

(train* OR intervention* OR program*))

**AND**

(AG (childhood OR adolescence) OR (child* OR school* OR infan* OR adolescen* OR pediatri* OR paediatr* OR boy OR boys OR boyhood OR girl OR girls OR girlhood OR youth OR youths OR teen OR teens OR teenage* OR puberty OR preschool* OR toddler* OR juvenile* OR kids))

**AND**

(“control group*” OR random* OR "controlled trial" OR "controlled study" OR "experimental study" OR "experimental design" OR TI trial)

**EBSCO CINAHL**

(MH "Attention Deficit Hyperactivity Disorder" OR "Attention Deficit Disorder with Hyperactivity" OR ADHD OR ADD OR “attention deficit*” OR hyperactiv* OR hyperkinetic* OR “minimal brain deficit*” OR “minimal brain dysfunction*”)

**AND**

(MH "Psychotherapy+" OR psychotherap* OR “psychological therap*” OR “psychological intervent*” OR psychoeduca* OR mentoring* OR coaching* OR mindful* OR relax*)

OR

((parent* OR mother OR father OR teacher* OR school*)

*AND*

For peer review only

(program* OR train* OR educa* OR therapy* OR therapies* OR therapeu*OR intervention* OR coaching* OR counseling*))
OR
((behavio* OR cognit* OR 'acceptance and commitment' OR dialectica*)
*AND*
(program* OR therapy* OR therapies* OR therapeu* OR intervention* OR treatment* OR train*))
OR
(psychosocial
AND
(treatment* OR therapy* OR therapies* OR therapeu* OR train* OR intervention* OR program*))
OR
((skill* OR organization* OR organisation* OR planning* OR play*)
AND
(train* OR intervention* OR program*))

**AND**

(AG child OR (child* OR school* OR infan* OR adolescen* OR pediatri* OR paediatr* OR boy OR boys OR boyhood OR girl OR girls OR girlhood OR youth OR youths OR teen OR teens OR teenage* OR puberty OR preschool* OR toddler* OR juvenile* OR kids))

**AND**

(MH "Clinical Trials+" OR "control group*" OR random* OR "controlled trial" OR "controlled study" OR "experimental study" OR "experimental design" OR TI trial)

**EBSCO ERIC**

(DE "Attention Deficit Disorder" OR DE "Attention Deficit Hyperactivity Disorder" OR ADHD OR ADD OR "attention deficit*" OR hyperactiv* OR hyperkinetic* OR "minimal brain deficit*" OR "minimal brain dysfunction*")

**AND**

(DE "Psychotherapy" OR psychotherap* OR "psychological therap*" OR "psychological intervent*" OR psychoeduca* OR mentoring* OR coaching* OR mindful* OR relax*)
OR
((parent* OR mother OR father OR teacher* OR school*)
*AND*
(program* OR train* OR educa* OR therapy* OR therapies* OR therapeu*OR intervention* OR coaching* OR counseling*))
OR
((behavio* OR cognit* OR 'acceptance and commitment' OR dialectica*)
*AND*
(program* OR therapy* OR therapies* OR therapeu* OR intervention* OR treatment* OR train*))
OR
(psychosocial
AND
(treatment* OR therapy* OR therapies* OR therapeu* OR train* OR intervention* OR program*))
OR
((skill* OR organization* OR organisation* OR planning* OR play*)
AND

For peer review only

(train* OR intervention* OR program*))
**AND**

(child* OR school* OR infan* OR adolescen* OR pediatri* OR paediatr* OR boy OR boys OR boyhood OR girl OR girls OR girlhood OR youth OR youths OR teen OR teens OR teenager* OR puberty OR preschool* OR toddler* OR juvenile* OR kids))
**AND**

("control group*" OR random* OR "controlled trial" OR "controlled study" OR "experimental study" OR "experimental design" OR TI trial)

**Web of Science**
(TS=("Attention Deficit Disorder with Hyperactivity" OR ADHD OR ADD OR "attention deficit*" OR hyperactiv* OR hyperkinetic* OR "minimal brain deficit*" OR "minimal brain dysfunction*"))
**AND**

(TS=(psychotherap* OR psychotherap* OR "psychological therap*" OR "psychological intervent*" OR psychoeduca* OR mentoring* OR coaching* OR mindful* OR relax*
OR
((parent* OR mother OR father OR teacher* OR school*)
*AND*
(program* OR train* OR educa* OR therapy* OR therapies* OR therapeu*OR intervention* OR coaching* OR counseling*))
OR
((behavio* OR cognit* OR 'acceptance and commitment' OR dialectica*)
*AND*
(program* OR therapy* OR therapies* OR therapeu* OR intervention* OR treatment* OR train*))
OR
(psychosocial
AND
(treatment* OR therapy* OR therapies* OR therapeu* OR train* OR intervention* OR program*))
OR
((skill* OR organization* OR organisation* OR planning* OR play*)
AND
(train* OR intervention* OR program*))))
**AND**
(TS=(child* OR school* OR infan* OR adolescen* OR pediatri* OR paediatr* OR boy OR boys OR boyhood OR girl OR girls OR girlhood OR youth OR youths OR teen OR teens OR teenager* OR puberty OR preschool* OR toddler* OR juvenile* OR kids))
**AND**
(TS=("clinical trial*" OR "control group*" OR random* OR "controlled trial" OR "controlled study" OR "experimental study" OR "experimental design"))

For peer review only

**Cochrane Central register of Controlled Trials** *(CENTRAL)*
("Attention Deficit Disorder with Hyperactivity" OR ADHD OR ADD OR "attention deficit*" OR hyperactiv* OR hyperkinetic* OR "minimal brain deficit*" OR "minimal brain dysfunction*")
**AND**

((psychotherap* OR "psychological therap*" OR "psychological intervent*" OR psychoeduca* OR mentoring* OR coaching* OR mindful* OR relax*)
OR
((parent* OR mother OR father OR teacher* OR school*)
*AND*
(program* OR train* OR educa* OR therapy* OR therapies* OR therapeu*OR intervention* OR coaching* OR counseling*))
OR
((behavio* OR cognit* OR 'acceptance and commitment' OR dialectica*)
*AND*
(program* OR therapy* OR therapies* OR therapeu* OR intervention* OR treatment* OR train*))
OR
((psychosocial)
AND
(treatment* OR therapy* OR therapies* OR therapeu* OR train* OR intervention* OR program*))
OR
((skill* OR organization* OR organisation* OR planning* OR play*)
AND
(train* OR intervention* OR program*)))
**AND**

(child* OR school* OR infan* OR adolescen* OR pediatri* OR paediatr* OR boy OR boys OR boyhood OR girl OR girls OR girlhood OR youth OR youths OR teen OR teens OR teenage* OR puberty OR preschool* OR toddler* OR juvenile* OR kids)

For peer review only

Supplement 2. Data sharing manual

## Data collection form

## Content

**Introduction**
**How do I transfer my data?**
**Which participants do we need?**
**Which data do we need?**
**What will we do with the data?**
**Supplement 3A. Measures**

Thank you for taking place in our Psychosocial ADHD INTerverntions (PAINT) collaborators group. We are very grateful that you are willing to put your time into this project. We believe that this group will be fruitful and successful collaboration with the aim to get more insight into psychosocial interventions for ADHD.
With this manual, we hope to provide you with guidance on the data collection. However, if you have any questions regarding the data handling, despite reading this manual, please let us know so we can help you further, and adapt our manual accordingly. You can reach us at paint@accare.nl.

Most of the choices we have made concerning data collection are driven by a need to keep methods and measures similar across trials. Every study is designed slightly different, with different measures, different in- and exclusion criteria, different methods to handle missing data, etc..
To harmonize measures and methods, at some point we will have to make choices regarding data analysis, that differ from the choices made in the original study. For example, we will do intention to treat analysis, while it may be possible that you did not do this in your original analysis. This means that, if available, we will ask for data or participants not included in the original analysis. Furthermore, it is important to mention that we will not report on results from a single trial, although we can report a forestplot displaying all effect sizes of included studies.

### How do I transfer my data?

Preferably, we would receive the information electronically. We can work with any kind of data file (STATA, SPSS, etc.), but we prefer a layout, where each line represents a subject.

You can email the data file once you encrypted it using a program such as 7-zip (an explanation on how to do this is included in supplement B). It is important that the password with which you will protect your data file, will be send in a separate email from the email that contains the data file (as attachment). Another important point in transferring the data is that the data should be anonymized. This means that there is no information with which we could trace a single participant back to the individual. This means that no names should be included in the file. However, all other information that can identify a person should be left out of this file. This can mean combinations of variables, for example postal code and date of birth. Please make sure that this kind of information is not present in the data file. If these kinds of examples arise, please contact us, so we can think with you on what best to do.

### Which participants do we need?

We want the data of all included participants. This means all participants that have a baseline measure and were randomized. It could be possible that this is a different approach than you took in

For peer review only

your analysis of the data. Because we want to perform intention to treat analysis, we need all data from included participants.

Which data do we need?

We ask you for clean data (please contact us if this provides you with any questions). We want sum scores and if available norm-scores of questionnaires. Proper labeling is important. In the Table below we provide you with an overview of constructs that we are interested in. It could be possible that the measure or instrument used in your study is not mentioned in this Table, this does not mean that we are not interested, the measures mentioned are mere examples. If there are any remarks on this measure (e.g. certain items were not used) please mention these in the column "remarks". Please indicate which informants filled out the questionnaires, when multiple informants are used please provide us with data from all informants. If multiple instruments have been used to measure a certain construct, we would really like to receive all instruments (for example a self and a proxy version, or multiple ADHD measures). This could aid us in the harmonization process (see above). If a construct is measured with an in-house instrument or if you made any adjustments on the instrument, could you also provide us this instrument?

For questionnaires, we would like to receive the, sum scores and norm-scores and if available item scores. We would always like to receive the full questionnaire if possible, and not just certain subscales. In the future, it could be possible that we will ask you about norming tables, we hope however, that we will not need these.

For (structured) interviews we would like to receive items scores, sum scores and outcomes. We would like to receive information on the full interview, not only certain outcomes.

Concerning cognitive measures, we would like to receive calculated outcomes variables across trials and conditions, not mere trial to trial performance. For example, in the stop task, we would like to receive measures such as SSRT, mean (and SD) of reaction time on Go trials, number of commissions and omissions, but not the individual performance on a single trial of the stop task. We fill also need information about the task such as number of conditions, number of trials, and how the outcome variables were calculated.

For variables that are available on multiple time points, please provide us with all relevant data. For example, if an ADHD measure is taken at baseline, right after the intervention and after 3 months, we would like to receive this measure assessed at all time points. Again, proper labelling is very important. Please label the data in English (or Dutch).

The constructs and variables that we are interested in for the IPD meta-analysis are shown below. We understand that not all studies have included all measures. The table below will provide you with a list you can keep on hand when preparing the data before sending it. Next to the constructs are examples of instruments. Please remember that this is not a complete list, and that it could be possible that you have used an instrument that is not in our list, but that would still be very valuable for this IPD meta-analysis. Please remember that you do not have to recode your data. We will do this for all datasets.

What will we do with the data?

Once we receive the data we will do some basic data checks. These will include checking missing and impossible values. If we will have any questions concerning the data during or after these checks we will contact you for clarification. After that we will harmonize the data with the other datasets. This harmonization process depends on the available data. For example, this could mean that we have to dichotomize data, or making norm deviation scores (translating raw questionnaire scores on one

For peer review only

scale into raw questionnaire scores on another scale using norm scores). Harmonization is easiest if the same measure has been used across studies. If you have used multiple instruments to measure a certain outcome (e.g. more than 1 ADHD scale), we ask you to provide us both measures. This could mean that we may be able use a latent variable for some measures, or we could calculate correlations between measures.

The original dataset will be saved as the original file. Any harmonization will take place in a separate file, which are only later merged into one big data file.

For peer review only

Supplement 2A.

Tabel 1 Measures.

| **Descriptive measures** | |
|---|---|
| Participant ID | |
| Date of randomization | Dd/mm/yyyy |
| Date of treatment start | Dd/mm/yyyy |
| Date post-intervention measurement | Dd/mm/yyyy |
| Date follow up measurement | Dd/mm/yyyy |
| Date baseline measurement | Dd/mm/yyyy |
| Treatment allocation | 0- Control<br>1- Intervention |
| Age | Age in years |
| Gender | 1-Male<br>2-Female |
| IQ | Norm score of WISC |
| Verbal IQ | Norm score of WISC |
| Performance IQ | Norm score of WISC |
| Ethnicity | |
| Socioeconomic status | |
| ADHD severity at baseline | |
| Comorbidities | ODD/CD/Anxiety/Depression/PDD/Tics/ etc. |
| Number of children living with the family | Total number of children in the household |
| Primary care-taker education | |
| Secundary care-taker education | |
| Household income | Combined income of primary caretaker and (if applicable) secundary caretaker |
| Parental psychopathology (diagnosis) | Parental ADHD/ anxiety/depression/ etc |
| Marital status | |
| Education primary caretaker | |
| Education secondary caretaker | |
| Age Father | Age in years |
| Age mother | Age in years |

For peer review only

| | |
|---|---|
| Psychoactive Medication use pre- intervention | Yes/no, if yes which medication, mg MPH if applicable, x times per day, for the last month, duration |
| Previous care use of the child | Care use in mental health. |
| Medication use during intervention | Yes/no, if yes, mg per day |
| Treatment expectancy | |
| Stable medication use during intervention | Yes/No/NA |
| Other descriptive measures | |
| **Individual child outcomes- behavioral** | |
| **Measured constructs** | **Examples of instruments** |
| **ADHD** | Conners, CBCL, DBD, ACTeRS, ARS, BASC, SNAP, SWAN, Werry Weiss Peters activity scale, Behavioral checklist, PACS, Parent daily report, KSADS, standardized behavioral observations |
| Disruptive disorders/ behavior | Conners, CBCL, DBD, EDBI, SNAP, SSRS, ACTeRS, KSADS, behavior problem checklist, aggression and conduct problems scale, behavioral checklist, PACS, parent daily reports, standardized behavioral observations |
| Social skills | DSAS, Behavior problem checklist, BASC, ACTeRS, SSRS, CBCL |
| Internalizing problems | Conners, CBCL, SSRS, BASC, Behavior problem checklist, MASC, PACS, KSADS, SCARED, CDI |
| Attachment | |
| Quality of life | HONOSCA, kidsscreen, EQ-5d, IRS |
| Selfconcept | Piers-Harris |
| Which other child behavioral outcomes are available? | |
| **Individual child outcomes- academic outcomes** | |
| Measured Construct | Examples of measures |
| Grades on core courses | Math, Spelling, Reading |
| Standardized Academic Achievement tests | Reading, spelling, math scores, woodcock johnson, WRAT, WISC Durrel |
| On and off task behaviors | Classroom observations, SOAPs |
| Academic competence | Academic rating scale, classroom performance survey |

For peer review only

| | |
|---|---|
| Which other academic outcomes are available? | |
| **Individual child outcomes- cognitive outcomes[a]** | |
| Higher cognitive functions | Working memory, executive functions, inhibition, planning, set shifting, reward sensitivity, emotion regulation |
| Intellectual functioning | IQ (WISC, RAVEN) |
| Motor skills | Fine motor skills, precision, gross motor skills |
| Memory | Visuospatial, verbal |
| Basic cognitive functions | Attention, reaction time, speed, perception |
| **Child individual outcomes- other measures** | |
| Adverse events | Types of adverse events reported |
| Treatment fidelity | |
| Treatment satisfaction | |
| Number of sessions attended | |
| Drop out | Yes/no |
| Date of discontinuation of the intervention | |
| Reason for discontinuation | |
| **Parental outcome measures** | |
| Measured constructs | Examples of measures |
| Parental psychopathology | DASS, GHQ, BDI, PSI, CSS, AARS, SCL90, |
| Parent child interaction, parenting behaviors, parenting style, parenting affect | DPICS or other observational measure, parenting practices inventory (PPI), parent interaction style, PS (parenting scale), measures parental sensitivity/reponsivity |
| Parental competence/ self efficacy | PSI-subscale parental competence, PSOC |
| Spouse relationship | locke wallace marital adjustment, PSI-subscale |
| Parental self esteem | Rosenberg self esteem scale (RSE) |
| Parenting stress | PSI |
| Which other parenting outcomes are available? | |

[a] concerning cognitive measures, we would like to receive calculated outcomes measures, not mere trial to trial performance. For example, in the stop task, we would like to receive measures such as SSRT, mean reaction time, number of commissions and omissions, but not the individual performance on a single trial of the stop task. We will also need information on the conditions of the task, how the outcome was calculated, etc.

For peer review only

PRISMA-P (preferred reporting items for systematic review and meta-analysis protocols) 2015 checklist: recommended items to address in a systematic review protocol

| Section and topic | Item No | Checklist item | Location in manuscript |
|---|---|---|---|
| **Administrative information** | | | |
| Title: | | | |
| Identification | 1a | Identify the report as a protocol of a systematic review | Page 1 |
| Update | 1b | If the protocol is for an update of a previous systematic review, identify as such | NA |
| Registration | 2 | If registered, provide the name of the registry (such as PROSPERO) and registration number | Page 8 |
| Authors: | | | |
| Contact | 3a | Provide name, institutional affiliation, e-mail address of all protocol authors; provide physical mailing address of corresponding author | Page 1 |
| Contributions | 3b | Describe contributions of protocol authors and identify the guarantor of the review | Page 2 |
| Amendments | 4 | If the protocol represents an amendment of a previously completed or published protocol, identify as such and list changes; otherwise, state plan for documenting important protocol amendments | NA |
| Support: | | | |
| Sources | 5a | Indicate sources of financial or other support for the review | Page 2 |
| Sponsor | 5b | Provide name for the review funder and/or sponsor | Page 2 |
| Role of sponsor or funder | 5c | Describe roles of funder(s), sponsor(s), and/or institution(s), if any, in developing the protocol | Page 2 |
| **Introduction** | | | |

| Section and topic | Item No | Checklist item | Location in manuscript |
|---|---|---|---|
| Rationale | 6 | Describe the rationale for the review in the context of what is already known | Page 5-8 |
| Objectives | 7 | Provide an explicit statement of the question(s) the review will address with reference to participants, interventions, comparators, and outcomes (PICO) | Page 8 |
| **Methods** | | | |
| Eligibility criteria | 8 | Specify the study characteristics (such as PICO, study design, setting, time frame) and report characteristics (such as years considered, language, publication status) to be used as criteria for eligibility for the review | Page 9 |
| Information sources | 9 | Describe all intended information sources (such as electronic databases, contact with study authors, trial registers or other grey literature sources) with planned dates of coverage | Page 9 |
| Search strategy | 10 | Present draft of search strategy to be used for at least one electronic database, including planned limits, such that it could be repeated | Page 9 (and supplement) |
| Study records: | | | |
| Data management | 11a | Describe the mechanism(s) that will be used to manage records and data throughout the review | Page 10 |
| Selection process | 11b | State the process that will be used for selecting studies (such as two independent reviewers) through each phase of the review (that is, screening, eligibility and inclusion in meta-analysis) | Page 9 |
| Data collection process | 11c | Describe planned method of extracting data from reports (such as piloting forms, done | Page 8-9 (and supplement) |

| Section and topic | Item No | Checklist item | Location in manuscript |
|---|---|---|---|
| | | independently, in duplicate), any processes for obtaining and confirming data from investigators | |
| Data items | 12 | List and define all variables for which data will be sought (such as PICO items, funding sources), any pre-planned data assumptions and simplifications | Page 10-12 (and supplement) |
| Outcomes and prioritization | 13 | List and define all outcomes for which data will be sought, including prioritization of main and additional outcomes, with rationale | Page 10-12 |
| Risk of bias in individual studies | 14 | Describe anticipated methods for assessing risk of bias of individual studies, including whether this will be done at the outcome or study level, or both; state how this information will be used in data synthesis | Page 12 |
| Data synthesis | 15a | Describe criteria under which study data will be quantitatively synthesised | Page 12-13 |
| | 15b | If data are appropriate for quantitative synthesis, describe planned summary measures, methods of handling data and methods of combining data from studies, including any planned exploration of consistency (such as $I^2$, Kendall's τ) | Page 12-13 |
| | 15c | Describe any proposed additional analyses (such as sensitivity or subgroup analyses, meta-regression) | Page 12-13 |
| | 15d | If quantitative synthesis is not appropriate, describe the type of summary planned | NA |
| Meta-bias(es) | 16 | Specify any planned assessment of meta-bias(es) (such as publication bias | Page 13 |

| Section and topic | Item No | Checklist item | Location in manuscript |
|---|---|---|---|
| | | across studies, selective reporting within studies) | |
| Confidence in cumulative evidence | 17 | Describe how the strength of the body of evidence will be assessed (such as GRADE) | Page 12 |

For peer review only
